# The nBAF complex subunit CREST/SS18L1 regulates hippocampal memory processes via tyrosine 397 and histone acetyltransferase CBP

**DOI:** 10.1016/j.celrep.2026.117158

**Published:** 2026-03-25

**Authors:** Franklin G. Garcia, Maria Farias de Albuquerque, Vanessa Johnson, Enikö Kramár, Kousha Changizi, Sherif Abdelkarim, Agatha S. Augustynski, Dina P. Matheos, Joseph Picone, Tracy L. Fetterly, Alyssa Rodriguez, Jessica Childs, Angela Gomez-Arboledas, Julia Winter, Elizabeth Heller, Pierre Baldi, Marcelo A. Wood

**Affiliations:** 1 Department of Neurobiology and Behavior, Charlie Dunlop School of Biological Sciences, University of California, Irvine, Irvine, CA 92697, USA; 2 Center for the Neurobiology of Learning and Memory, University of California, Irvine, Irvine, CA 92697, USA; 3 Department of Computer Science, Institute for Genomics and Bioinformatics, University of California, Irvine, Irvine, CA 92697, USA; 4 Department of Systems Pharmacology & Translational Therapeutics, Perelman School of Medicine, University of Pennsylvania, Philadelphia, PA 19104, USA; 5 Institute for Memory Impairments and Neurological Disorders, University of California, Irvine, Irvine, CA, USA; 6 Lead contact

## Abstract

Chromatin-modifying and -remodeling machineries are important for learning-induced transcriptional activity, yet it remains unclear how they coordinate to drive *de novo* gene expression for memory formation. Here, we examine the transcription factor known as calcium-responsive transactivator (CREST) in memory formation, synaptic plasticity, and learning-induced gene expression. CREST is known to bind major chromatinmodifying and -remodeling machineries via interaction with CREB-binding protein (CBP) and brahma-related gene 1 (BRG1), respectively. *In silico* modeling of CREST identified tyrosine 397 (Y397) within the CBP-binding domain. Expression of a CREST Y397F point mutant impairs long-term potentiation and memory. Conversely, expression of a CREST Y397D point mutant enhances memory in a CBP-dependent manner. Differential gene expression analysis reveals distinct CREST Y397-regulated signatures during memory consolidation. CBP acts through CREB and post-translation modifications to affect memory, but the findings of this study argue for consideration of the CREST-CBP interaction and Y397 accessibility as factors in memory processes.

## In brief

Garcia et al. show that knockdown of CREST or expression of CREST Y397F in the adult dCA1 impairs hippocampal long-term memory formation and synaptic plasticity. Bulk RNA sequencing and differential gene expression analysis were used to identify distinct gene sets that are regulated by CREST Y397 during memory consolidation.

## Graphical Abstract

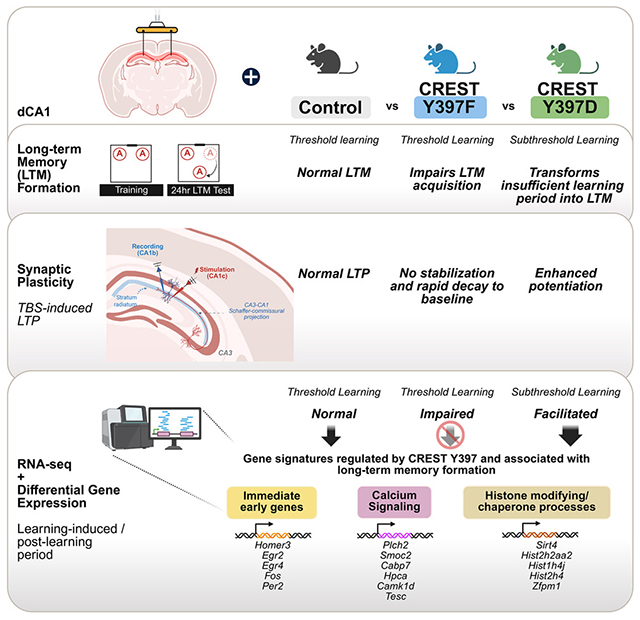

## INTRODUCTION

The process by which new information is converted into memory relies on a highly coordinated induction of molecular, cellular, and transcriptional changes. The stabilization process of memory consolidation requires distinct profiles of *de novo*, learning-induced gene expression during the critical “post-training” phase of a learning event that is necessary for transforming memory into a more lasting form (i.e., long-term memory, LTM).^1^ While it is well accepted that transcription is necessary for memory consolidation, it is not well understood how transcriptionally permissive mechanisms cooperate to induce gene expression required for memory formation. The epigenome provides a particularly powerful signal-integration system in the brain for transcriptional machinery to effectively access gene targets within the condensed higher-order structure of chromatin and act on the nucleosome subunits that consist of condensed DNA and histone octamers. The study of epigenetic mechanisms has identified major roles for chromatin modifying (post-translational modification on histone proteins) and remodeling (nucleosome sliding, evicting, or shuffling) in LTM consolidation.^2–6^ The acetylation and deacetylation of lysine residues on histone tails is a dynamic and reversible chromatin modification that is regulated by histone acetyltransferases (HATs) and histone deacetylases (HDACs).^4,7,8^

Very little is known about the transcriptional mechanisms that bring together histone modification and nucleosome remodeling. The discovery of calcium-responsive transactivator (CREST; aka synovial sarcoma translocation gene on chromosome 18-like 1, SS18L1) provided a key candidate for a transcriptional regulator with the capacity to interact with multiple subunits of the chromatin-modifying/remodeling machinery.^9^ There is an N-terminal regulatory domain that is essential for binding brahma-related gene 1 (BRG1), which is an ATPase of the neuron-specific Brg/Brm-associated factor (nBAF) nucleosome remodeling complex. CREST also has a C-terminal transactivation domain that is necessary for binding and recruiting cAMP-responsive element binding protein (CREB)-binding protein (CBP), which is a HAT and transcriptional regulator.^9–11^ CBP is well known for its role in long-term potentiation (LTP) and memory via its phosphorylation-dependent interaction with CREB, an essential transcription factor for memory formation.^12–17^ Mutations in CREST have been associated with amyotrophic lateral sclerosis (ALS), a fatal neurodegenerative disorder characterized by loss of muscle control and cognitive dysfunction.^18–20^ The role of CREST in cognition independent of its function in neural motor activity remains unknown yet a strong possibility, given its interaction with CBP.

Previous work by our group and others has demonstrated a role for *Crest* in cocaine-induced drug-seeking behavior and synaptic plasticity in the nucleus accumbens, neuronal dendrite morphogenesis, and the assembly kinetics of the neuron-specific nBAF nucleosome remodeling complex in post-mitotic neurons.^9,21,22^ A study by Ghosh and colleagues first identified that mutation of *Crest* in cultured hippocampal neurons resulted in decreased dendrite length and morphology, suggesting a role for CREST in cellular mechanisms important for synaptic plasticity and memory.^9^ In a different study by Qiu and Ghosh, it was shown that a CREST:BRG1 complex can occupy the promoter region of glutamate ionotropic receptor NMDA-type subunit 2B (*Nr2b*) in stimulated cultured neurons and recruit CBP for increased *Nr2b* mRNA expression in a CREST-dependent manner.^10,23^ In this study, we examined the role of CREST in LTP (a form of synaptic plasticity) and memory.

## RESULTS

### Crest is necessary for LTM formation and stabilization of LTP in the adult dorsal hippocampus

**Figure 1.**
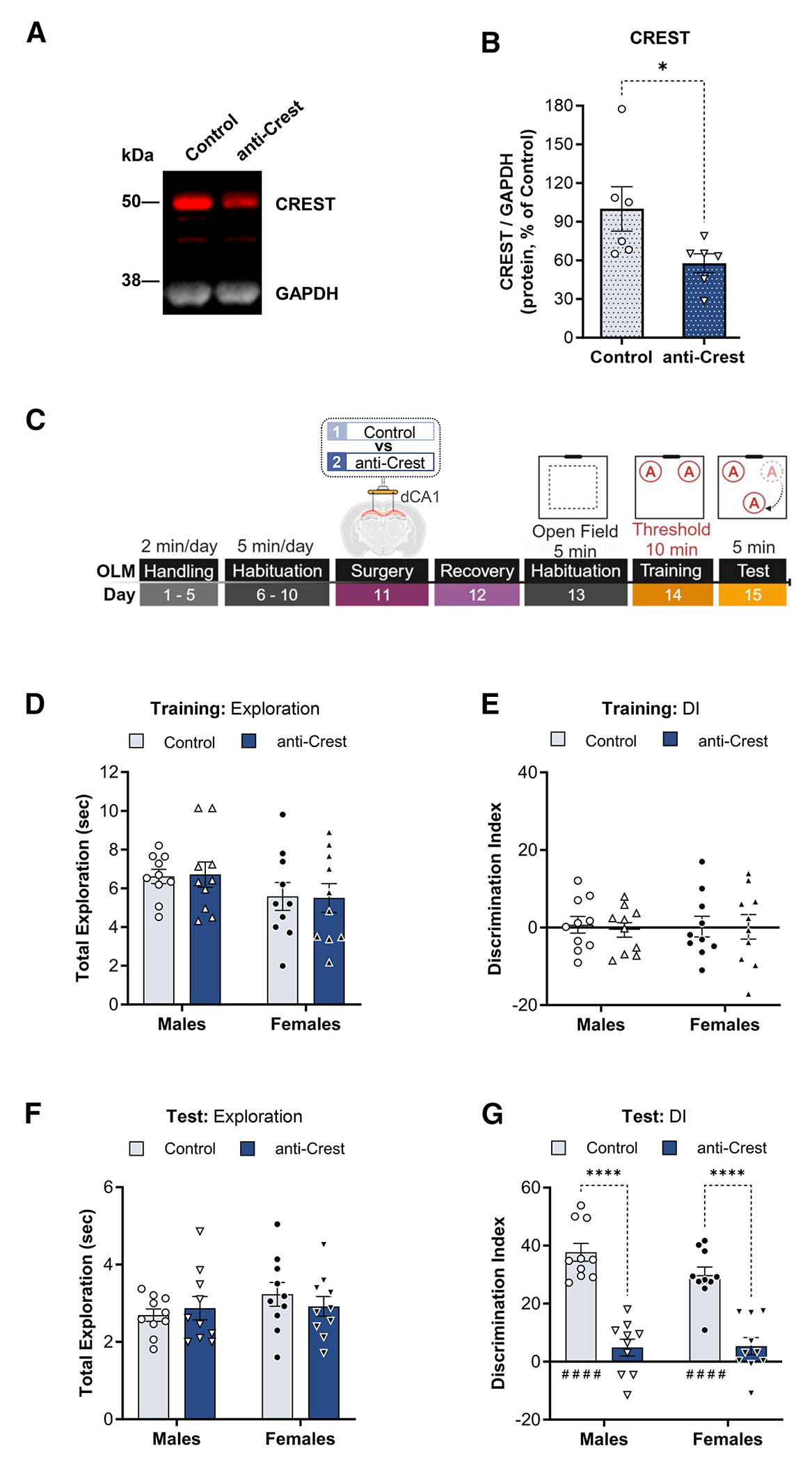
Focal knockdown of *Crest* in adult dorsal hippocampus impairs long-term memory formation (A and B) Representative immunoblot and quantification of CREST (red) protein expression relative to GAPDH in the adult dorsal hippocampus (dCA1) 4 days after infusion of a control or anti-*Crest* vivo-morpholino oligo (two-tailed Student’s *t* test*: t*_10_ = 2.25, *p < 0.05). *n* = 6 males/condition (see Data S1 source data). Protein size markers are annotated in units of kilodaltons. (C) Timeline of OLM behavior testing with bilateral infusion of either control or anti-*Crest* vivo-morpholino oligo into the dCA1. (D) Total exploration of objects during the threshold training session (10 min) (two-way ANOVA: sex × treatment effect *F*_1,36_ = 0.020, *p* = 0.89). (E) Discrimination index (DI) for threshold training session (10 min) (two-way ANOVA: sex × treatment effect *F*_1,36_ = 0.066, *p* = 0.80). (F) Total exploration of objects during test session (5 min) (two-way ANOVA: sex × treatment effect *F*_1,36_ = 0.88, *p* = 0.35). (G) DI for test session (5 min) at 24 h after training session (two-way ANOVA: effect of treatment *F*_1,36_ = 94.99, ****p < 0.0001). Sidak’s *post hoc* test of control vs. anti-*Crest*: males, ****p < 0.0001, and females, ****p < 0.0001. No effect of sex × treatment interaction (two-way ANOVA: *F*_1,36_ = 2.02, *p* = 0.16). Pre-planned paired *t* test of test × training session annotated for each cohort: controls, males, *t*_9_ = 16.66, ^####^*p* < 0.0001, and females, *t*_9_ = 9.81, ^####^*p* < 0.0001; and anti-*Crest*, males, *t*_9_ = 1.40, *p* = 0.19, and females, *t*_9_ = 1.03, *p* = 0.33. Males, white circles and triangles. Females, black circles and triangles. Data are presented as means ± SEM. Data represent two independent cohorts of male and female C57BL/6J adult (*n* = 10/condition/sex). See also Figure S2

The first goal of this study was to determine the role of *Crest* in LTM formation and LTP. We chose the object location memory (OLM) task and slice physiology, which both involve the dorsal CA1 (dCA1) region of the hippocampus.^24–32^ Immunofluorescences staining shows CREST has robust expression among the neuronal (NeuN+) subtype population within the dorsal hippocampus (Figure S1). As such, we targeted the dCA1 with an anti-*Crest* morpholino to knockdown CREST expression (Figure S2A). We found that 4 days is sufficient to significantly reduce protein levels of CREST (Figures 1B and S2B). To determine the effect of knocking down CREST on memory, male and female C57BL/6J mice were subjected to the OLM task and received either control or anti-Crest morpholino via bilateral infusion 24 h after habituation (see schematic, Figure 1C). A comparison between control and anti-*Crest* morpholino conditions for distance traveled throughout days of habituation prior to surgery and after delivery of the morpholino showed no significant difference (Figure S2C, males and females). As we have previously reported, a 10-min training session (which we will refer to as threshold training) is sufficient for young adult mice to develop an LTM for object location.^27,28,30^ During the threshold training session, we did not observe a significant difference in total object exploration time or object preference (i.e., discrimination index [DI]) in either group condition or sex (Figures 1D and 1E). At 24 h after training, control morpholino-infused male and female groups showed a significant increase in preference for the moved object relative to the training session (paired Student’s *t* test of test × training session: control, males, *t*_9_ = 16.66, ^####^*p* < 0.0001, and females, *t*_9_ = 9.81, ^####^*p* < 0.0001) (Figures 1F and 1G). In contrast, male and female animals infused with the anti-*Crest* morpholino did not show a significant preference (relative to the training session) for the moved object during testing (paired Student’s *t* test of test × training: anti*Crest*, males, *t*_9_ = 1.40, *p* = 0.19, and females, *t*_9_ = 1.03, *p* = 0.33) despite having similar levels of total object exploration (Figures 1F and 1G). There were no significant sex differences between the independent male and female cohorts in OLM performance (no effect of sex × treatment interaction: *F*_1,36_ = 2.02, *p* = 0.16; Sidak’s *post hoc* test for male vs. female, control, *p* = 0.14, and anti-*Crest*, *p* = 0.99). Thus, animals infused with the anti-*Crest* morpholino were impaired in LTM acquisition/consolidation in a hippocampus-dependent task.

We also assessed retention of normal mouse anxiety behavior (thigmotaxis) via open field and elevated-plus-maze testing (Figure S2D).^33–35^ Analysis of zone preference (open field, i.e., center vs. edges of OLM chambers) on day 13 (i.e., 2 days post-morpholino infusion) of OLM behavior and exploration in the elevated plus maze (1–2 days after OLM test) showed that focal knockdown of *Crest* expression in the hippocampus did not alter animal thigmotaxis across control and anti-*Crest* conditions of either male or female groups (Figures S2E and S2F).

**Figure 2.**
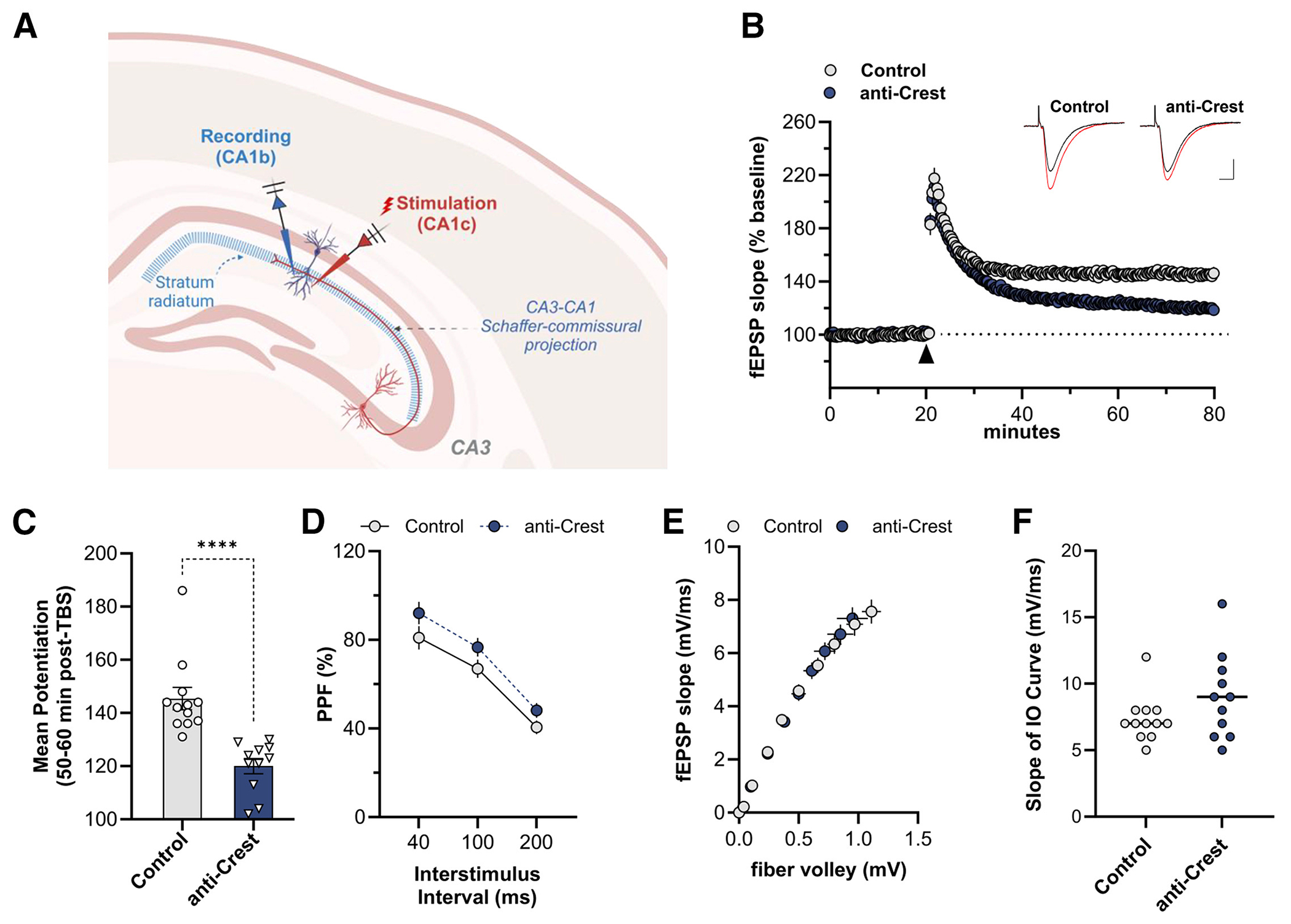
Focal knockdown of *Crest* in the adult hippocampus impairs synaptic plasticity (A) A simplified schematic of electrode placement in a hippocampus slice. Long-term potentiation (LTP) was induced by applying theta burst stimulation (TBS, black arrowhead) at CA1c of Schaffer-collateral projections, and recordings were collected in apical dendrites at CA1b (stratum radiatum) of the dorsal hippocampus. (B) Mean slope of the field excitatory postsynaptic potential (fEPSP) as a percentage of baseline recordings in slices prepared from young (2 months old) C57BL6/J males at 4 days post-bilateral infusion of either control (gray) or anti-*Crest* (blue) vivo-morpholinos. Representative traces during baseline (black) and 60 min post-TBS (red). Scale bar: 1 mV/5 ms. (C) Bar graph shows mean potentiation of control (circles) and anti-*Crest* (downward triangles) infused conditions at 50–60 min after TBS (Student’s *t* test: *t*21 = 4.90, ****p < 0.0001). (D–F) (D) Paired-pulse facilitation (PPF) (two-way ANOVA: no effect of condition × time interaction *F*_2,42_ = 0.67, *p* = 0.52) and (E) input/out (IO) curve and (F) slope of IO curves (*t*21 = 1.58, *p* = 0.13) for control (gray) and anti-*Crest* (blue) groups. Data are presented as means ± SEM. *n* = 6 male mice/condition; 11 or 12 slices/condition.

We next examined LTP, a form of lasting synaptic plasticity that requires gene expression. Transverse hippocampal slices were prepared 4 days post-infusion of either control or anti-*Crest* morpholino, and LTP was induced by delivering theta burst stimulation (TBS) to Schaffer collateral inputs in CA1c. Field excitatory post-synaptic potentials (fEPSPs) were recorded from outputs in CA1b stratum radiatum (Figure 2A, schematic). TBS-induced LTP in slices from the anti-*Crest* morpholino group failed to stabilize with respect to the control morpholino-infused group (Figure 2B). At 50–60 min post-TBS (i.e., 70–80 min after beginning CA1b recording), slices from anti-*Crest* morpholino-infused mice had significantly less potentiation relative to the control morpholino group (Figure 2C). Paired-pulse facilitation (PPF) and input/output curves showed no difference between control and anti-Crest morpholino groups (Figures 2D–2F). Thus, CREST knockdown in the hippocampus impairs LTP. Together, these results demonstrate that focal knockdown of CREST is sufficient to impair the acquisition of long-term object location-dependent memory in both the adult male and the adult female dorsal hippocampus and that CREST is necessary for the stabilization of LTP.

### Crest has a tyrosine within the CBP-binding domain with predictive affinity for post-translational modification

**Figure 3.**
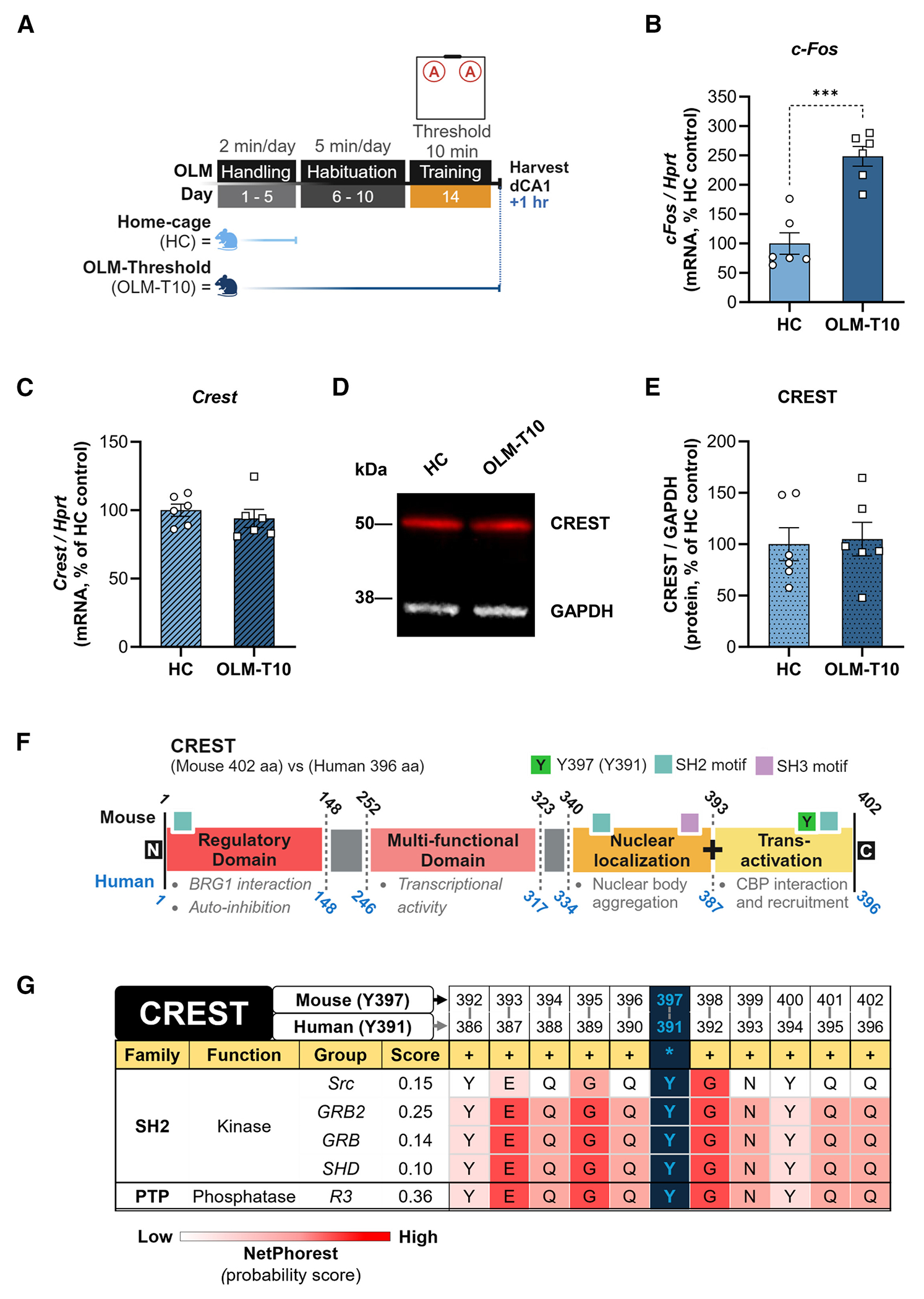
CREST has a highly conserved tyrosine within the CBP-binding domain (A) Timeline of OLM behavior for collection of dCA1 tissue from home cage (HC) or 1 h post-OLM threshold training (OLM-T10) conditions. (B) Quantification of *cFos* mRNA expression relative to *Hprt* reference gene in HC (circles) and OLM-T10 (squares) (Student’s *t* test: *t*_10_ = 5.99, ***p < 0.001). (C) Quantification of *Crest* mRNA expression in HC and OLM-T10 (Student’s *t* test: *t*10 = 0.76, *p* = 0.47). (D and E) Representative immunoblot and quantification of CREST (red) protein expression in HC and OLM-T10 conditions relative to GAPDH (gray, loading control) (Student’s *t* test, *t*_10_ = 0.22, *p* = 0.83) (see Data S1 source data). Protein size markers are annotated in units of kilodaltons. (F) Schematic adapted from the UniProt Consortium database (#O75177, human, and #Q8BW22, mouse) of key domains and motifs and a tyrosine residue in the transactivation (i.e., CBP-binding) domain that is highly conserved between the mouse and the human CREST protein sequences. (G) Summary output adapted from the Net-Phorest2.1 classifier for kinase recognition motifs relative to the conserved CREST Y397 (mouse) and Y391 (human) tyrosine site (blue) with a representative probability score gradient for amino acid residues within the transactivation domain (C terminus) (see Data S1 source data). Data are represented as means ± SEM with *n* = 6/group, all males.

Next, we examined whether *Crest* expression is induced during the memory consolidation phase, which is known to require *de novo* gene expression.^15,16^ As shown in the Figure 3A experimental timeline, the dCA1 was harvested from mice at 1 h after OLM threshold training (OLM-T10) or home cage (HC, control) conditions, as we previously published.^28,30,36^ Quantification of *c-Fos*, a well-characterized learning-induced gene in LTM, mRNA expression showed a nearly 2.5-fold increase in the OLM-T10 group compared with HC controls (Figure 3B, 391 (Y391) in the human CREST peptide sequence is a target for phosphorylation, according to curated mass spectroscopy analysis, which coincides with the homologous Y397 site in mouse (Figure 3F).^40^ Notably, both the human Y391 and the mouse Y397 conserved residues reside within the last nine C-terminal amino acids necessary for recruitment of CBP (Figure 3F).^9,11,41^ Additionally, when we used NetPhorest2.1 (i.e., a computational atlas of Table S1).^37–39^ Quantification of *Crest* mRNA expression in hippocampal CA1 did not show any difference relative to HC control (Figure 3C). Similarly, there were no differences in CREST protein levels between HC and OLM-T10 conditions (Figures 3D and 3E). Thus, neither *Crest* mRNA nor CREST protein levels change during memory consolidation, which is supported by our RNA sequencing (RNA-seq) data from Kwapis et al.^28^ This suggests that CREST may be regulated via a post-translational modification mechanism, such as phosphorylation, similar to the phosphorylation of CREB that is required to recruit CBP.^12^

To identify potential sites on CREST that may be subject to post-translational modification, we utilized the PhosphoSite Plus (Cell Signaling) database, where we found that tyrosine consensus sequence motifs for kinases and binding domains derived from *in vivo* and *in vitro* assays), we found that the last 11 amino acid residues spanning the C terminus (which includes the CBP-binding domain) of CREST have conserved consensus binding motifs recognized by SH2-domain family kinases and PTP family phosphatases (based on prediction score) (Figure 3G and Data S1).^42^ We further cross-referenced the CREST peptide sequence with the PhosphoSVM (a non-kinase-specific prediction tool for serine, threonine, and tyrosine residue phosphorylation) database and found that the human Y391 residue was ranked with the highest prediction score among all other candidate targets for phosphorylation.^43^ Thus, we generated point-mutant versions of CREST.

### Manipulation of CREST Y397 has bidirectional effects on hippocampal CA1-dependent LTM and LTP

**Figure 4.**
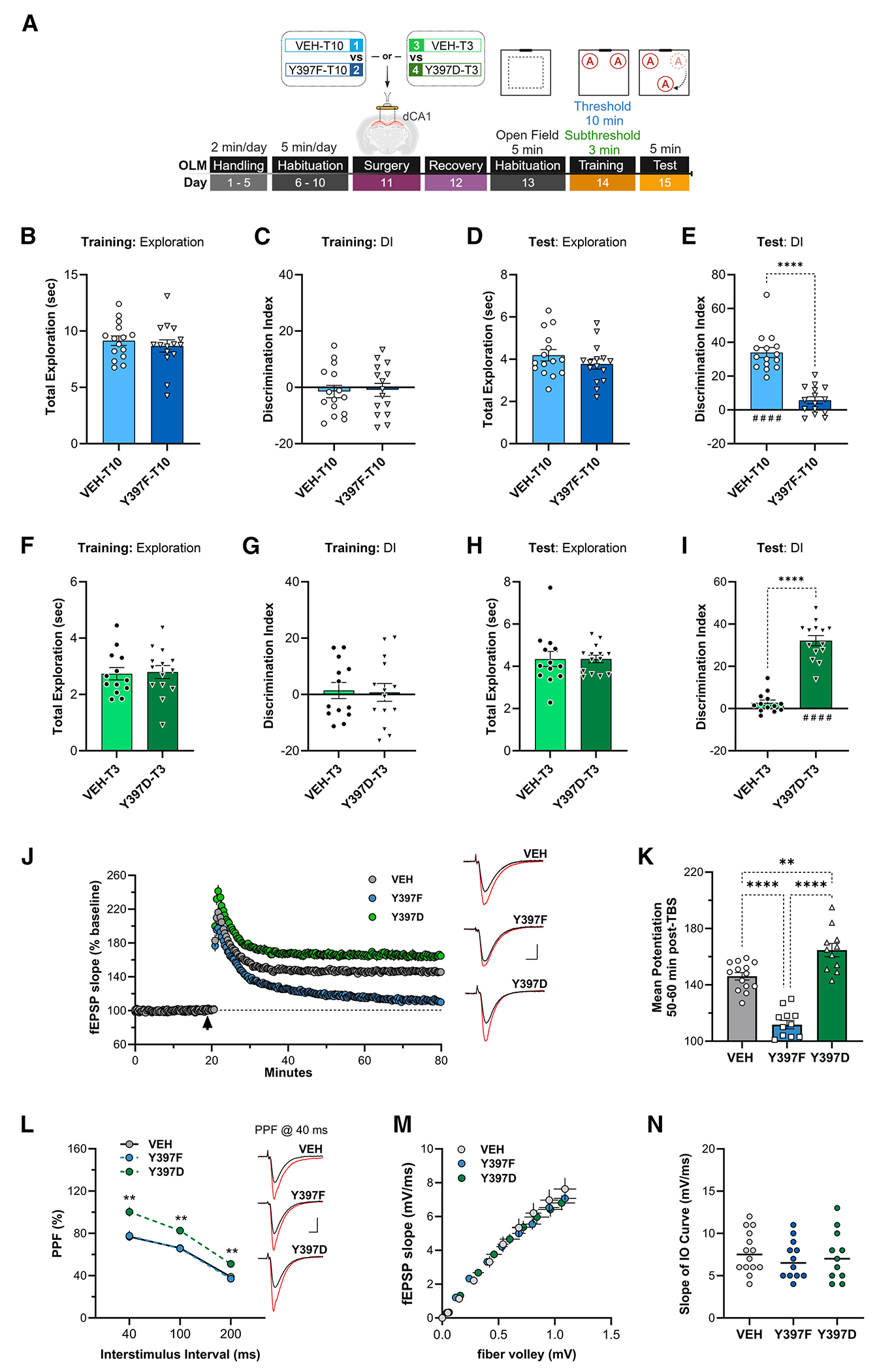
CREST Y397 bidirectionally regulates memory processes of long-term memory formation and synaptic plasticity (A) Timeline of OLM behavior procedure in which in vivo-jetPEI-mediated transfection was used to express CREST Y397F (2, Y397F-T10, B–E), CREST Y397D (4, Y397D-T3, F–I), or vehicle control (1 and 3, VEH-T10 and VEH-T3, respectively) in the adult dCA1 and examined in either a threshold (T10, 10 min) or a subthreshold (T3, 3 min) training condition. (B) Total time of object exploration in 10 min threshold training session for groups VEH-T10 and Y397F-T10 (Student’s *t* test: *t*28 = 0.67, *p* = 0.51). (C) DI in 10 min threshold training session for groups VEH-T10 and Y397F-T10 (Student’s *t* test: *t*28 = 0.19, *p* = 0.85). (D) Total time of object exploration in 5 min OLM test session for groups VEH-T10 and Y397F-T10 (Student’s *t* test: *t*_28_ = 1.14, *p* = 0.26. (E) DI in 5 min OLM test session for groups VEH-T10 and Y397F-T10 (Student’s *t* test: *t*_28_ = 7.76, ****p < 0.0001) (training [C] vs. test DI, pre-planned Student’s *t* test: VEH-T10, *t*_14_= 8.25, ^####^*p* < 0.0001, and Y397F-T10, *t*_14_ = 1.93, *p* = 0.07). (F) Total time of object exploration in 3 min OLM subthreshold training session for groups VEH-T3 and Y397D-T3 (Student’s *t* test: *t*_25_= 0.19, *p* = 0.85). (G) DI in 3 min subthreshold training session for groups VEH-T3 and Y397D-T3 (Student’s *t* test: *t*_25_ = 0.16, *p* = 0.87). (H) Total time of object exploration in 5 min OLM test session for groups VEH-T3 and Y397D-T3 (Student’s *t* test: *t*_25_ = 0.0034, *p* = 0.997). (I) DI in 5 min OLM test session for groups VEH-T3 and Y397D-T3 (Student’s *t* test: *t*_25_ = 10.49, ****p < 0.0001) (training [G] vs. test DI, pre-planned Student’s *t* test: VEH-T3, *t*_12_ = 0.41, *p* = 0.69, and Y397D-T3, *t*_13_ = 5.91, ^####^*p* < 0.0001). Data are presented as means ± SEM. *n* = 13–15/condition, males only. VEH-T10 (white circles) vs. Y397F-T10 (white triangles). VEH-T3 (black circles) vs. Y397D- T3 (black triangles). (J) Mean slope of field excitatory postsynaptic potential (fEPSP) recordings from CA1b stratum radiatum in slices prepared from adult C57BL/6J males at 4 days post-bilateral infusion of in vivo-JetPEI transfection reagent with vehicle-only control (VEH; gray), CREST Y397D (green) or CREST Y397F (blue) experimental conditions. Arrowhead marks TBS. Representative traces during baseline (black) and 60 min post-TBS (red) for each condition (scale bar: 1 mV/5 ms). (K) Quantification of mean ± SEM potentiation of VEH control (white circles), Y397F (white squares), and Y397D (white triangles) 50–60 min after TBS (one-way ANOVA: *F*_2,34_ = 58.45, ****p < 0.0001; Sidak’s *post hoc* multiple comparisons: VEH vs. Y397F, *t*_34_= 7.30, ****p < 0.0001; VEH vs. Y397D, *t*_34_ = 3.83, **p < 0.01; Y397F vs. Y397D, *t*_34_ = 10.58, ****p < 0.0001). (L) Paired-pulse facilitation (PPF) of the synaptic response in CA1 dorsal hippocampus of VEH (gray), Y397F (blue), and Y397D (green) for inter-pulse intervals at 40, 100, and 200 ms (two-way ANOVA: effect of time × group interaction, *F*4,68 = 3.38, **p < 0.05; Sidak’s *post hoc* test: VEH vs. Y397D, 40 ms *t*_18.45_ = 4.38, **p < 0.01; 100 ms *t*_20.35_ = 3.77, **p < 0.01; 200 ms *t*_21.79_ = 3.71, **p < 0.01). Right, representative examples of PPF collected at 40 ms inter-stimulus interval in slices from each group. Traces of the first (black line) and second (red line) fEPSPs are superimposed. Scale bar, 1 mV/5 ms. (M and N) (M) IO curve and (N) slope of IO curves (one-way ANOVA: *F*_2,34_ = 0.37, *p* = 0.70) for VEH, Y397D, and Y397F comparing pre-synaptic fiber volley (mV) to fEPSP slopes (mV/ms) across a range of stimulation currents in CA1b. Data are presented as means ± SEM for *n* = 6 males/condition; 11–14 slices/condition. See also Figures S3 and S4.

We generated CREST tyrosine 397 to phenylalanine (Y397F) and CREST tyrosine 397 to aspartic acid (Y397D) point mutants and examined their effects on LTM and LTP (Figures S3A and S3B). We predicted that the Y397F point mutant would impair LTM formation, whereas the Y397D point mutant would enhance LTM formation. Mice were subjected to the OLM task and received bilateral dCA1 infusion of a vector-free *in vivo* transfection reagent (in vivo-JetPEI) complexed to a plasmid expressing the CREST Y397F point mutant at 24 h after habituation (i.e., day 11 of the OLM procedure), followed by 1 day of recovery before proceeding through the OLM behavior (in vivo-jetPEI reagent without DNA delivered as vehicle control [VEH]) (Figure 4A).^44,45^ Both the VEH and the Y397F in vivo-jetPEI transfection conditions successfully habituated to the OLM context, and we observed no differences in total distance traveled throughout days of habituation (Figure S4A). VEH control (VEH-T10) and the Y397F point mutant experimental conditions (Y397F-T10) received threshold training (10 min) on day 14 of OLM behavior. Infusion of Y397F-T10 had no effect on total exploration or DI for threshold training compared with VEH-T10 controls (Figures 4B and 4C). Both groups exhibited comparable total exploration during the test session (Figure 4D). During the retention test, we observed that the Y397F-T10 group showed a significantly decreased DI compared with the VEH-T10 control group (Student’s *t* test: *t*_28_ = 7.76, ****p < 0.0001) (Figure 4E). We also assessed anxiety behavior via open field and elevated plus maze, where we observed that infusion of the CREST Y397F point mutant (Y397F-T10) did not alter anxiety-like behavior compared with VEH-T10 control (Figures S4B and S4C). These results demonstrate that the Y397F point mutation in CREST significantly impairs LTM formation.

In parallel experiments, a CREST Y397D point mutant was delivered bilaterally into the dCA1 in complex with in vivo-jetPEI 3 days prior to OLM subthreshold training (3 min, which we refer to as T3), which is not sufficient for LTM formation (schematic, Figure 4A). Both the VEH-T3 and the Y397D-T3 transfection conditions successfully habituated to the OLM context, and we observed no difference in total distance (Figure S4D). During the subthreshold training session, both the VEH-T3 control and the Y397D-T3 point-mutant conditions had comparable total exploration and displayed no object preference bias (Figures 4F and 4G). During the retention test, we found that, while both groups had comparable total exploration, VEH-T3 controls were not able to acquire LTM (as predicted); however, the Y397D-T3 condition showed a significant preference for the moved object (Student’s *t* test: *t*_25_ = 10.49, ****p < 0.0001) (Figures 4H and 4I). When we examined VEH-T3 and Y397D-T3 groups in open field and elevated plus maze, we observed no differences (Figures S4E and S4F). These results demonstrate that the Y397D point mutation in CREST can enable LTM formation in subthreshold, insufficient learning conditions.

To further examine the role of CREST in memory formation, we used an adeno-associated virus (AAV) approach to overexpress the Y397F or Y397D point mutant in the adult dCA1 and compared with an empty vector (EV) control in two distinct hippocampal-dependent tasks, with either threshold or subthreshold learning period, to assess LTM (Figure S5). First, mice that received dCA1 infusion of AAV-Y397F, AAV-Y397D, or AAV-EV were subjected to the object-based memory updating task (objects in updated locations [OUL]) developed and described by our group in Kwapis et al.^46^ to measure LTM for original information after memory updating (Figure S6A).^46^ Similar to our transfection approach, animals that received the AAV-Y397F infusion condition and a threshold learning training period were significantly impaired in memory updating relative to AAV-EV control (48 h LTM test; DI, Student’s *t* test: *t*_17_ = 2.25, *p < 0.05; Figures S6B–S6D), whereas the animals with infusion of the AAV-Y397D condition were able to facilitate LTM for updated information when exposed only to an insufficient, subthreshold learning period during training, relative to AAV-EV control (48 h LTM test; DI, Student’s *t* test: *t*_18_ = 2.91, **p < 0.01; Figures S6E–S6G). Second, we examined other aspects of hippocampal-dependent memory via contextual fear conditioning, an inherently more stressful learning condition than OLM or OUL, where the AAV-Y397F and AAV-Y397D point-mutant conditions were exposed to either threshold or subthreshold context acquisition protocols (Figure S7A).^47–49^ While the AAV-Y397F point-mutant condition showed impaired LTM for contextual memory, in contrast to our expected outcome, the AAV-Y397D point-mutant also impaired LTM for contextual fear, relative to AAV-EV control (Y397F, one-tailed Student’s *t* test, *t*_17_ = 2.04, *p < 0.05, and Y397D, one-tailed Student’s *t* test, *t*_18_ = 1.96, *p < 0.05; Figures S7B and S7C). This discrepancy will be addressed in the discussion, but together these results further strengthen the importance of the CREST Y397 residue in bidirectional regulation of hippocampal-dependent LTM formation.

To test the effect of CREST Y397 point mutants on synaptic plasticity, we examined LTP in the dCA1 of slices harvested 4 days after in vivo-jetPEI-mediated transfection of the CREST Y397F or Y397D point mutants or VEH experimental conditions. The time course of TBS-induced LTP in hippocampal slices from Y397F, Y397D, and VEH mice showed comparable short-term potentiation during the first 10 min after induction (Figure 4J). Thereafter, we found that hippocampal expression of the CREST Y397F point mutant interfered with the stabilization of late-phase LTP, as fEPSP responses rapidly decayed toward baseline relative to the VEH control condition (Figure 4J).^50–52^ In contrast, recordings in hippocampal slices with expression of the CREST Y397D point mutant showed an overall enhancement in LTP compared with the VEH control condition (Figure 4J). When we examined mean levels of potentiation at 50–60 min following induction, the Y397D-infused group showed significantly enhanced potentiation, while LTP in slices from the Y397F-infused group was significantly impaired relative to VEH slices (one-way ANOVA: *F*_2,34_ = 58.45, ****p < 0.0001) (Figure 4K). Despite the effects of each point-mutant condition on LTP, only one group showed altered baseline synaptic transmission. PPF and input/out curves were comparable between VEH and Y397F; however, the PPF in slices from the Y397D-infused experimental condition were significantly enhanced over VEH at each stimulation interval (Figures 4L–4N). Together, these results demonstrate that manipulation of CREST Y397 bidirectionally affects LTP.

### Profiling of the LTM consolidation window reveals distinct patterns of gene expression associated with CREST Y397

**Figure 5.**
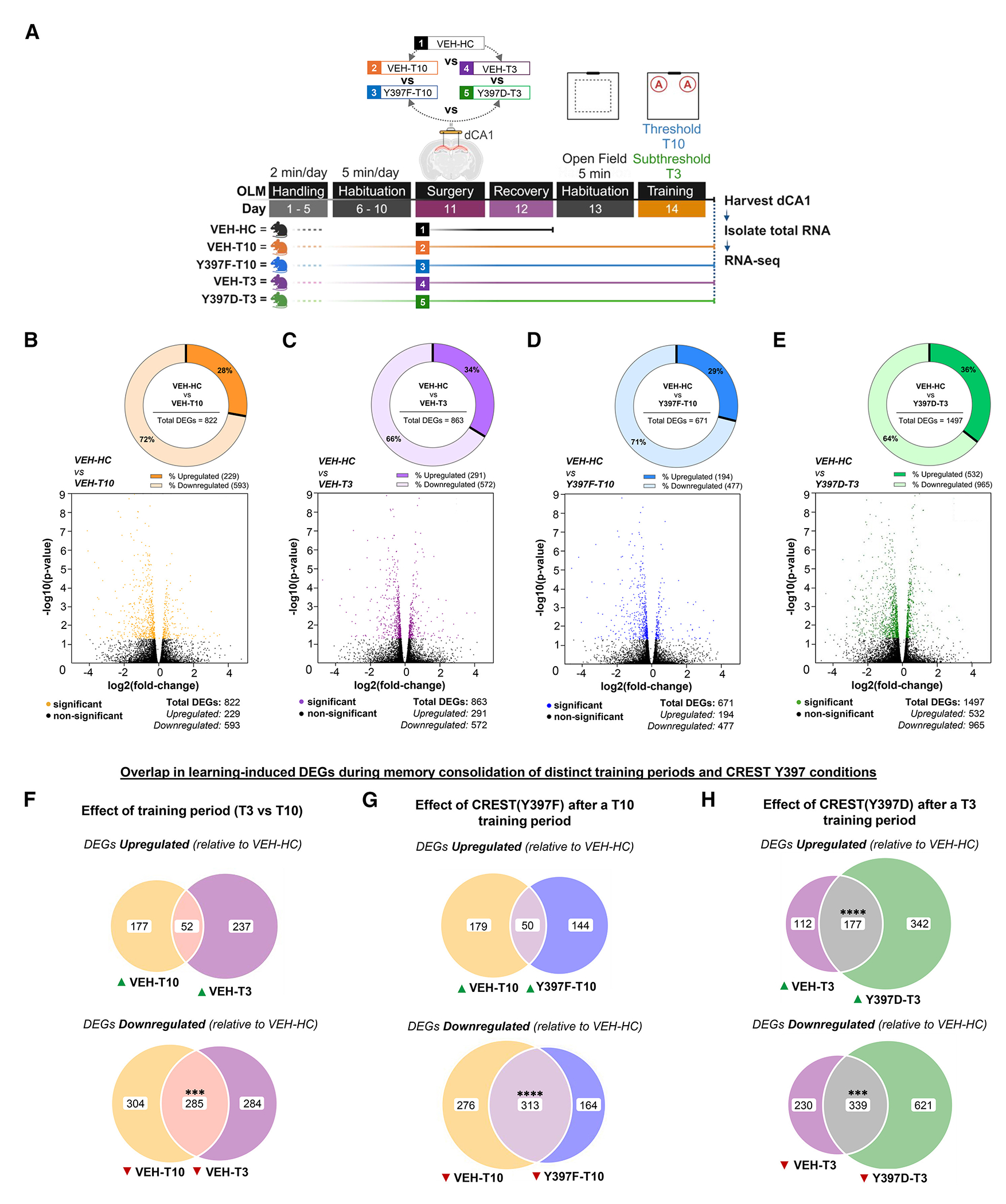
Altering Crest Y397 in the dCA1 has OLM training-period-specific effects on learning-induced transcriptional profiles (A) Timeline of OLM behavior for dCA1 tissue collection 1 h after either a subthreshold (T3) or a threshold (T10) training period (molecular window of memory consolidation) for adult mice that received vehicle control (VEH), CREST Y397F, or CREST Y397D experimental conditions. (B–E) Volcano plots illustrate significance (*y* axis) and magnitude (*x* axis) (*p* < 0.05, color coded; *p* ≥ 0.05, black), and corresponding donut charts summarize percentage of learning-induced DEG (upregulated and downregulated) experimental conditions. (F–H) All Venn diagrams show upregulated or downregulated DEGs (*p* < 0.05, relative to VEH-HC) from the comparison of two conditions, with the numbers of DEGs and Fisher’s exact test (FET) statistic (FDR-adjusted *p* value annotated) (see Tables S5, S7, S10, S12, S15, and S18; Figures S8, S9, S10, S11, S12, S13, S14, and S15).

To investigate how the CREST Y397 point mutations affect learning-induced gene expression, we performed bulk RNA-seq and downstream analyses. We examined gene expression changes 1 h after either a subthreshold (3 min, T3) or a threshold (10 min, T10) training period from dCA1 tissue (Figure 5A, schematic). We compared groups exposed to subthreshold (VEH-T3 and Y397D-T3) and threshold (VEH-T10 and Y397F-T10) training periods to HC controls (VEH-HC) to identify distinct expression profiles that are enhanced (by the CREST Y397D point mutant) or impaired (by the CREST Y397F point mutant) during memory consolidation (Figure S8A). All five experimental conditions have reads alignment rates between 90% and 95%, and each sample has a PHRED base quality score greater than 30, a benchmark for high-quality next-generation sequencing, which is also comparable to our previous RNA-seq studies (Figures S8B and S8C).^28,30,36,53^ The receiver operating characteristic (ROC) curve produced by Cyber-T analysis of this dataset provided an area under the curve (AUC) metric that demonstrates robust model performance (AUC = 0.978) (Figure S8D).^54,55^ Principal-component analysis of log_2_-TMM-normalized counts (*n* = 30 libraries) showed only partial separation of the five experimental groups, with PC1 explaining 41.1% and PC2 10.0% of the total variance (Figure S5E). This modest spread is expected given that the CREST Y397 point mutants alter a limited subset of transcripts, whereas most variance arises from shared hippocampal expression. In this study, we report a comprehensive analysis of this dataset with Cyber-T, but as a comparison, we also provide a brief differential gene expression (DGE) analysis with DESeq2 for each condition relative to HC or training period control, where we found that indeed Cyber-T is a more conservative analysis, which produces already adjusted *p* values through its Bayesian approach (Table S2).^54–56^

The first question we asked was whether there is a difference in gene expression induced following 10 min (threshold) of training vs. 3 min (subthreshold) of training. As these two training periods have different effects on LTM formation, we predicted large differences in learning-induced gene expression profiles. As shown in Figures 5B and 5C, the VEH-T10 and VEH-T3 control groups (compared with an HC control experimental condition, VEH-HC) had comparable numbers of total differentially expressed genes (DEGs), 822 and 863, respectively (Tables S3 and S4). However, VEH-T10 and VEH-T3 shared only 52 upregulated DEGs (Fisher’s exact test [FET]: false discovery rate [FDR]-adjusted *p* = 0.661) (Figure 5F top; Tables S5, S6, and S7; see Figure S9 for Gene Ontology [GO] analysis). In contrast, there were 285 shared DEGs among the downregulated DEGs between VEH-T10 and VEH-T3 (FET: FDR-adjusted ****p < 0.0001) (Figure 5F, bottom). We also examined overlap in the VEH-T10 and VEH-T3 conditions when DEGs have opposite directions of expression but found as little as a 2–3 gene overlap (Figure S1; Tables S8 and S9). Thus, overall, these two different training periods lead to similar overall numbers of DEGs, but the identity of the genes is mostly different, which supports the different effects these training periods have on LTM formation.

We then examined the effect of the CREST Y397F point mutant on learning-induced gene expression following a 10 min threshold training period. The Y397F-T10 point-mutant condition exhibited a total of 671 DEGs, with 194 being upregulated and 477 being downregulated (Figure 5D; Table S10). When we compared the up- and downregulated DEGs to the VEH-T10 controls (because the Y397F group received the T10 training period), we observed an overlap of 50 genes that were upregulated (FET: FDR-adjusted *p* = 0.0626) and 313 genes that were downregulated (FET: FDR-adjusted *p* < 0.0001) between VEH-T10 and Y397F-T10 conditions (Figure 5G; Tables S11 and S12; Figure S11 for GO analysis). A within-T10 training period comparison revealed that expression of the Y397F-T10 point mutant can alter expression of 167 DEGs relative to the corresponding VEH-T10 condition (Figure S12A; Table S13). To understand how the CREST Y397F mutant impairs LTM, we examined which DEGs were upregulated in the VEH-T10 group and were downregulated in the Y397F-T10 group (Figure S12B). We found only two genes, *Atxn7l1* and gene ID 4933409K07RIK (Table S14). Interestingly, both are associated with human diseases.^57,58^

Next, we examined the effect of the CREST Y397D point mutant following a 3 min subthreshold training period. The Y397D-T3 condition had a total of 1,497 DEGs, with 532 being upregulated and 965 being downregulated (Figure 5E; Table S15). When we compared the Y397D-T3 and VEH-T3 conditions, we observed an overlap of 177 genes that were upregulated (FET: FDR-adjusted ****p < 0.0001) and 339 genes that were downregulated (FET: FDR-adjusted *p* < 0.001) (Figure 5H; Tables S16 and S17; Figure S13 for GO analysis). A within-T3 subthreshold training period comparison revealed that expression of the Y397D-T3 point mutant can alter expression of 416 DEGs relative to the corresponding VEH-T3 condition (Figure S14A; Table S18). To understand how the CREST Y397D mutant transforms subthreshold training into LTM for object location, we examined which DEGs were upregulated in both the VEH-T10 group and the Y397D-T3 point-mutant condition. We found an overlap of 96 genes, which consisted of several immediate-early genes (i.e., *Egr1*, *Homer1*, c-*Fos*, and *Dusp6*) known to be involved in LTM formation (Figure S14B; Table S19).^37,59^ In addition, a past study by Qiu and Ghosh^10^ used cultured neurons and identified the *Grin2b* and c*Fos* genes as physical targets of CREST using chromatin immunoprecipitation (ChIP)-qPCR.^23,37–39^ In our dataset, we see a significant upregulation of *Grin2b* (fold change = 1.27, *p* < 0.05) only in the Y397D-T3 condition (Table S15), and *c-Fos* was upregulated in all training period conditions except for the Y397F-T10 pointmutant condition. We also asked the question of whether mutation of CREST Y397 can affect the identity of the DEGs expressed in the T10 and T3 OLM training periods (relative to HC). The Y397F-T10 point-mutant condition recapitulates only 367 of the 822 total DEGs in the control T10 training period (VEH-T10) (Figure S15A; Table S20). Alternatively, the Y397D-T3 point-mutant condition expressed 497 of the 822 total DEGs of the controls with the T10 training period (VEH-T10) and only 517 of the 863 total DEGs in the control condition with a subthreshold training period (VEH-T3), relative to HC (Figures S15A and S15B; Table S20). Sorting by the VEH-HC, VEH-T10, or VEH-T3 condition did not reveal any distinct gene expression patterns across all conditions (Figures S15C and S15D). However, a comparison of the 497 and 517 DEGs that the Y397D-T3 condition recapitulates within the T10 and T3 training periods, respectively, revealed 279 DEGs that are shared (regardless of direction of expression), which further demonstrates the ability of the Y397D point mutation to transform subthreshold learning periods (Figures S15F and S15G; Table S21).

**Figure 6.**
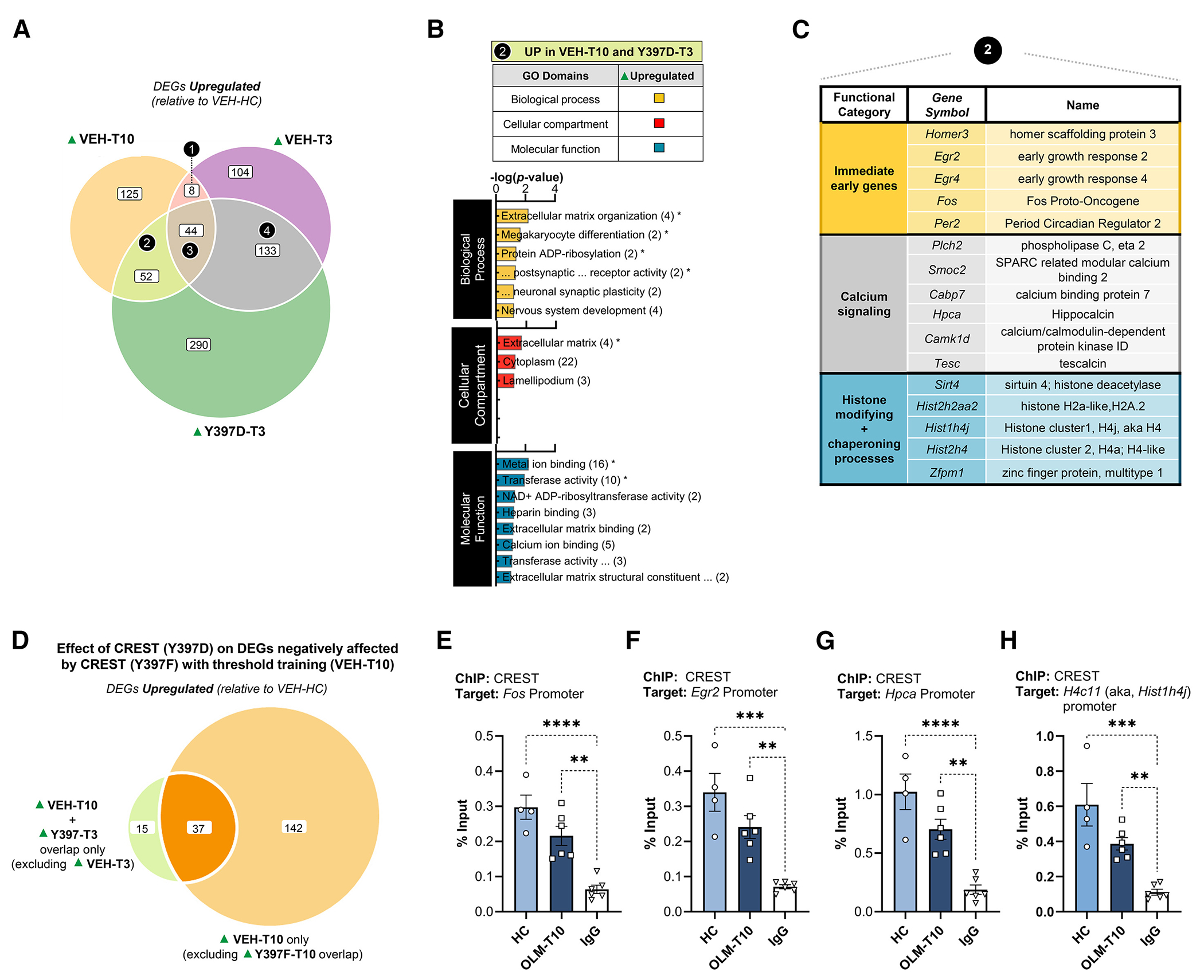
Upregulated DEGs that normally induce in threshold T10, fail to induce in subthreshold T3, but are captured by the CREST Y397 point mutant Y397D (A) A three-way Venn diagram comparing upregulated learning-induced genes from differential gene expression analysis (*p* < 0.05 relative to VEH-HC, homecage) for VEH with OLM threshold (VEH-T10, orange), VEH with OLM subthreshold training period (VEH-T3, purple), and CREST Y397D point mutant with subthreshold training period (Y397D-T3, green), with overlaps #1–#4 (black icons) and number of DEGs (white boxes) (Table S22). (B) Gene Ontology (GO) analysis showing the top 10 terms for the biological process, cellular compartment, and molecular function GO domains (ranked by – log(*p*), *y* axis) for overlap #2 for the DEGs (*p* < 0.05, *n* = 52) that are upregulated only in both VEH-T10 and Y397D-T3 (Table S23). The number of DEGs from overlap #2 is annotated in parentheses per GO term (asterisks indicate *p* < 0.05). (C) Summary of DEGs in overlap #2 associated with histone modifying/chaperone processes, immediate-early genes, and calcium signaling. (D) Venn diagram compares the 179 DEGs negatively affected by the Y397F-T10 experimental condition (relative to VEH-T10, Figure 5G, top) with the 52 DEGs where the Y397D-T3 and VEH-T10 experimental conditions overlap (relative to VEH-T3, A, overlap #2) (Table S24). (E–H) Results of chromatin immunoprecipitation (ChIP) in dCA1 tissue collected under HC or 1 h after OLM threshold (T10) conditions (see Figure 3A diagram, *n* = 4–6/condition, all males) and examined for CREST occupancy at the promoter of *Fos* (one-way ANOVA: *F*_2,13_ = 23.21, *p* < 0.0001; Sidak’s *post hoc* multiple comparisons: HC vs. IgG, *t*_13_ = 6.50, ****p < 0.0001; OLM-T10 vs. IgG, *t*_13_ = 4.74, **p < 0.01), *Egr2* (one-way ANOVA: *F*_2,13_ = 18.10, ***p < 0.001; Sidak’s *post hoc* multiple comparisons: HC vs. IgG, *t*_13_ = 5.78, ***p < 0.001; OLM-T10 vs. IgG, *t*_13_ = 4.08, **p < 0.01), *Hpca* (one-way ANOVA: *F*_2,13_ = 21.58, *p* < 0.0001; Sidak’s *post hoc* multiple comparisons: HC vs. IgG, *t*_13_ = 6.34, ****p < 0.0001; OLM-T10 vs. IgG, *t*_13_ = 4.37, **p < 0.01), and *H4c11* (aka *Hist1h4j*) (one-way ANOVA: *F*_2,13_ = 18.07, ***p < 0.001; Sidak’s *post hoc* multiple comparisons: HC vs. IgG, *t*_13_ = 5.91, ***p < 0.001; OLM-T10 vs. IgG, *t*_13_ = 3.65, **p < 0.01). Data are presented as means ± SEM.

To further explore the intersection of learning periods and the CREST Y397D point mutant, we examined a three-way overlap from the RNA-seq data. Figure 6A shows a three-way Venn diagram between the VEH-T10, VEH-T3, and Y397D-T3 groups. This allowed us to determine the genes that are induced with threshold learning (VEH-T10), fail to be induced during subthreshold training (VEH-T3), but are induced by the Y397D point mutant with a subthreshold training period (Y397D-T3). We identified 52 DEGs that are induced by both threshold training (VEH-T10) and the Y397D point mutant following subthreshold training (Y297D-T3) (Figure 6A; Table S22). GO analysis of these 52 genes revealed annotation terms associated with several key molecular and cellular mechanisms relevant to synaptic plasticity and memory formation (Figure 6B; Table S23). For example, Figure 6C shows a summary of several genes associated with calcium signaling (represented by *Capb7*, *Plch2*, *Smoc2*, *Hpca*, *Camk1d*, and *Tesc*), known immediate-early genes (represented by *Homer3*, *Egr2*, *Egr4*, *Per2*, and *c-Fos*), and histone isoforms and histone chaperone complexes (represented by *Sirt4*, *Hist2h2aa2*, *Hist1h4j*, *Hist2h4*, and *Zfpm1*). The histone category may have been excluded from the GO analysis because these genes are not currently classified within the current DAVID Knowledgebase. We further compared these 52 DEGs to the 179 DEGs that the Y397F-T10 failed to upregulate compared with learning-induced DEGs in the VEH-T10 control condition. This comparison revealed that, among the 52 DEGs that the Y397D-T3 point-mutant condition can facilitate to resemble DEGs from threshold learning (T10), 37 (including Egr2, Histh4j, Homer3, and Hpca, to name a few) overlapped with the 179 DEGs affected by expression of the Y397F point mutant in a T10 period (Figure 6D; Table S24). To determine if CREST is directly regulating these DEGs, we performed ChIP-qPCR to measure changes in CREST occupancy after an OLM T10 period at the promoter region of c-*Fos*, *Egr2*, *Hpca*, and *H4c11* (aka *Hist1h4j*) (Figures 6E–6H). While CREST binding was highest in the HC condition, we observed only a trend for a learning-induced decrease in CREST occupancy for c-*Fos* (*p* = 0.099), *Egr2* (*p* = 0.12), *Hpca* (*p* = 0.086), and *H4c11* (*p* = 0.060) (Figures 6E–6H). Together, these findings support our underlying hypothesis that the Y397 residue may have a role in facilitating a specific gene expression signature during memory consolidation that supports LTM formation.

### CBP is necessary for Crest Y397D to facilitate memory formation

**Figure 7.**
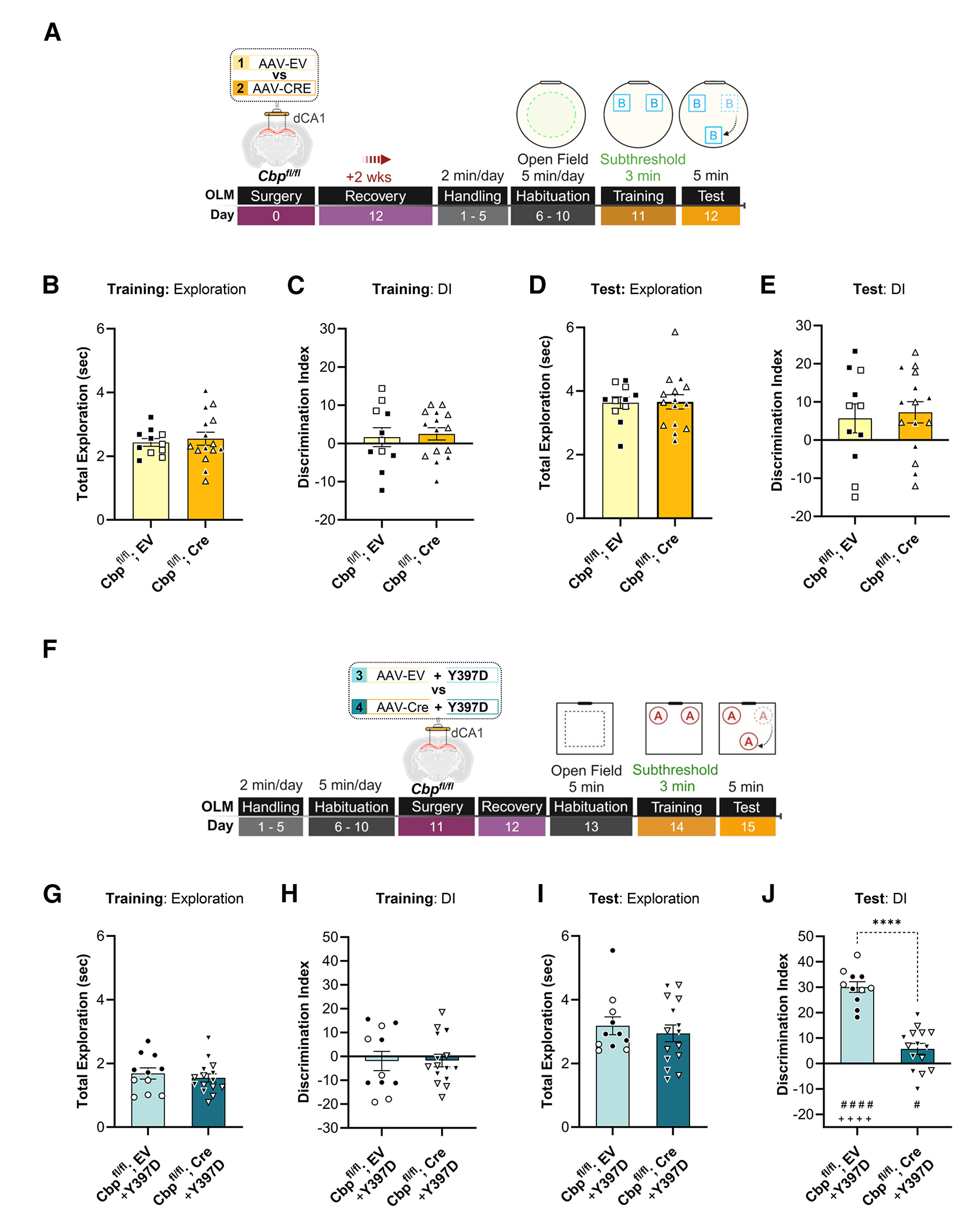
CREST Y397D-mediated facilitation of long-term memory formation is CBP dependent (A) Timeline of OLM behavior in context B (B–E), with *Cbp*^*fl/fl*^ adult mice infused with an AAV expressing either empty vector (1; EV, squares) control or Cre-recombinase (2; Cre, triangles) into the dCA1. (B) Total object exploration during context B OLM subthreshold training (Student’s *t* test: *t*_24_ = 0.46, *p* = 0.65). (C) DI during context B OLM subthreshold training session (Student’s *t* test: *t*_24_ = 0.30, *p* = 0.77). (D) Total exploration of objects during context B OLM test session (Student’s *t* test: *t*_24_ = 0.09, *p* = 0.93). (E) DI during context B OLM test session (Student’s *t* test: *t*_24_ = 0.35, *p* = 0.73). (F) Timeline of OLM behavior in context A (G–J), where EV- and Cre-infused *Cbp*^*fl/fl*^ groups from context B received bilateral jetPEI-meditated transfection of CREST Y397D point mutant: 3, *Cbp*^*fl/fl*^; EV + Y397D (circles) and 4, *Cbp*^*fl/fl*^; Cre +Y397D (downward triangles). (G) Total object exploration during context A OLM subthreshold training (Student’s *t* test: *t*_24_ = 0.65, *p* = 0.52). (H) DI during context A OLM subthreshold session (Student’s *t* test: *t*_24_ = 0.043, *p* = 0.97). (I) Total object exploration during context A OLM test session (Student’s *t* test: *t*_24_ = 0.61, *p* = 0.55). (J) DI for context A OLM test session (Student’s *t* test: *t*_24_ = 7.98, ****p < 0.0001) with pre-planned comparisons annotated (Student’s *t* test: *Cbp*^*fl/fl*^; EV + Y397D [pre-planned paired *t* test, training × test: *t*_20_ = 7.05, ^####^*p* < 0.0001]; *Cbp*^*fl/fl*^; Cre +Y397D [pre-planned, paired *t* test, training × test: *t*_28_ = 2.23, ^#^*p* < 0.05]; and context B vs. A [pre-planned, paired *t* test: EV *t*_20_ = 5.621, ^++++^*p* < 0.0001, and Cre *t*_28_ = 0.43, *p* = 0.67). *n* = 11–15 (5–8 males and females)/group. Data are presented as means ± SEM and represented as combinations of two independent experiments. Males, white squares, triangles, and circles. Females, black squares, triangles, and circles. See also Figure S16.

The Y397 residue is in the small CBP-binding domain of CREST, which suggests that CBP may modulate CREST-dependent effects on LTM. To test this possibility, we expressed the CREST Y397D point mutant in *Cbp^fl/fl^* mice. We first generated a focal deletion of CBP (Figure S16A) in the dCA1, as in our previous study, using AAV-Cre.^27^ Male and female *Cbp^fl/fl^* mice received dCA1 infusion of either AAV-EV (i.e., empty vector, *Cbp^fl/fl^*) or AAV-Cre recombinase (*Cbp^fl/fl^*; Cre) 2 week prior to the start of OLM behavior in a circular chamber with object set B (see experimental timeline in Figure 7A). Both received subthreshold (T3) training (Figures 7B and 7C) to ensure that focal deletion of CBP had no effect on LTM formation, which is important as the subsequent experiment examined the effect of CREST Y397D on transforming subthreshold learning into LTM in a CBP-dependent manner. Throughout habituation and during subthreshold training sessions, animals did not differ in total exploration of objects or DI (Figures 7B and 7C). As predicted, during OLM test sessions AAV-EV- and AAV-Cre-infused *Cbp^fl/fl^* groups had comparable object exploration and DI when provided subthreshold training (Figures 7D and 7E).

After being provided 1 week in HC conditions to allow for a return to behavioral baseline, animals proceeded through a second round of OLM testing in a distinct context and object setting.^28,60,61^ In this second round of OLM testing, both AAV-EV and AAV-Cre groups received bilateral infusion of an in vivo-jetPEI + CREST Y397D transfection complex into the dCA1 24 h after habituation (day 11) (Figure 7F). Following recovery, animals received a subthreshold training period and 24 h retention testing. During habituation and subthreshold training, comparison of *Cbp^fl/fl^*; EV + Y397D and *Cbp^fl/fl^*; Cre + Y397D mice revealed no significant differences in exploration or DIs (Figures 7G and 7H). However, in the retention test session, only *Cbp^fl/fl^*; EV + Y397D showed a significant enhancement in DI relative to *Cbp^fl/fl^*; Cre + Y397D (Student’s *t* test: *t*_24_ = 7.98, ****p < 0.0001) and respective training DI (Student’s *t* test; preplanned paired *t* test, training × test: *t*_20_ = 7.05, ^####^*p* < 0.0001) (Figures 7I, 7J, and S16B–S16E). This effect is conserved in both males and females (no effect of sex × AAV condition interaction, two-way ANOVA: *F*_1,22_ = 0.64, *p* = 0.43) (Figure S16F). In dCA1 tissue isolated 1 h after the end of the OLM test in context A, we detected a significant decrease in *Cbp* expression only in the AAV-Cre infusion condition (Figure S16G) (Student’s *t* test: *t*_12_ = 5.22, ***p < 0.001). Together, these results demonstrate that CBP is necessary for the CREST Y397D point mutant to enable LTM formation following subthreshold learning.

## DISCUSSION

The ability to interact with a nucleosome remodeling enzyme (BRG1) and a HAT (CBP) suggested that CREST may have a central role in epigenetic gene regulation required for long-lasting forms of synaptic plasticity and LTM formation. However, the role of CREST in cognitive function remained unknown. Our initial experiments in this study demonstrated that *Crest* expression (mRNA or protein) does not change during the critical period of consolidation following a learning experience, suggesting that a potential post-translational modification mechanism regulates CREST activity. *In silico* modeling identified a putative phosphorylation site at residue Y397, which is conserved from mouse to human. Although we were unable to verify this phosphorylation using conventional immunoprecipitation-western blot approaches, due to poor available antibodies, or through mass spectrometry, we generated the Y397F and Y397D point mutants to examine their effects on LTP, LTM, and learning-induced gene expression. We found that the Y397D point mutant enhances LTP and enables LTM formation following subthreshold learning, whereas the Y397F point mutant impairs both LTP and LTM. In parallel analysis using the OUL task, we were able to replicate this bidirectional effect of the Y397 point mutants for memory updating with dCA1 AAV overexpression. Contextual fear condition with the AAV approach may have also revealed the detrimental effect of Y397D overexpression, because the condition resulted in a further decrease in freezing below subthreshold control. This can also be further informed by our observation that the expression of the point mutant with the transfection approach resulted in the highest number of DEGs. The Y397 residue is located within the last 9 amino acids of the C terminus, which has been shown to be the minimal binding domain for CBP recruitment.^9^ These results suggest that CREST is regulating learning-induced gene expression via a CBP-dependent manner, and we speculate that the Y397 residue may be a *bona fide* phosphorylation site essential to CBP recruitment, which will be the focus of future investigation.

CBP is a potent HAT that we and others have shown to be necessary for LTP and LTM.^15–17,26,27,32,52,62^ CBP is most well known for its inducible interaction with the transcription factor CREB via the phosphorylation of serine 133 on CREB. Phosphorylation of S133 on CREB is critical for CREB:CBP-mediated gene expression required for LTM formation.^12–14,63^ With regard to CREST, a cell culture study demonstrated that CREST and CBP are physically located (using ChIP-qPCR) on the promoters of *c-Fos* and *Grin2b*.^10^ This raises the possibility that CBP is required for CREST-dependent regulation of memory formation. Indeed, we found that the CREST Y397D point mutant could enable the transformation of subthreshold learning into LTM only in a CBP-dependent manner. Alternatively, the effects we observed with the CREST Y397F point mutant and anti-Crest morpholino on impairing LTM and LTP may point to the availability of CREST or the accessibility to Y397 as rate-limiting factors important for the interaction of CBP and CREST. This result suggests that, similar to CREB, CREST requires CBP for LTM formation. A key open question is whether CREST and CREB interact with CBP simultaneously, independently of each other, or in temporally distinct phases of memory consolidation.

One of the first studies to try to understand how different epigenetic mechanisms are recruited during transcriptional regulation dynamics comes from the yeast field. For example, budding yeast implement a multi-stage, combinatorial coordination of recruiting a nucleosome remodeling complex (SWI/SNF) at gene promoter regions prior to a HAT (SAGA GCN5 adaptor complex) to regulate cell-cycle processes.^64–66^ While insight from the yeast field provides a specific scenario for epigenetic machinery recruitment, CREST in the mammalian brain provides a molecular bridge that may be able to facilitate similar coordinated regulation, by engaging both BRG1 and CBP.^10^ The neuron-specific subunits (CREST, BAF53b, and BAF45b/c) are incorporated into the neuronal BAF nucleosome remodeling (nBAF) complex as neuronal precursor cells differentiate into neurons, and they are indeed essential for that differentiation process.^22,67–69^ We have previously demonstrated that BAF53b is an important subunit of the nBAF complex that is necessary for LTP and LTM.^29–31^ Mutations in most genes whose protein products are part of the nBAF complex have been shown to be associated with human developmental and cognitive disorders. For example, *de novo* mutations in nBAF complex subunit genes are linked to the intellectual disability disorders Coffin-Siris (*BAF250A/B* and *BRG1*), Nicolaides-Baraitser syndrome (*BAF250B*, *BRM*, and *BAF47*), and autism spectrum disorder (*BAF53b*).^70–73^ In humans, more than one-third of cases of ALS show evidence of cognitive impairment, and *de novo* mutations of *CREST* at Ileu123Met and Gln388Stop have been identified in cases.^18–20,74,75^ The localization of these mutations to the BRG1- and CBP-binding domains, respectively, may have a causal link to the disease phenotype. In this study, we demonstrated that CREST has a role in LTP and LTM in the adult mouse brain that is independent of any potential role in development, so our findings provide valuable insight for how we can interpret the significance of a loss of the CBP-binding domain in the Gln388Stop ALS mutation and the possibilities for further exploring the potential of targeted activation of the Y397 site to rescue memory decline with aging.

To begin to understand the role of CREST in regulating learning-induced gene expression that is required for LTM formation, we examined the effects of the CREST mutants on gene expression during memory consolidation. The main comparisons we focused on were how threshold training (10 min) compared with subthreshold training (3 min). A major advantage of the study design for the RNA-seq analysis was the ability to directly compare gene expression during memory consolidation following periods of threshold or subthreshold training. Notably, we observed that, although the 3 min subthreshold and the 10 min threshold training periods induce very similar numbers of DEGs, they share very little overlap gene identity. This may point to more complex aspects of memory consolidation where a longer duration of incidental learning may not necessarily result in a greater number of genes or a proportional accumulation.

To understand how the CREST Y397D point mutant may be facilitating subthreshold training-induced gene expression for LTM, we focused on how it recapitulates the threshold training condition and deviates from the subthreshold training period condition. This specifically revealed gene sets encoding known immediate-early genes, collagen isoforms of the extracellular matrix (ECM) that support synaptic plasticity, several components of the calcium signaling pathway (including the calcium-sensor, *Hpca*), and histone exchange mechanisms.^76–79^ Notably, matrix metalloproteinases (MMPs) that regulate turnover of components of the ECM, including collagen, are elevated with ALS pathology.^80–82^ We detected *Hpca*, a calcium sensor, within our ‘‘calcium ion binding’’ term in the GO analysis, and its role in preventing excitotoxicity in hippocampal neurons expands on the calcium-responsive properties previously described in cell-culture CREST studies.^9,79^

The process of histone exchange and turnover provides another layer of epigenetic regulation whereby nucleosome remodeling complexes, such as SWI/SNF and nBAF, can further affect nucleosome composition to change chromatin structure.^83–88^ Specifically, the turnover of the histone variants H3.3 and H2A.Z is dynamically involved in regulating transcriptional activation, synaptic plasticity, and LTM formation in adult dCA1 neurons both positively and negatively, respectively.^89–92^ In our targeted differential gene expression analysis of DEGs that are enabled by the Y397D-T3 and recapitulate a subset of the VEH-T10 learning-induced genes, we identified a significant increase in expression of genes that encode histone isoforms (i.e., *Hist1h4j, Hist2h2aa2*, and *Hist2h4*).^93^ While these specific histone isoforms have not been previously implicated in memory function in the adult dCA1, a *de novo* heterozygous missense mutation in a lysine residue of HIST1H4J has been linked to intellectual disability disorder and further highlights non-redundant and lack of compensatory function by other histone isoforms.^94^ Furthermore, a role for CREST Y397 regulating nucleosome composition is further supported by the upregulation of *Zfpm1* (i.e., zinc-finger protein, FOG1), which directly interacts with RBBP4 (a chaperone of H3/H4 tetramers) within the chromatin assembly factor-1 (CAF-1) and the HDAC complex NuRD.^95–97^ The bidirectional function of Y397 was further strengthened when we found these key gene signatures transformed by the Y397D point mutant to also be represented among those DEGs that failed to induce in the Y397F point mutant with threshold learning. This provided us with a subset of 37 DEGs that are oppositely regulated by Y397. We also provide evidence that CREST is in fact binding a subset of these genes (e.g., c-*Fos, Egr2, Hpca*, and *H4c11* [aka *Hist1h4j*]). While CREST occupancy at these Y397-regulated DEGs did not change with learning, it is possible that the 1 h post-threshold training time point we used may have missed the more dynamic activity of CREST binding, considering that other transcription factors, such as CREB, can be rapidly phosphorylated within the first 30 min following contextual fear acquisition.^38^ The Y397-regulated DEGs were represented in a recent RNA-seq dataset by Chatterjee et al., who used the CBP^KIX/KIX^ transgenic mouse to uncouple interactions with CREB, where we see *c-Fos, Egr2*, and *Per2*.^26^ This would suggest that at least a subset of the Y397-regulated DEGs are directly regulated via a CREST-CBP interaction. Thus, CREST may have a role in regulating the expression of genes that encode components of mechanisms that affect nucleosome composition in addition to calcium signaling response and synapse function.

In summary, this study demonstrates a role for CREST in synaptic plasticity, memory formation, and the regulation of distinct gene signatures, which further adds to what little we know about it to date. More specifically, the Y397 residue of CREST appears particularly important, as manipulation of this residue has bidirectional effects on LTP and LTM and it may be a putative phosphorylation site. The location of Y397 in the CBP-binding domain of CREST further suggests that CREST functions in a CBP-dependent manner to modulate memory formation, which our data support. This raises intriguing questions regarding the interplay between CREB, CREST, and CBP especially considering the predominant role of CREB/CBP in the learning and memory literature.

### Limitations of the study

The detection of the phosphorylation of Y397 was not possible, and additional time points or further exploration of mass spectrometry methodology is needed. Thus, we can only speculate that Y397 is a phosphorylation site, so we refer to Y397F and Y397D only as point mutants rather than phospho-mutants. While CREST has been previously shown to recruit CBP, further investigation would be necessary to provide sufficient evidence for a CBP mechanism involving Y397 phosphorylation status and CREST-CBP interaction. The scope of this study was limited to a brief glimpse into how CBP is necessary for mediating the ability of the Y397D point mutant to facilitate memory formation, and we cannot make any claims as to when or whether a CREST-CBP interaction is in direct competition with the well-established and essential transcriptional activation that is mediated through CREB-CBP interaction. This study primarily focused on the CREST-CBP interaction independent of the status of the nBAF complex, so it is unclear whether the effects of the Y397 residue involve CREST that is also interacting with BRG1. This study did not test the effects of overexpressing wild-type CREST. As such, we cannot distinguish between the effects of exogenous expression and those of the point mutations.

## STAR★METHODS

### RESOURCE AVAILABILITY

#### Lead contact

Requests for further information, resources, and reagents should be directed to and will be fulfilled by the lead contact, Dr. Marcelo A. Wood (mwood@uci.edu). Supplemental information and source data are available for this article.

#### Materials availability

Plasmids generated in this study are deposited at the University of California, Irvine, Center for Neural Circuit Mapping (CNCM) Viral Vector Core Facility (RRID: SCR_025573). All unique/stable reagents generated in this study are available from the lead contact with a completed material transfer agreement.

### EXPERIMENTAL MODEL AND STUDY PARTICIPANT DETAILS

#### Mice

Young adult mice were between 2 and 4 months old at the time of testing. All mice were C57BL/6J (Jackson Laboratory, RRID:IMSR_JAX:000664). The previously characterized CBP conditional knockout mice (*Cbp^fl/fl^*,genotyping primers: forward 5’- CTGCTCTACCTAAATTCCCAG-3’ and reverse 5’- GGGGAAATTTTGGCTGGCAAG-3’) (RRID:IMSR_JAX:025178) are maintained on a C57BL/6 background and received Cre recombinase infusion to produce focal CBP deletion.^27,100^ Mice had access to food and water *ad libitum*, and lights were maintained on a 12h light/dark cycle. All behavioral testing was performed during the daytime light cycle between the hours of 8am and noon to account for memory performance oscillations.^101^ Animals were only single-housed prior to the start of behavioral experiments. All experiments were conducted according to US National Institutes of Health guidelines for animal care and use and were approved by the Institutional Animal Care and Use Committee of the University of California, Irvine.

#### Sex as a biological variable

Both male and female vertebrate animals were included as a factor in the experimental design, analyses, and reporting in this study where possible to account for the effect of sex as a biological variable (SABV). This study factored the new SABV National Institute of Health (NIH) initiative to enhance reproducibility, rigor, and transparency.

### METHOD DETAILS

#### Surgery

Mere were anesthetized with isoflurane (induced, 4%; maintained 2%) and mounted on a stereotax (Stoelting, #51730D). Custom injectors (Plastics One) were lowered at a rate of 0.2mm/15sec for bilateral infusion into the dorsal hippocampus (dCA1): AP= −2.0mm, ML= ±1.5mm, DV= −1.5mm; relative to Bregma. Once at DV coordinate, the injector was left in place for 2min prior to beginning infusion.^25,27,28^ Our conditions involved infusing either 1μL of a vivo-morpholino oligo (Control or anti-*Crest*, Gene Tools), AAV-CMV-EV (UC Viral Core), AAV-CMV-Cre (Addgene), AAV1-miniCaMK2-hBG-EV, AAV1-miniCaMK2-hBG-HA-CREST-Y397F or AAV1-miniCaMK2-hBG-HA-CREST-Y397D (UCI Viral Core) per dCA1 hemisphere at a rate of 12μL/hr. For the dCA1 infusion of the in vivo-jetPEI/DNA complexes (Polyplus), each animal received 1.5μL per hemisphere at a rate of 12μL/hr. After complete infusion, the injectors remained in place for an additional 5mins to allow for optimal diffusion before raising by 0.1mm from target DV coordinate for a 1min hold and proceeding to withdrawing at a rate of 0.1mm/15secs. Mice were allowed to recover after surgery before proceeding to behavior testing and closely monitored for distress. AAV infusions were performed 3 weeks prior to behavior testing. Vivo-morpholino and in vivo-jetPEI/DNA complexes were infused 4 days prior to behavior testing.

#### Morpholino, site-directed mutagenesis, *in vivo* transfection and AAV

For vivo-morpholino (VM) oligo infusions, the standard negative control oligo (‘Control’; 5’ -CCTCTTACCTCAGTTACAATTTATA-3’, #PCO-VivoStandardControl-100) and a custom oligo targeting the 5’-UTR of the mouse *Crest* mRNA sequence (‘anti-Crest’; 5’-ACTCAGCCCGTGATCATTGGG-3’) were diluted in sterile water to a final working concentration of 5μM. For generating CREST point-mutants, a plasmid encoding the mouse *Crest* with a N-terminus HA-tag under the regulation of the miniCaMK2 promoter (Viral Core, UCI Center for Neural Circuit Mapping) was used with the GeneArt Site-Directed Mutagenesis system (ThermoFisher Scientific, #A13282) and NEBuilder HiFi DNA Assembly Master mix (NEB, #E2621S). The following primers were used to generate the point mutants: CREST-Y397F (forward: 5’- GGGCCAGTTTGGAAATTACCAGCAATAAAAGCTT-3’, reverse: 5’- GGGCCAGTTTGGAAATTACCAGCAATAAAAGCTT-3’) and CREST-Y397D (forward: 5’- GGGCCAGGATGGAAATTACCAGCAATAAAAGCTT-3’; reverse: 5’- TTTCCATCCTGGCCCTGTTCATAGCC-3’) (Figures S3A and S3B). *In vivo* transfection was carried out with the in vivo-jetPEI reagent kit (Polyplus, #101000040) prepared and optimized according to the manufacturer’s and previously established protocol recommends.^44,45^ Briefly, 20μg of DNA plasmid (endotoxin free and suspended in sterile water) encoding a CREST Y397 point-mutant was diluted in 10% sterile glucose and 3.2μL of in vivo-jetPEI polymer reagent (i.e., 0.16μL/μg of DNA, N/P=8) to prepare a 60μL injection master mix in a final 5% glucose that was incubated for 15min at RT to form JetPEI/Plasmid complexes prior to dCA1 infusion. The AAV1-CMV-Cre (titer: ≥1x10^13^ vg/mL, Addgene viral prep # 105537-AAV1, was a gift from Dr. James M. Wilson). AAV1-miniCaMK2-hBG-EV (empty vector control, titer: 8.88x10^13^ gc/mL), AAV1-miniCaMK2-hBG-HA-CREST-Y397F (1.72x10^13^ gc/mL), AAV1-miniCaMK2-hBG-HA-CREST-Y397D (2.24x10^13^ gc/mL) and AAV-CMV-EV (≥1x10^13^ vg/mL) were made at the University of California, Irvine Center by the Neural Circuit Mapping Viral Production Facility.

#### Open field and object location memory task

The function of dCA1 is particularly essential for processes of long-term memory formation, including encoding, consolidation and retrieval.^24,25,27,29,30^ For examining hippocampal-dependent memory formation, we used the well characterized object location memory (OLM) task.^24,28,60,61,102–104^ Briefly, mice received 5 days of handling (2mins/day) followed by habituation to the OLM chamber context (32cm (l) x 32cm (w) x 31cm(h)) for 5 consecutive days (5mins/day). Either on the 5^th^ or on a 6^th^ day (following recovery from surgical procedures) of habituation zones defined as edges and center of the OLM context field were designated in the ANY-maze software (Stoelting, #6000) and used to track time in zone and reported as percentage of time. The day after the end of habituation mice received either a threshold (10min) or sub-threshold (3min) training session that consisted of free access to explore two identical objects (100mL Pyrex beakers weighted with quickset cement, ThermoFisher Scientific, Fisher: cat# 02-540H). During the retention testing session for long-term memory formation (24hrs after training session), the mice were given access to explore the OLM chamber context for 5mins with one of the identical objects displaced from the training position to a distal novel location. For all OLM task sessions, the ANY-maze tracking software 4.99 (Stoelting Co.) was used to collect videos and tracking data. A multi-timer was used to score exploration of each object during the session based on defined criteria described in Vogel-Ciernia et al.^60^ In addition to reporting total exploration (*t*) and distance travelled, we also report exploration preference for objects in a familiar or novel location based on video scoring to obtain a discrimination index (DI = (*t*_novel_ – *t*_familiar_) / (*t*_novel_ + *t*_familiar_) x 100%). Based on training session, mice that showed a preference for one object (DI > ±20) were removed from the study. A DI of 0 indicates equal preference for the two objects. A DI higher than 25 in the 5min retention test session is considered as a significant preference for the moved object and successful acquisition of long-term memory formation for object location. A second OLM context (defined as ‘Context B’) consisted of a modified 5-gallon round bucket (24.5cm (h) x 27.5cm (d)), weighted square glass candle holders (7.6cm x 7.6cm x 7.6cm), floor bedding distinct to Context A and a peppermint oil (0.1%) scent. When the same mice received multiple behavior task testing, they were proved 7 days of recovery in the colony facility (with refreshed housing material and bedding) immediately following the end of OLM Context B testing and prior to starting OLM Context A testing based on previously established behavioral protocol recommendations.^30,60^ All experimental condition assigned to animals are kept blind throughout behavior for experimenters as well as during scoring of exploration preferences.

#### Elevated plus maze

At 24hrs after the OLM test session, mice were examined for retention of anxiety-like behavior on the plus maze.^28,34,36^ The apparatus consists of two open arms (30 × 5 cm), two enclosed arms (30 × 5 × 15 cm), and a center platform (5 × 5 cm) at the intersection and an overall height of 40cm above the floor. The mice receive a 5min session (starting in the center platform) 24hrs after the end of the OLM test session with ANY-maze recording and data acquisition of time used to calculate percentage of time spent in enclosed versus open arms (i.e., zone). The apparatus was cleaned with 70% ethanol between each mouse.

#### Contextual fear conditioning (CFC)

For contextual fear conditioning, 1 wk after the end of OUL 48 hr LTM test, mice received a single 3 min acquisition session with 2 min and 28 secs of context exposure prior to a 2 sec foot-shock with an intensity for either a threshold (0.70 mA), which produces robust long-term memory formation, or a subthreshold (0.35mA) training protocol.^27,30,36,48^ Animals remained in the context for 30 secs post-shock before being removed from the conditioning chambers. The following day, animals were re-exposed to the original context for a 6 min test session (without a foot-shock) where freezing behavior provided an index for fear based on prior memory formation for the context-shock association.^49,105^ Video Freeze software (version 3.0.0.0, MedAssociates) was used to automatically collect and score animal freezing.

#### Objects in updated locations (OUL) task

The OUL task was carried out as previously described in Kwapis et al.^46^ Briefly, mice were provided with 5 days of handling (2min/day) and 6 days of habituation in the white open field OUL context chambers (60.96 x 45.72 x 26.67-cm). After habituation, animals were trained with two identical objects (C1 and C2) for a single threshold (10min) or subthreshold (3min) session in the habituation context. The following day, mice received a 10min or 3min 24hr LTM test (Update session) during which object C2 is replace by C3 in the new location for memory updating. Finally, mice were given a 48hr LTM test (Update test) where objects C1-3 are reintroduced along with object C4 in a novel location. Memory for the original information is calculated by with the exploration for object A4 and A1 with the following formula: (A4−A1)/(A4+A1) * 100). The ANY-maze tracking software 4.99 (Stoelting Co.) was used to collect videos and tracking data and a multi-timer was used to score videos to log total exploration of each object.

#### Immunohistochemistry

Mice were administered an intraperitoneal standard dose of 50 mg/kg of sodium pentobarbital (stock concentration = 60 mg/ml, Sigma Aldrich, #P3761) for transcardiac perfusion with saline followed by 4% paraformaldehyde (PFA) prior to harvesting whole brain for post-fixation (24hrs) and cryopreservation in 30% sucrose (48hrs). Then, brains were flash frozen and sliced at 40μm on a cryostat (Epredia HM525 NX cryostat, Fisher Scientific #95-664-0EC70) and placed into PBS for subsequent free-floating immunohistochemistry previously described in Harris et.^106^ Briefly, brain sections were wash with PBS (4 × 10 min), permeabilized with 0.5% Triton X-100 in PBS (1 h), incubated with a blocking solution (5% Normal Goat serum (NGS), 0.5% bovine serum albumin (BSA), and 0.1% Triton X-1000 in PBS) for 1 h at room temperature (RT, with shaking). Then, slices were incubated overnight at RT (with shaking) with blocking solution supplemented with antibodies to probe for CREST (Rabbit, Proteintech, #12439-1-AP,1:300), NeuN (mouse, Abcam, ab104224, 1:250) or anti-HA tag (rabbit, Cell Signaling, #3724S). The following day, sections were washed in PBS (4 × 10 min), incubated with secondary antibodies (goat anti-rabbit Alexa Fluor 568, 1:500, A-11011; goat anti-mouse Alexa Fluor 488, 1:500, A-11008; ThermoFisher Scientific) in 0.1% Triton X-100 in PBS for 24 h at 4°C (with shaking), and washed in PBS (2 x 10min). Finally, the sections were counterstained with DAPI (1:10,000, Life Technologies; #D3571) for 15min at RT with shaking, washed in PBS (4 × 10 min), mounted onto Fisher Plus slides (Fisher Scientific, #12-550-15), and coverslipped with Aqua-Poly/Mount (Polysciences, # 18606-20). Images were acquired with a Leica TCS Sp8 confocal microscope under non-saturating conditions and processed with ImageJ (RRID:SCR_003070) or LAS X (Leica) to adjust channel colors, brightness, and contrast and applied to all experimental conditions. Low magnification micrographs were collected as a tilescan with a 10x objective at a single z-plane. High magnification micrographs were collected as z-stacks with either a 20x oil objective at 1.04 μm/z-step with 10 z-steps or a 63x oil objective at 0.43 μm/z-step with 47 z-steps and shown as a Maximum Intensity Projection processed in LAS X (Leica, RRID:SCR_013673). Conditions were compared within each slide and all images were acquired with the same gain/intensity. Pseudo-coloring for merged channels was used when possible to produce color-safe images and the same brightness and contrast settings are applied across experimental conditions for representative micrographs. Scale bars are included and described in figure legends. To examine CREST knockdown with the morpholino approach we used an intra-sample comparison analysis such that mice received unilateral infusion of the anti-Crest morpholino in one hemisphere and the standard negative control morpholino in the contralateral hemisphere. Tilescans were collected for 3 slices per animal (N=7) with a 10x objective.

The following alternative protocol was used for immunostaining of CBP (Rabbit, 1:500, Proteintech, 22277-1-AP) or CREST (Rabbit, 1:300, Proteintech, 12439-1-AP). Brains were flash frozen in isopentane, then sliced into 20-μm- thick sections around the injection site. After slide mounting, tissue was fixed in 4% PFA, followed by incubation in 10% NGS and 0.5% Triton-X in PBS. Primary antibody was diluted in 1% BSA in PBS and incubated overnight at 4°C. The tissue was washed with PBS and then incubated for 1 hour at room temperature in secondary antibody (1:500, Invitrogen AB_143157), followed by DAPI counterstaining (1:10,000). After mounting, slides were imaged using a Leica TCS SP8 confocal microscope at 20X and processed using ImageJ software. Conditions were compared within each slide and all images were acquired with the same gain/intensity. See key resources table.

#### Immunoblotting

To detect CREST protein expression, the dCA1 was collected 4days post-infusion (anti-Crest morpholino) (Figures 1A and 1B) and 1hr after the end of the OLM training period (i.e., molecular window of memory consolidation) (Figure 3D) (HC v. OLM-T10) (refer to Data S1 files for original immunoblot images).^27,28,36^ Hippocampal dissections were homogenized with a pestle in a mixture of Halt Protease Inhibitor (ThermoFisher, 87786) diluted to 1X in RIPA (Boston BioProducts, BP-115). Total protein lysate concentrations were determined with a Bradford assay (Bio-Rad, #5000005). A mixture of 60μg of total protein, NuPAGE LDS Sample Buffer (4x) (Invitrogen, #NP0007), Reducing buffer (10x) (Invitrogen, #NP0009) was prepared per sample replicates and denatured at 100° for 10min before loading to each lane of a 10% Bis-Tris NuPAGE (Invitrogen, #NP0301) precast gel. Gels ran for 20 minutes at 120V, then for 50 min at 200V, followed transfer onto a PVDF membrane (Millipore, #IPFL00010) at 35V at 4°C for 1hr using the XCell SureLock Mini-Cell and XCell II Blot Module system (Invitrogen, #EI0002). Then, the membranes were incubated for 1hr blocking buffer (5% BSA in TBS-Tween 0.1%), followed by primary antibodies diluted in blocking buffer (rabbit anti-Crest 1:3000, Proteintech, #12439-1-AP; mouse anti-GAPDH 1:800, Santa Cruz, #sc32233) incubation overnight at 4°C. The following day the membranes were washed in 0.1% TBS-Tween and followed with incubation in secondary antibodies (1:15,000 mouse anti-Rabbit 680LC, Li-COR, #926-68021; 1:15,000 goat anti-mouse 800CW, Li-COR, #926-32210) diluted in blocking buffer and a series of final washes in 0.1% TBS-Tween. Membranes were scanned with the Odyssey 2800 imager and protein expression was analyzed with Image Studio Lite (v3.1) (Li-COR). See key resources table.

#### RTq-PCR

Quantitative real-time RT-PCR was performed to examine the expression of *cFos, Crest, Cbp* and *Hprt* (reference gene). The dorsal hippocampus (dCA1) tissue was rapidly dissected for RNA isolation using the RNeasy minikit (Qiagen, #74104), including on-column DNase treatment with the RNase-Free DNase Set (Qiagen, #79256). The cDNA was generated using 100ng of total RNA with the High-Capacity cDNA Reverse Transcription Kit with RNase inhibitor (Thermo Fisher, #4374966). The primers and probe sets were derived from the Roche Universal Probe Library or the Integrated DNA Technologies PrimerQuest Tool and PrimeTime qPCR Probe Assay and designed to be exon-exon spanning (see Table S1 for primer and probe sequences) and multiplexed in the Roche Light-Cycle 480 II machine (Roche, LightCycler480 software version 1.5.1.62 SP3). All values were normalized to *Hprt* expression. Data analyses and statistics were conducted using the Roche proprietary algorithm based on the Pfaffl method.^107^

#### Chromatin immunoprecipitation

Chromatin immunoprecipitation was performed using dorsal hippocampus tissue and was processed as described in the Millipore EZ Magna ChIP kit protocol.^28,36,48^ Briefly, tissue was cross-linked in 1% formaldehyde, subjected to a cell lysis step followed by nuclear lysis. Sonication was performed on ice, using a hand-held sonicator (Fisher Scientific, Sonic Dismembrator Model 100) with 5X, 20’’ second pulses with at least one minute off in between. 50 microliters of chromatin (equivalent to 1/8 of the total) was used per IP. 4μg of anti-Crest antibody (Proteintech, Cat# 12439-1-AP) or 4μg of anti-rabbit IgG (Proteintech, cat# 30000-0-AP) was used for IPs. After washing, chromatin was eluted in the presence of Proteinase K followed by column purification of the DNA (Qiagen, QIAquick PCR Purification Kit, cat #28106). Primer sequences for *Egr2, c-Fos, Hpca* and *H4c11* (aka, *Hist1h4j*) that were used for PCR are provided in Table S1. Percent input was calculated as 100*AE ^(Ct(Input)- Ct(IP)).

#### RNA sequencing and alignment

To examine learning-induced gene expression changes during memory consolidation, we expressed the Y397F (null, blue), Y397D (mimic, green) or vehicle control (VEH, jetPEI only, orange and purple) in the dCA1 and subjected adult mice to either a subthreshold or threshold OLM training period (Figure 5A). The dCA1 tissue was harvested from n=6 male mice/experimental condition 1hr after the end of the training period and snap frozen. Mice assigned as home-cage controls only received handling prior to dCA1 collection. This RNA-seq experiment consisted of a total of 30 samples and 5 distinct experimental conditions collected only from male adult C57BL/6J mice. Total RNA was isolated from frozen hippocampi with the RNAeasy Plus mini kit (Qiagen, #74104). A RNA integrity number (RIN) was measured for each sample with the Qubit RNA HS assay kit (Invitrogen, Q32852), and only samples with a RIN ≥ 7.0 were included for submission to Novogene (Sacramento, CA) for cDNA synthesis, amplification, library construction, and sequencing using the Illumina NovaSeq and HiSeq platforms with paired-end 150bp (PE150) sequencing. The RNA-seq analysis was conducted using the Tuxedo pipeline. Tophat was used for alignment, and Cufflinks was used for assembly and expression level computation.^98,99^ Tophat aligned the RNA-seq reads against the mm10 reference genome and its transcriptome.^98^ Then, Cufflinks assembled the aligned reads to get the gene expression levels, with the output being fragments per kilobase of the exon model per million reads mapped (FPKM).^108^ The quality of sequences was evaluated using the mean PHRED quality scores for each replicate produced in real-time during the base-calling step of the sequencing run.

#### Differential gene expression

The differential analysis was performed by Cyber-T, using the ANOVA mode, which considers all conditions simultaneously.^55,109^ Cyber-T corrects naive p-values by employing an adjustable window of genes with similar expression levels and an adjustable background-strength parameter, deriving posterior distributions and more accurate standard deviation estimates. Following this, the genes were sorted by their corrected p-value, and analysis was confined to genes with a p-value of < 0.05. Cyber-T also produces a receiver operating characteristic (ROC) curve with an area under the curve (AUC) metric. As shown in supplementary Figure S8D, our analysis achieved an AUC of 0.978, a promising result for this type of biological experiment.

For completeness, we also performed the differential analyses using DESeq2.^56^ The list of genes identified by DESeq2 with a p-value < 0.05 is provided in supplementary Table S2. Although most Cyber-T DEGs are present in the DESeq2 output, DESeq2 identified additional genes, indicating that Cyber-T adopts a more conservative approach in assigning p-values. Specifically, between 64% and 77% of the Cyber-T DEGs in comparisons of Y397F-T10, Y397D-T3, Veh-T3, and Veh-T10 versus Veh-HC were also identified by DESeq2.

Volcano plots show DEGs for each comparison, where scattered points represent genes: the x-axis is the fold change, while the y-axis is the −log10(p-value). Colored dots refer to genes with a p-value < 0.05 generated by Cyber-T.^55^ Heatmaps display the numbers of DEGs and the magnitude of log2(fold-change) within each comparison. Gene ontology analysis was conducted using the DAVID Knowledgebase (v2023q4, NIH DAVID Bioinformatics), and the unadjusted p-values from the EASE score (DAVID’s modified Fisher’s Exact test) are used to report gene-enrichment in annotation terms.^110–113^

#### Hippocampal slice preparation and recording

Hippocampal slices were prepared from male WT mice (approximately 3 months of age). Following isoflurane anesthesia, mice were decapitated, and the brain was quickly removed and submerged in ice-cold, oxygenated dissection medium containing (in mM): 124 NaCl, 3 KCl, 1.25 KH2PO4, 5 MgSO4, 0 CaCl2, 26 NaHCO3, and 10 glucose. Coronal hippocampal slices (320 μm) were prepared using a Leica vibrating tissue slicer (Model: VT1000S) before being transferred to an interface recording containing preheated artificial cerebrospinal fluid (aCSF) of the following composition (in mM): 124 NaCl, 3 KCl, 1.25 KH2PO4, 1.5 MgSO4, 2.5 CaCl2, 26 NaHCO3, and 10 glucose and maintained at 31 ± 10C. Slices were continuously perfused with this solution at a rate of 1.75-2 ml/min while the surface of the slices were exposed to warm, humidified 95% O2 / 5% CO2. Recordings began following at least 2 hours of incubation.

Field excitatory postsynaptic potentials (fEPSPs) were recorded from CA1b stratum radiatum using a single glass pipette filled with 2M NaCl (2-3 MΩ) in response to orthodromic stimulation (twisted nichrome wire, 65 μm diameter) of Schaffer collateral-commissural projections in CA1 stratum radiatum. Pulses were administered at 0.05 Hz using a current that elicited a 50% maximal response. Paired-pulse facilitation was measured at 40, 100, and 200 sec intervals prior to setting baseline. After establishing a 20 min stable baseline, long-term potentiation (LTP) was induced by delivering 5 ‘theta’ bursts, with each burst consisting of four pulses at 100 Hz and the bursts themselves separated by 200 msec (i.e., theta burst stimulation or TBS). The stimulation intensity was not increased during TBS. Data were collected and digitized by NAC 2.0 Neurodata Acquisition System (Theta Burst Corp., Irvine, CA) and stored on a disk.

Data in the text are presented as means ± SD, while in the figures as mean ± SEM. The fEPSP slope was measured at 10–90% fall of the slope and data in figures on LTP were normalized to the last 20 min of baseline. Electrophysiological measures were analyzed using a 2-way ANOVA unless otherwise specified in the text and the level of significance was set at p ≤ 0.05.

### QUANTIFICATION AND STATISTICAL ANALYSIS

Statistical analyses were performed as stated in the main text and figure legends using Prism v10.0.2 (GraphPad). The quantification of immunoblots was performed using Licor Image Studio Lite (Version 3.1; RRID:SCR_013715) to collect band signal (i.e., Signal = Total Intensity - (Background Intensity x Area)) and reported as normalized values relative to a housekeeping protein (i.e., GAPDH) and the control condition. The quantification of fluorescence intensity from immunohistochemistry staining was performed using a defined area with the ImageJ (RRID:SCR_003070) Region of Interest (ROI) feature to sample the dCA1 brain region in all biological replicates for each experimental condition and reported as integrated density relative to the internal control condition in the contralateral brain hemisphere. The statistical methods used, including sample size, are based on previously published work with no additional methods to predetermine sample size, but we followed what is routinely reported in the field and reported it for each figure in the corresponding figure legend. The data distribution was assumed as normal, with similar variance among sample groups without any formal testing. All two-group comparisons were analyzed by two-tailed Student’s t-test, except for the quantitative analysis of immunohistochemistry fluorescence signal which used a two-tailed paired-Student’s t-test. When two independent factors were compared (i.e., treatment and session), data were analyzed with two-way ANOVAs with Sidak’s post-hoc test for multiple comparisons. All statistical analyses are two-tailed, unless otherwise stated in figure legends, with α value set to 0.05 and a p-value less than α required for statistical significance (* p < 0.05, ** p < 0.01, *** p < 0.001, **** p < 0.0001). The error bars in all figures represent Standard error of the mean (SEM). For all experiments, any sample values with a ± 2-standard deviations from the group mean were defined as outliers and excluded from data plots. Statistical analyses of LTP data used number of slices *in lieu* of number of animals as replicate data points.

FET was used to assess the significance of the intersection between pairs and groups of gene sets, which were identified based on their expression profiles across different conditions. This test is particularly suited for small sample sizes. The test compares the observed distribution of genes across conditions to what would be expected by chance, providing a p-value indicative of the likelihood of observing such an overlap if no actual association exists. For each comparison, we constructed 2x2 contingency tables containing the number of genes exclusively expressed in one condition, exclusively in another, shared between conditions, and not observed in either condition. These counts were then input into the FET to compute the p-value, reflecting the probability that the observed overlap (or greater) could occur under the null hypothesis of no association. We performed two versions of the test with a different number of background genes (not observed in either condition) and we report both results. Background1 contains all the genes with low expression values (<1) in the FPKM table, which resulted in 14434 genes. Background2 contains all the DEGs from both conditions. Given the multiple comparisons inherent in differential gene expression analysis, we applied a False Discovery Rate (FDR) Benjamini-Hochberg (BH) procedure to the p-values obtained from FET. For each set of comparisons, p-values from FET were collated and subjected to the BH procedure (Table S6). *Implementation Details*: The statistical analyses were implemented in Python, utilizing libraries such as Pandas for data manipulation, StatsModels for FDR correction, and SciPy for conducting Fisher’s Exact Test.

## Supplementary Material

Figures_S1-S16

Data_S1

Table_S1

Table_S2

Table_S3

Table_S4

Table_S5

Table_S6

Table_S7

Table_S8

Table_S9

Table_S10

Table_S11

Table_S12

Table_S13

Table_S14

Table_S15

Table_S16

Table_S17

Table_S18

Table_S19

Table_S20

Table_S21

Table_S22

Table_S23

Table_S24

Data_S2

Supplemental information can be found online at https://doi.org/10.1016/j.celrep.2026.117158.

## Figures and Tables

**Table:** KEY RESOURCES TABLE

REAGENT or RESOURCE	SOURCE	IDENTIFIER
Antibodies		
anti-CBP	Proteintech	Cat# 22277-1-AP; RRID:AB_3085692
anti-CREST	Proteintech	Cat# 12439-1-AP; RRID:AB_2302517
anti-NeuN	Abcam	Cat# ab104224; RRID:AB_10711040
anti-HA	Cell Signaling	Cat# 3724S; RRID:AB_1549585
Goat anti-Rabbit Alexa Fluor 568	Thermo Fisher Scientific	Cat# A-11011; RRID:AB_143157
Goat anti-Mouse Alexa Fluor 488	Thermo Fisher Scientific	Cat# A-11008; RRID:AB_143165
DAPI	Life Technologies	Cat# D3571; RRID:AB_2307445
anti-GAPDH	Santa Cruz Biotechnology	Cat# sc-32233; RRID:AB_627679
IRDYE 800CW Mouse secondary	Licor	Cat# 926-32210; RRID:AB_621842
IRDYE 680LT Rabbit secondary	Licor	Cat# 926-68021; RRID:AB_10706309
Bacterial and virus strains		
AAV1-CMV-Cre	Addgene	RRID: 10553
AAV1-CMV-EV	UCI Center for Neural Circuit Mapping (CNCM) Viral Core; RRID:SCR_025573	MW87
AAV1-miniCaMK2-hBG-HA-CREST-Y397F	UCI Center for Neural Circuit Mapping (CNCM) Viral Core; RRID:SCR_025573	MW276
AAV1-miniCaMK2-hBG-HA-CREST-Y397D	UCI Center for Neural Circuit Mapping (CNCM) Viral Core; RRID:SCR_025573	MW277
AAV1- miniCaMK2-hBG-EV	UCI Center for Neural Circuit Mapping (CNCM) Viral Core; RRID:SCR_025573	MW270
Biological samples		
adult C57BL/6J mouse hippocampal tissue	This paper	N/A
adult C57BL/6J mouse whole brain (flash-frozen, isopentane)	This paper	N/A
Healthy adult C57BL/6J mouse whole brain (PFA-perfused)	This paper	N/A
Chemicals, peptides, and recombinant proteins		
In vivo-jetPEI	Polyplus	Cat# 101000040
Paraformaldehyde	Sigma-Aldrich	Cat# 441244
Tween-20	Sigma-Aldrich	Cat# P7949
D-sucrose	Fisher Scientific	Cat# BP220-1
BSA standard	Fisher Scientific	Cat# 23209
BSA (lyophilized)	Fisher Scientific	Cat# 9048-46-8
Sodium pentobarbital	Sigma-Aldrich	Cat# P3761
Critical commercial assays		
RIPA lysis buffer	Thermo Fisher Scientific	Cat# 89900
NuPAGE sample reducing buffer (10x)	Thermo Fisher Scientific	Cat# NP0009
NuPAGE antioxidant reagent	Thermo Fisher Scientific	Cat# NP0005
NuPAGE Transfer buffer (20x)	Thermo Fisher Scientific	Cat# NP00061
NuPAGE MOPS running buffer	Thermo Fisher Scientific	Cat# NP001
LICOR Chameleon Due protein ladder	Licor	Cat# 928-60000
Bradford Protein Assay dye concentrate	BioRad	Cat# 5000005
Invitrogen NuPAGE precast protein gels	Thermo Fisher Scientific	Cat# NP0321BOX
PrimeTime Gene expression Mastermix (2x)	IDT	Cat# 1055771
High-capacity cDNA Reverse Transcription Kit with Rnase Inhibitor	Thermo Fisher Scientific	Cat# 4374966
GeneArt Site-Directed Mutagenesis system	Thermo Fisher Scientific	Cat# A13282
NEBuilder HiFi DNA Assembly Master Mix	NEB	Cat# E2621S
Qiagen RNeasy Mini kit	Qiagen	Cat# 74104
Qiagen Plasmid Plus Maxi kit	Qiagen	Cat# 12965
Deposited data		
Raw and analyzed data	This paper	GEO: GSE268687
Experimental models: Organisms/strains		
Mouse: C57BL/6J	The Jackson Laboratory	RRID:IMSR_JAX:000664
Mouse: Cbp^fl/fl^	Kang-Decker et al. 2004	RRID:IMSR_JAX:025178
Oligonucleotides		
Morpholino: standard negative control CCTCTTACCTCAGTTACAATTTATA	Gene Tools	Cat# PCO-VivoStandardControl-100
Morpholino: anti-Crest ACTCAGCCCGTGATCATTGGG	Gene Tools	Cat# mSS18L-42-12Jul22A-V-K
Primers for RTqPCR and ChIPqPCR, See Table S1	This paper	N/A
Forward primer for Site-Directed Mutagenesis, CREST Y397F: GGGCCAGTTTGGAAATTACCAGCAATAAAAGCTT	This paper	N/A
Reverse primer for Site-Directed Mutagenesis, CREST Y397F: GGGCCAGTTTGGAAATTACCAGCAATAAAAGCTT	This paper	N/A
Forward primer for Site-Directed Mutagenesis, CREST Y397D: GGGCCAGGATGGAAATTACCAGCAATAAAAGCTT	This paper	N/A
Reverse primer for Site-Directed Mutagenesis, CREST Y397D: TTTCCATCCTGGCCCTGTTCATAGCC	This paper	N/A
Recombinant DNA		
pAAV-miniCaMK2-hBG-HA-CREST-Y397F	This paper	MW276
pAAV-miniCaMK2-hBG-HA-CREST-Y397D	This paper	MW277
Software and algorithms		
ImageJ	NIH	RRID:SCR_003070; https://imagej.net/ij/
Leica Application Suite LAS X	Leica	RRID:SCR_013673
Biorender	Biorender	RRID:SCR_018361; https://www.biorender.com/
Licor Image Studio Lite v5.2	Licor	Version 3.1; RRID:SCR_013715
PhosphoSite Plus v6.7.4	Cell Signaling Technologies	https://www.phosphosite.org/homeAction.action
NetPhorest2.1	Miller et al^42^	http://www.miller-lab.org/webservices.html
GraphPad Prism (for Windows) v10.0.2 (232)	GraphPad	RRID:SCR_002798
LightCycler 480 RW v1.5.1.62; SP3	Roche	N/A
Cyber-T	Kayala and Baldi^55^	https://cybert.ics.uci.edu
TopHat v2.1.1	Trapnell et al.^98^	https://ccb.jhu.edu/software/tophat/index.shtml
DESeq2 v1.42.0	Love et al.^56^	https://bioconductor.org/packages/DESeq2
Cufflinks v2.2.1	Trapnell et al.^99^	https://cole-trapnell-lab.github.io/cufflinks
DAVID Knowledgebase v2023q4	NIH DAVID Bioinformatics	RRID:SCR_001881
Mouse genome assembly GRCm38 (mm10)	UCSC Genome Browser	https://genome.ucsc.edu/cgi-bin/hgGateway?db=mm10
ANY-maze tracking software	Stoelting	RRID:SCR_014289
Video Freeze Video Fear Conditioning Software v3.0.0.0	Med Associates Inc	RRID:SCR_014574; https://med-associates.com/product/videofreeze-video-fear-conditioning-software/
Other		
cDNA synthesis, amplification, library construction, and sequencing using the Illumina NovaSeq and HiSeq platforms with paired-end 150bp (PE150) sequencing	Novogene	Sacramento, CA

## Data Availability

• The RNA-seq datasets generated in this study have been deposited to the NCBI Gene Expression Omnibus (GEO) under accession number GEO: GSE268687 and are publicly available as of the date of publication. • Original western blot images (Data S1) and the datapoints used to generate all graphs (Data S2) are provided as source data files. • This paper does not report original code • Any additional information required to reanalyze the data reported in this paper is available from the lead contact upon request.

## Highlights

- Duration of incidental learning changes gene composition without a proportional accumulation
- CREST Y397 bidirectionally regulates memory processes and transforms subthreshold learning
- CBP is necessary for facilitating the effects of CREST Y397 on long-term memory formation
- CREST Y397 can regulate IEGs, calcium signaling, and histone modifying/chaperoning processes
