# Supplementary material for: The nBAF complex subunit CREST/SS18L1 regulates hippocampal memory processes via tyrosine 397 and histone acetyltransferase CBP
Source: Cell Rep. Author manuscript; Available in PMC 2026 May 20. (PMC13189212; doi:10.1016/j.celrep.2026.117158)
Supplement: Figures_S1-S16 [file NIHMS2168525-supplement-Figures_S1-S16.pdf]

Cell Reports, Volume *45*

Supplemental information

The nBAF complex subunit CREST/SS18L1 regulates hippocampal memory processes via tyrosine 397 and histone acetyltransferase CBP

Franklin G. Garcia, Maria Farias de Albuquerque, Vanessa Johnson, Enikö Kramár, Kousha Changizi, Sherif Abdelkarim, Agatha S. Augustynski, Dina P. Matheos, Joseph Picone, Tracy L. Fetterly, Alyssa Rodriguez, Jessica Childs, Angela Gomez-Arboledas, Julia Winter, Elizabeth Heller, Pierre Baldi, and Marcelo A. Wood

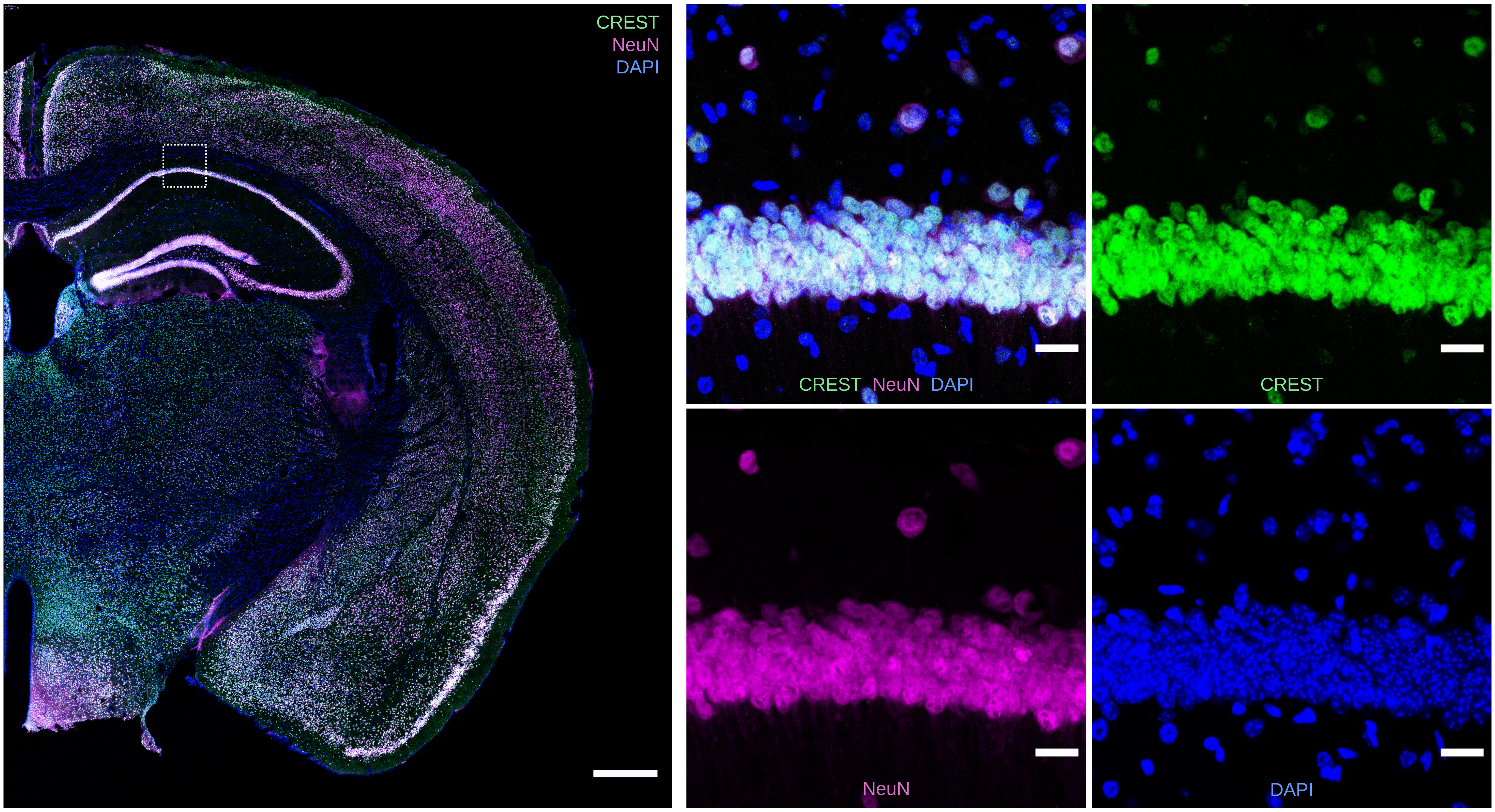

Figure S1.
CREST is highly expressed among neuronal populations of the dorsal hippocampus. Representative immunofluorescence micrographs with color-safe pseudo-coloring show expression of CREST (green) and NeuN (magenta) in a coronal section of the mouse brain (Bregma = -2.0). Nuclei are counterstained with DAPI (blue). Boxed area (white, left) selected for higher magnification (right) and shown with merged signals for CREST, NeuN and DAPI and provided with the individual color channel for each. Scale bar: low magnification (left, 500 μm) and higher magnification (right, 20 μm).

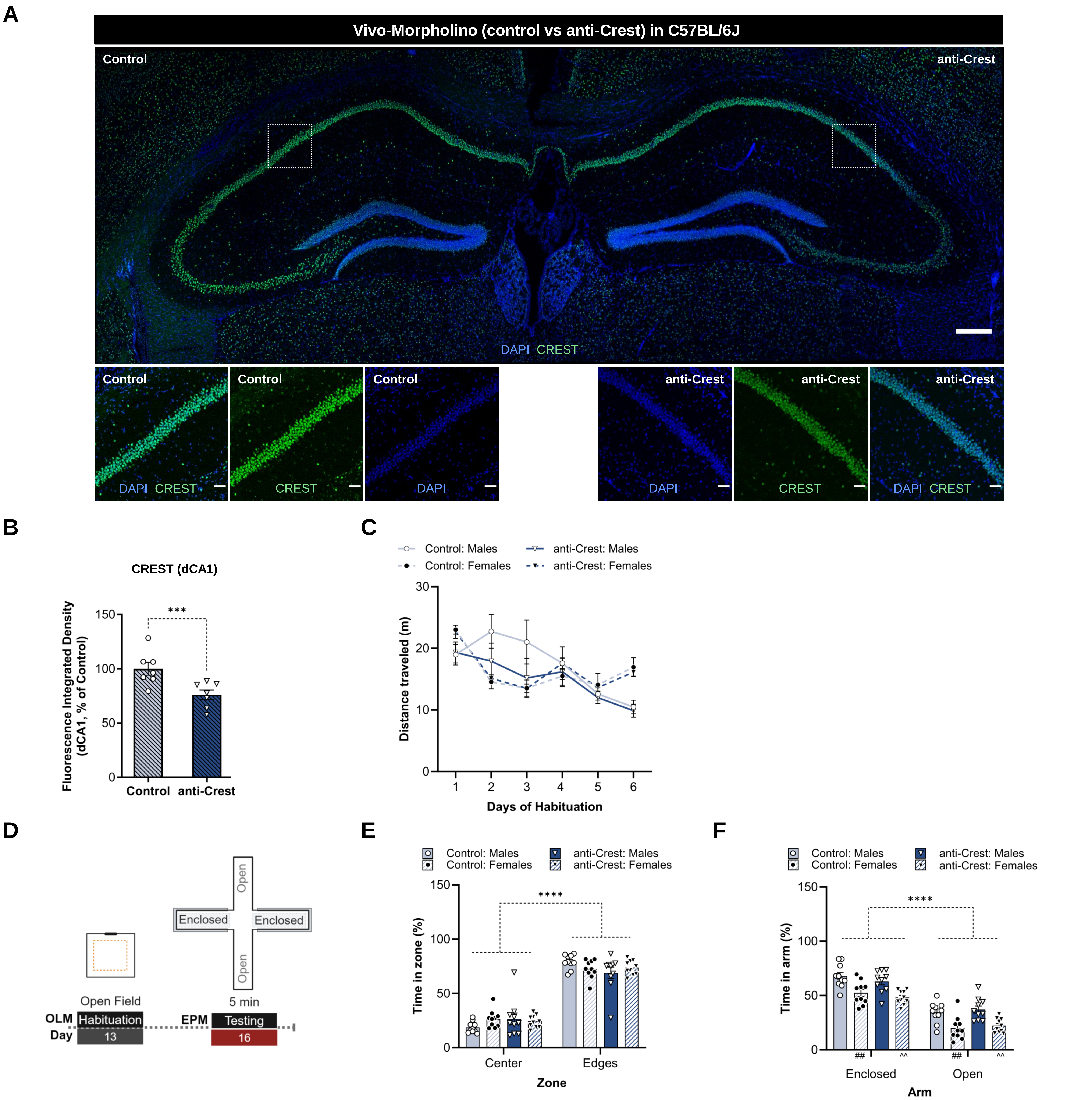

Figure S2.
Focal dorsal hippocampus knockdown of *Crest* has no effect on open field or elevated plus maze performance.
(A) Representative fluorescence micrograph shows an intra-sample comparison of both brain hemispheres for CREST (green) immunoreactivity 4 days after infusion of an anti-Crest (right hemisphere) or a standard control (left, contralateral hemisphere) vivo-morpholino (6μM) into the dorsal hippocampus (dCA1). Selected area (white) corresponds to high magnification insets for each condition. Nuclei are counterstained with DAPI (blue). Scale bar: low magnification (above, 250 μm) and high magnification (below, 50 μm). **(B)** Quantification of fluorescence integrated density of CREST protein expression in the dCA1 (two-tailed paired-Student's t-test: $t_6 = 7.10$, $^{***}p < 0.001$; $n = 7$ males/ condition; 3 slices per animal) 4 days after unilateral infusion of an anti-Crest morpholino and a control morpholino (contralateral hemisphere) into the dCA1 that is presented as a summary of two independent experiments. **(C)** Total distance travelled during OLM habituation for male and female C57BL6/J mice infused with either Control or anti-Crest vivo-morpholino (Two-way ANOVA: males (main effect of day $F_{5,108} = 6.41$, $^{****}p < 0.0001$; no interaction of condition x day, $F_{5,108} = 0.64$, $p = 0.67$) and females (main effect of day $F_{5,108} = 17.89$, $^{****}p < 0.0001$; no interaction of condition x day, $F_{5,108} = 0.41$, $p = 0.84$)). **(D)** Schematic of Open field testing on Day 13 of OLM (i.e., 6th day of habituation following post-surgery recovery day) and elevated plus maze (EPM, Day 16) testing 2 days after the end of OLM testing. **(E)** Percent of time in center or edge zones of the OLM chambers (i.e., open field task) in male and female C57BL6/J mice (Effect of zone only: Two-way ANOVA: $F_{1,72} = 475.4$, $^{****}p < 0.0001$). No group differences were observed within zone. **(F)** Percent of time in either the opened or closed arms in the elevated plus maze (Effect of arm only: Two-way ANOVA: $F_{1,72} = 187$, $^{****}p < 0.0001$; Sidak's post-hoc test: effect of sex within Control condition ($^{\#\#}p < 0.01$); effect of sex within anti-Crest condition ($^{\wedge\wedge}p < 0.01$). $n = 10$/ condition/ sex. Data presented as mean ±SEM. Group color-scheme: males (white circles and triangles) and females (black circles and triangles).

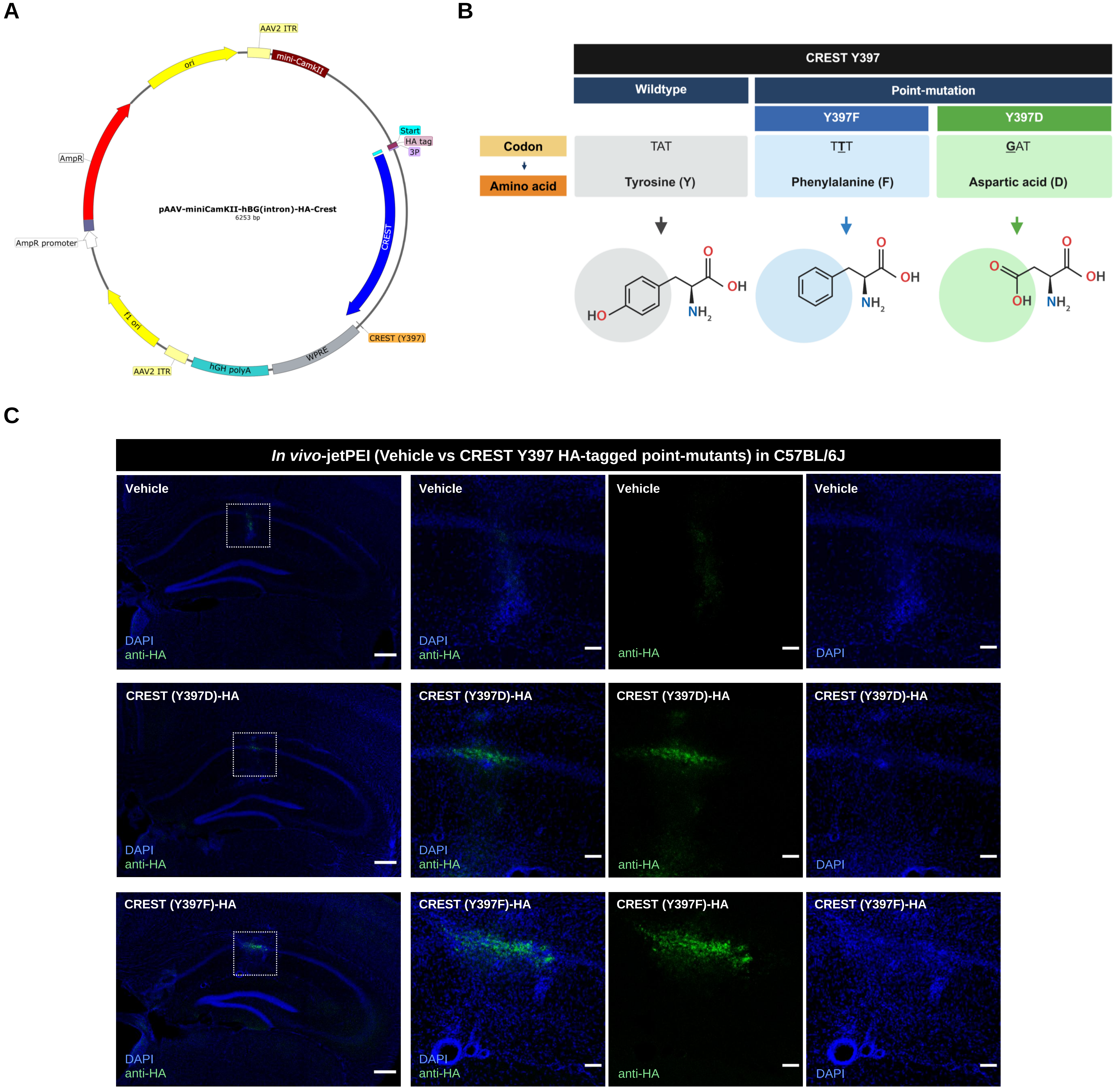

Figure S3.
Summary for generating CREST Y397 point-mutants.
(A) Map of a plasmid expressing *Crest* (i.e., *Ss18l1*) under the regulation of a CaMKII0.4 (mini) promoter (UCI CNCM Viral Core Facility) that was used for site-directed mutagenesis at Y397 (orange annotation) (Created with SnapGene). **(B)** Schematic summary for generating CREST Y397 point-mutants: 1. CREST Y397 (gray) is encoded by codon TAT (Y, tyrosine). Site-directed mutagenesis and Seamless cloning system (GeneArt-Thermo Fisher Scientific) was used to generate a Y397F point-mutant (TAT → TTT; Y → F, phenylalanine) and a Y397D point-mutant (TAT → GAT; Y → D, aspartic acid). **(C)** Representative immunofluorescence micrographs show immunoreactivity for the HA-tagged (green) CREST(Y397D)-HA and CREST(Y397F)-HA point-mutants (relative to the vehicle control condition) when complexed with the in vivo-jetPEI (Polyplus) transfection reagent at 4 days after infusion into the dCA1. Nuclei are counterstained with DAPI (blue). Selected area in dCA1 (white) corresponds to high magnification insets for each condition. Scale bar: low magnification (250 μm) and high magnification (50 μm).

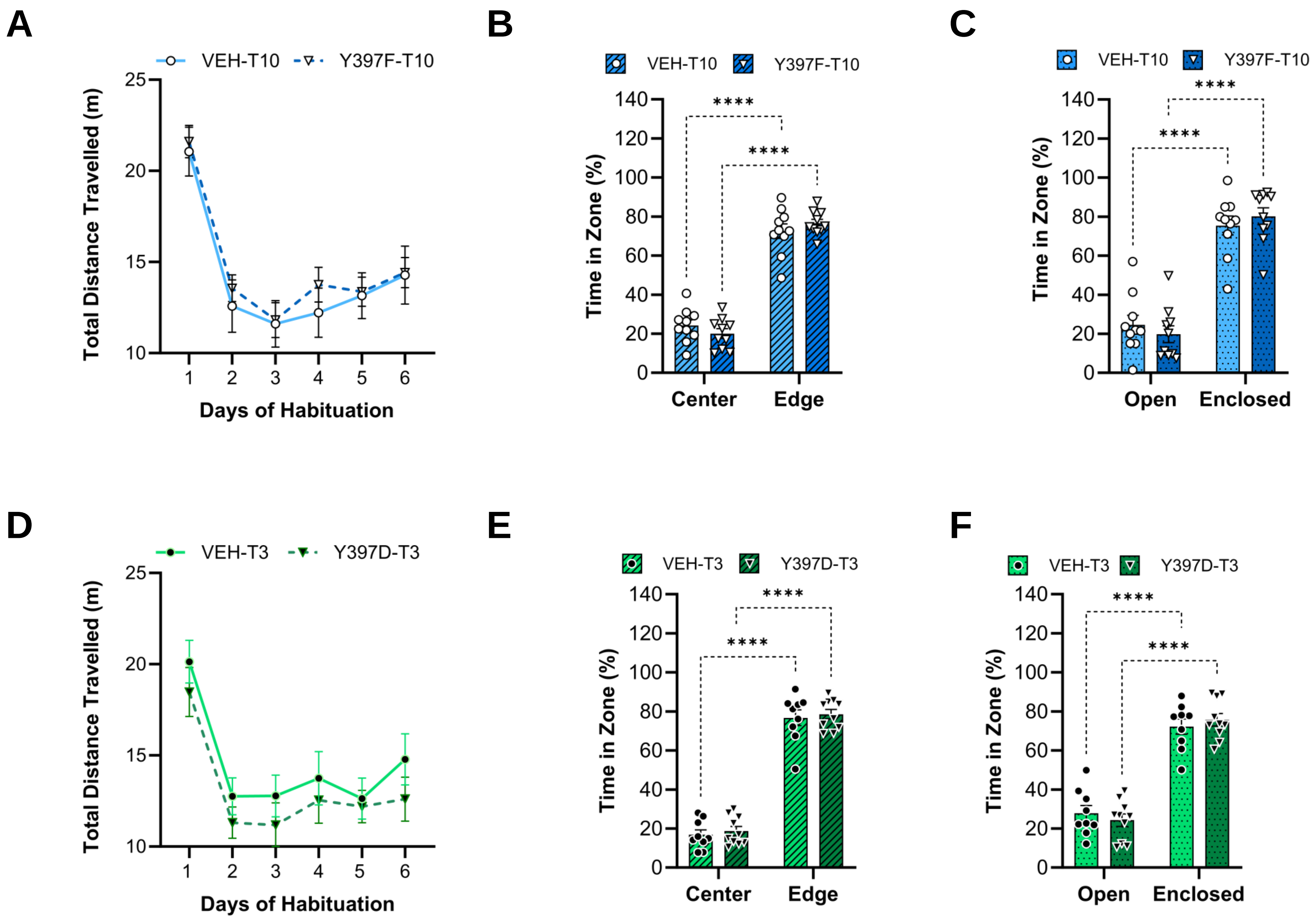

Figure S4.
Focal *in vivo* transfection of CREST Y397 point-mutants has no effect on movement during habituation or performance in the open field or elevated plus maze.
(A) Total distance traveled during days of habituation of OLM behavior: VEH-T10 and Y397F-T10 (Two-way ANOVA: main effect of day, $F_{5,108}$ = 18.23, **** $p < 0.0001$; no interaction of condition x day comparison, $F_{5,108} = 0.12$, p = 0.99)). **(B)** Percent of time in open field zones during Day 6 of OLM habituation: VEH-T-10 and Y397F-T10 (Two-ANOVA: effect of zone only, $F_{1,36}$ = 345.1, **** $p < 0.0001$). **(C)** Percent of time in elevated plus maze zones 24 hrs after OLM test: VEH-T10 and Y397F-T10 (Two-ANOVA: effect of zone only, $F_{1,36}$ = 148.0, **** $p < 0.0001$). **(D)** Total distance traveled during days of habituation of OLM behavior: VEH-T3 and Y397D-T3 (Two-way ANOVA: main effect of day, $F_{5,102}$ = 11.10, **** $p < 0.0001$; no interaction of condition x day, $F_{5,102} = 0.12$, p = 0.99). **(E)** Percent of time in open field zones during Day 6 of OLM habituation: VEH-T3 and Y397D-T3 (Two-ANOVA: effect of zone only, $F_{1,34}$ = 438.1, **** $p < 0.0001$), no condition differences within zone. **(F)** Percent of time in elevated plus maze arm zones at 1 day after OLM test: VEH-T3 and Y397D-T3 (Two-way ANOVA: effect of zone only, $F_{1,34}$ = 180.6, **** $p < 0.0001$). Control (VEH-T10, white circles; VEH-T3, black circles), Crest Y397F-T10 (white triangles) and Crest Y397D-T3 (black triangles). Data presented as mean ±SEM. Behavior: n = 9-10/ group (males only)/ independent replicate cohort. See **Figure S2D** for schematic of open field and elevated plus maze timeline relative to OLM behavior.

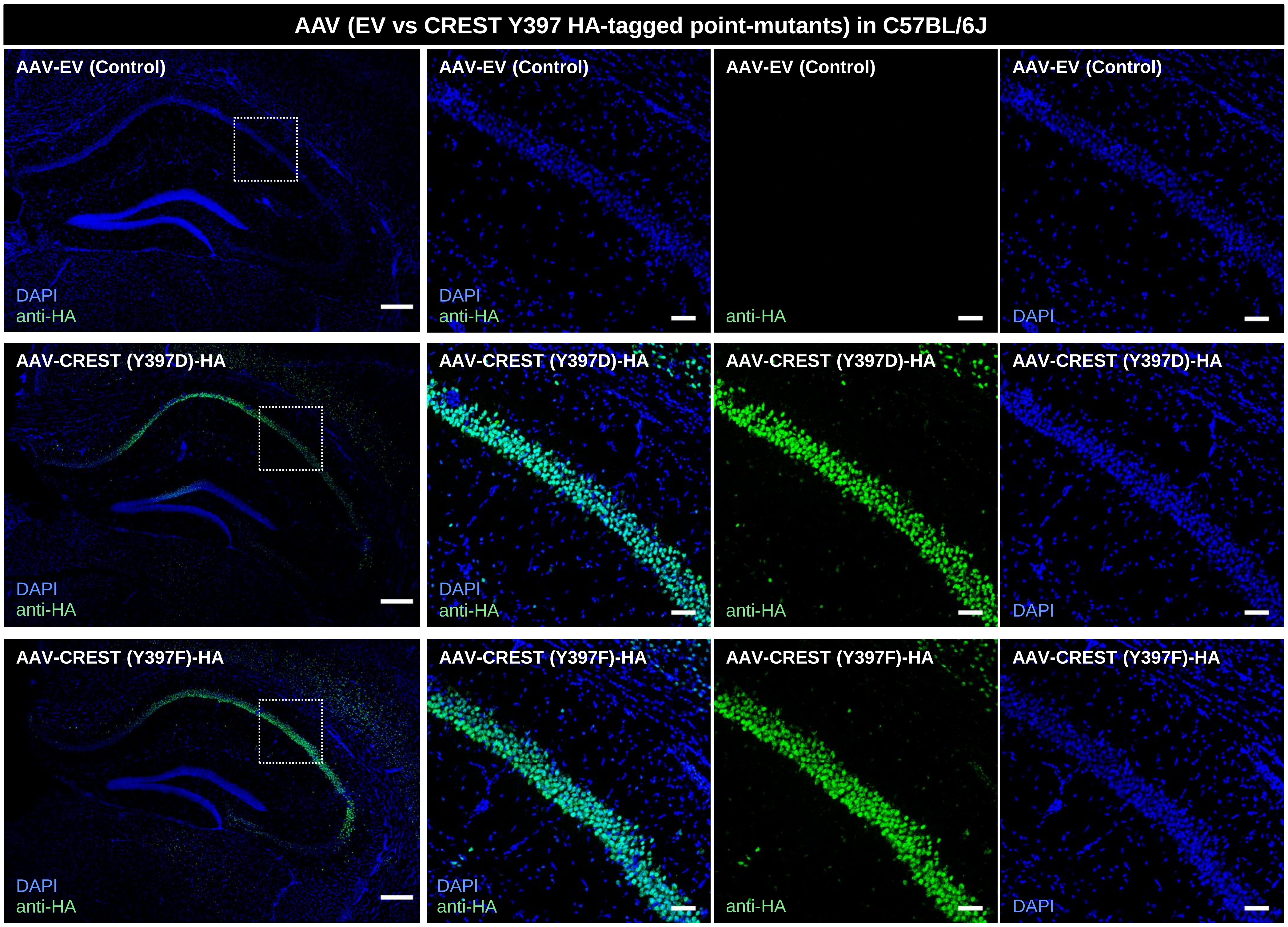

Figure S5.
AAV overexpression of the CREST Y397F and Y397D point-mutations in the adult dCA1.
Representative immunofluorescence micrographs show immunoreactivity for the HA-tagged (green) AAVs that express either the CREST Y397D or Y397F point-mutant 2 weeks after infusion into the dCA1. Selected area (white) corresponds to high magnification insets for each condition. An AAV empty vector (EV) was delivered as a control condition. Nuclei are counterstained with DAPI (blue). Scale bar: low magnification (250 μm) and high magnification (50 μm).

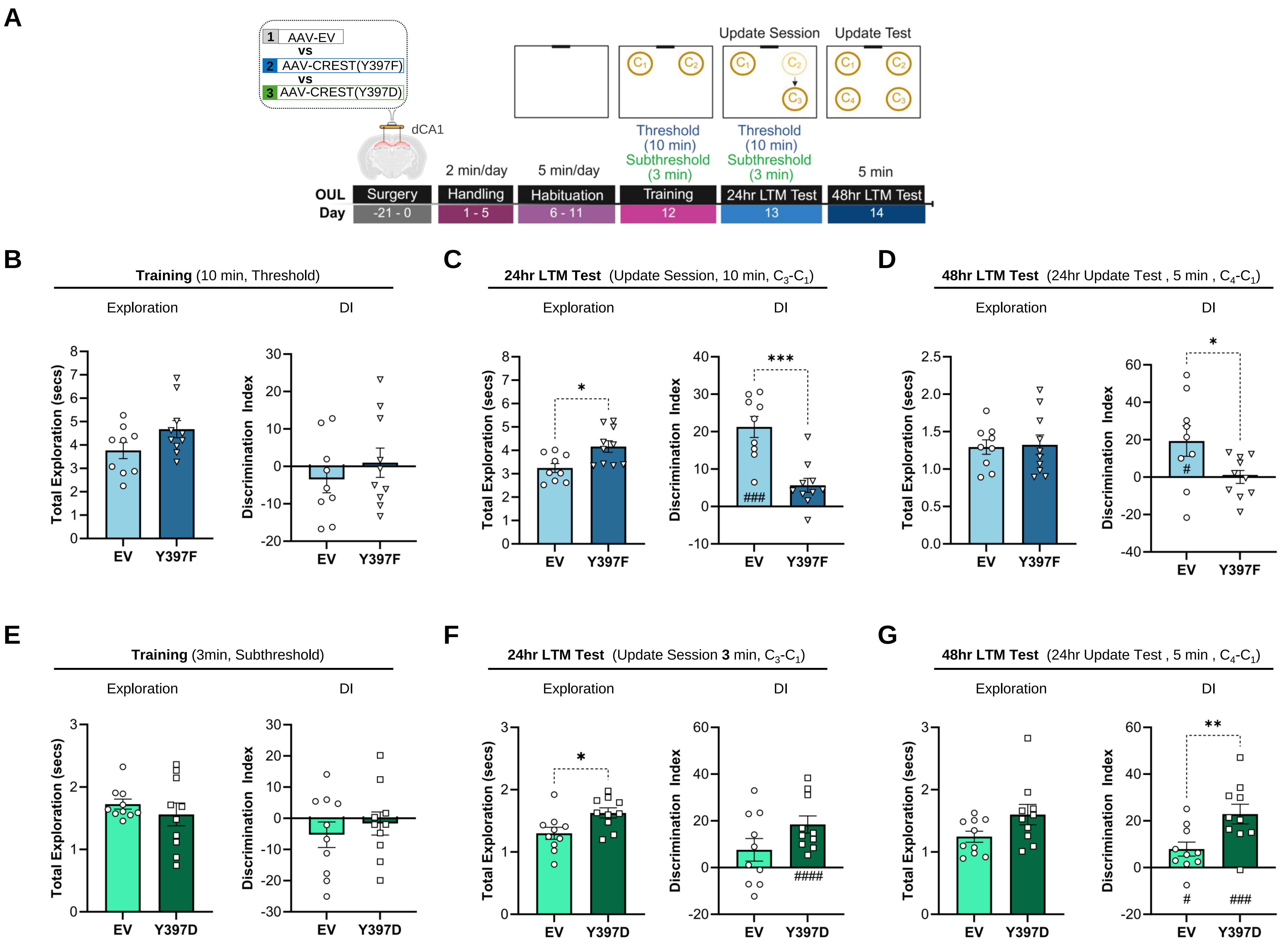

Figure S6.
CREST Y397 bidirectionally regulates hippocampal-dependent long-term memory for updated information.
(A) Timeline of the hippocampal-dependent Object in Updated Location task (OUL) where animals received bilateral dCA1 infusion of AAVs for empty vector control (EV), CREST(Y397F) or CREST(Y397D) 3weeks prior to the start of behavior and examined in either a threshold (T10, 10min) or subthreshold (T3, 3min) training conditions followed by a 24hr LTM test (Update session) and a 48hr LTM test (24hr Update test, 5min). **(B)** Total time of object exploration (Student's t-test: t_{17} = 1.80, p = 0.089) and DI (Student's t-test: t_{17} = 0.83, p = 0.42) in the 10 min threshold training session for EV and Y397F conditions. **(C)** Total time of object exploration (Student's t-test: t_{17} = 2.86, p = 0.012) and DI (Student's t-test: t_{17} = 4.72, $^{***}p$ < 0.001) in the 24hr LTM test (Update session,10 min) for EV and Y397F conditions. Training vs 24hr LTM test DI: EV (paired Student's t-test: t_8 = 5.20, $^{\#\#\#}p$ < 0.001), Y397F (paired Student's t-test: t_9 = 1.46, p = 0.18). **(D)** Total time of object exploration (Student's t-test: t_{17} = 0.18, p = 0.86) and DI (Student's t-test: t_{17} = 2.25, $^{*}p$ < 0.05) in the 48hr LTM test session (24hr Update test, 5 min) for the EV and Y397F conditions. Training vs 48hr LTM test DI: EV (paired Student's t-test: t_8 = 2.97, $^{\#}p$ < 0.05), Y397F (paired Student's t-test: t_9 = 0.15, p = 0.89). **(E)** Total time of object exploration (Student's t-test: t_{18} = 0.83, p = 0.42) and DI (Student's t-test: t_{18} = 0.65, p = 0.52) in the 3min subthreshold training session for EV and Y397D conditions. **(F)** Total time of object exploration (Student's t-test: t_{18} = 2.63, $^{*}p$ < 0.05) and DI (Student's t-test: t_{18} = 1.78, p = 0.092) in the 24hr LTM test (Update session, 3min) for EV and Y397D conditions. Training vs 24hr LTM test DI: EV (paired Student's t-test: t_9 = 1.67, p = 0.13), Y397D (paired Student's t-test: t_9 = 8.31, $^{\#\#\#\#}p$ < 0.0001) **(G)** Total time of object exploration (Student's t-test: t_{18} = 1.91, p = 0.072) and DI (Student's t-test: t_{18} = 2.91, $^{**}p$ < 0.01) in the 48hr LTM test session (24hr Update test, 5 min) for the EV and Y397D conditions. Training vs 48hr LTM test DI: EV (paired Student's t-test: t_9 = 2.34, $^{\#}p$ < 0.044), Y397D (paired Student's t-test: t_9 = 5.90, $^{\#\#\#}p$ < 0.001). Data presented as mean ±SEM. n = 9-10 /condition, all males. EV (white circles), Y397F (white triangles), and Y397D (white squares).

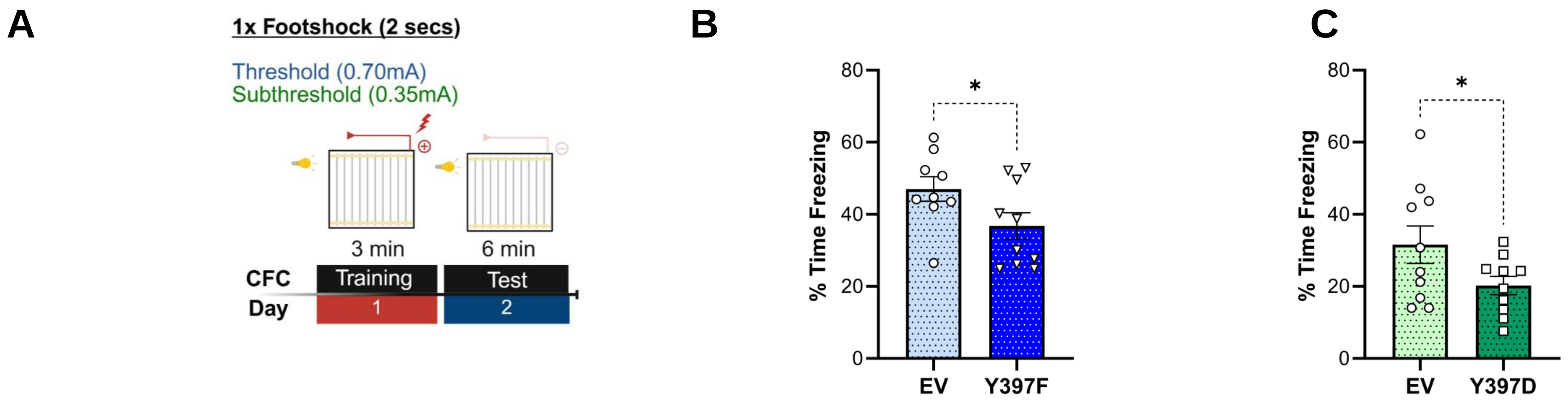

Figure S7.
AAVs of CREST Y397 point-mutants impair long-term memory for contextual fear.
(A) Schematic of procedure for hippocampal-dependent contextual fear conditioning to examine LTM after dCA1 expression of AAVs to express the Y397F or Y397D point mutation conditions and exposure to threshold (0.70mA shock) or subthreshold (0.35mA shock) training session (3min), respectively, relative to EV control. **(B)** Total % freezing during the 24hr LTM test (6min) for EV and Y397F AAV conditions (one-tailed Student's t-test: t_{17} = 2.04, $^{*}p < 0.05$). **(C)** Total % freezing during the 24hr LTM test (6min for EV and Y397D AAV conditions (one-tailed Student's t-test: t_{18} = 1.96, $^{*}p < 0.05$). Data presented as mean ±SEM. n = 9-10 /condition, all males. EV (white circles), Y397F (white triangles), and Y397D (white squares).

A

Condition #	Description of dCA1 infusion experimental conditions	Sample Sequencing IDs	Condition Abbreviation
1	in vivo-jetPEI reaction without exogenous DNA transfection (Vehicle) collected with only home-cage exposure	N01-N06	VEH-HC
2	in vivo-jetPEI reaction without exogenous DNA transfection (Vehicle) collected 1hr post-threshold training period (10min)	O01-O06	VEH-T10
3	in vivo-jetPEI transfection of CREST(Y397F) point mutation and collected 1hr post-threshold training period (10min)	P01-P06	Y397F-T10
4	in vivo-jetPEI reaction without exogenous DNA transfection (Vehicle) collected 1hr post-subthreshold training period (3min)	Q01-Q06	VEH-T3
5	in vivo-jetPEI transfection of CREST(Y397D) point mutation and collected 1hr post-subthreshold training period (3min)	R01-R06	Y397D-T3

B

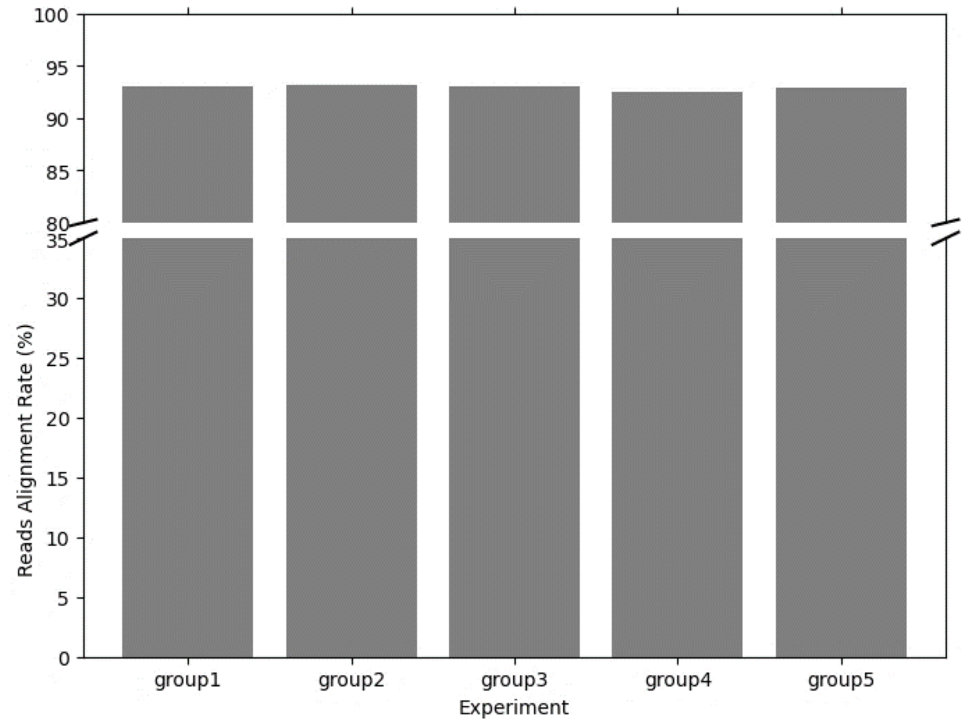

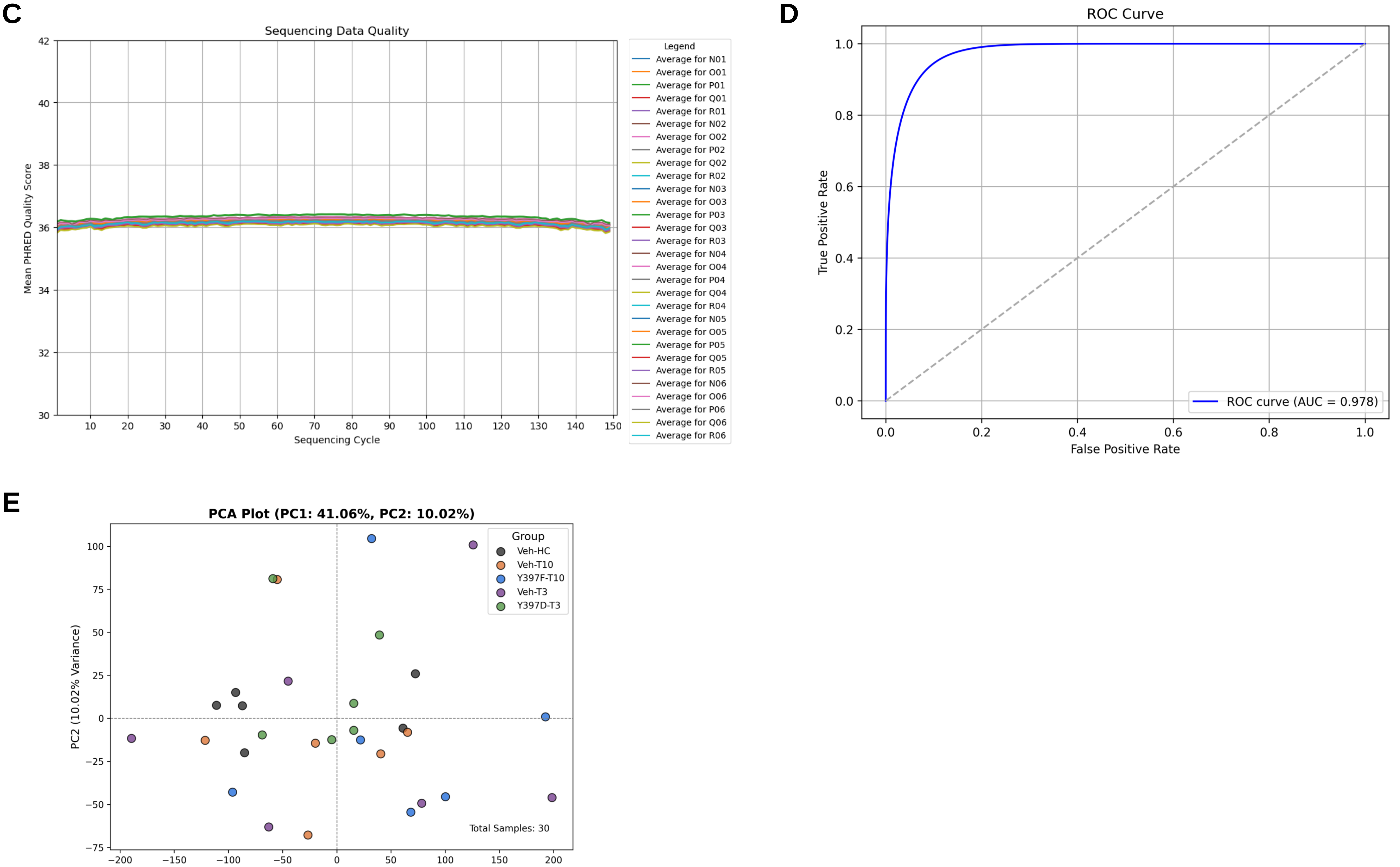

Figure S8.
RNA sequencing quality analysis.
(A) Summary of conditions, sample sequencing identification numbers and abbreviations for each sample group of RNA-seq dataset where a hemisphere of dissected dorsal hippocampus tissue was collected for *n* = 6 animals/group. **(B)** Plot of average Matched Reads for each experimental group reported as read alignment rate (%). **(C)** Mean PHRED quality scores for each sample. **(D)** ROC curve generated from Cyber-T analysis by modeling the distribution of p-values as a mixture of two beta distributions—a uniform component representing background (non-differentially expressed genes) and a peaked component near zero representing differentially expressed genes (described in Kayala and Baldi, 2012). **(E)** PCA plot shows the distribution of individual samples for each corresponding experimental condition based on their similarity.

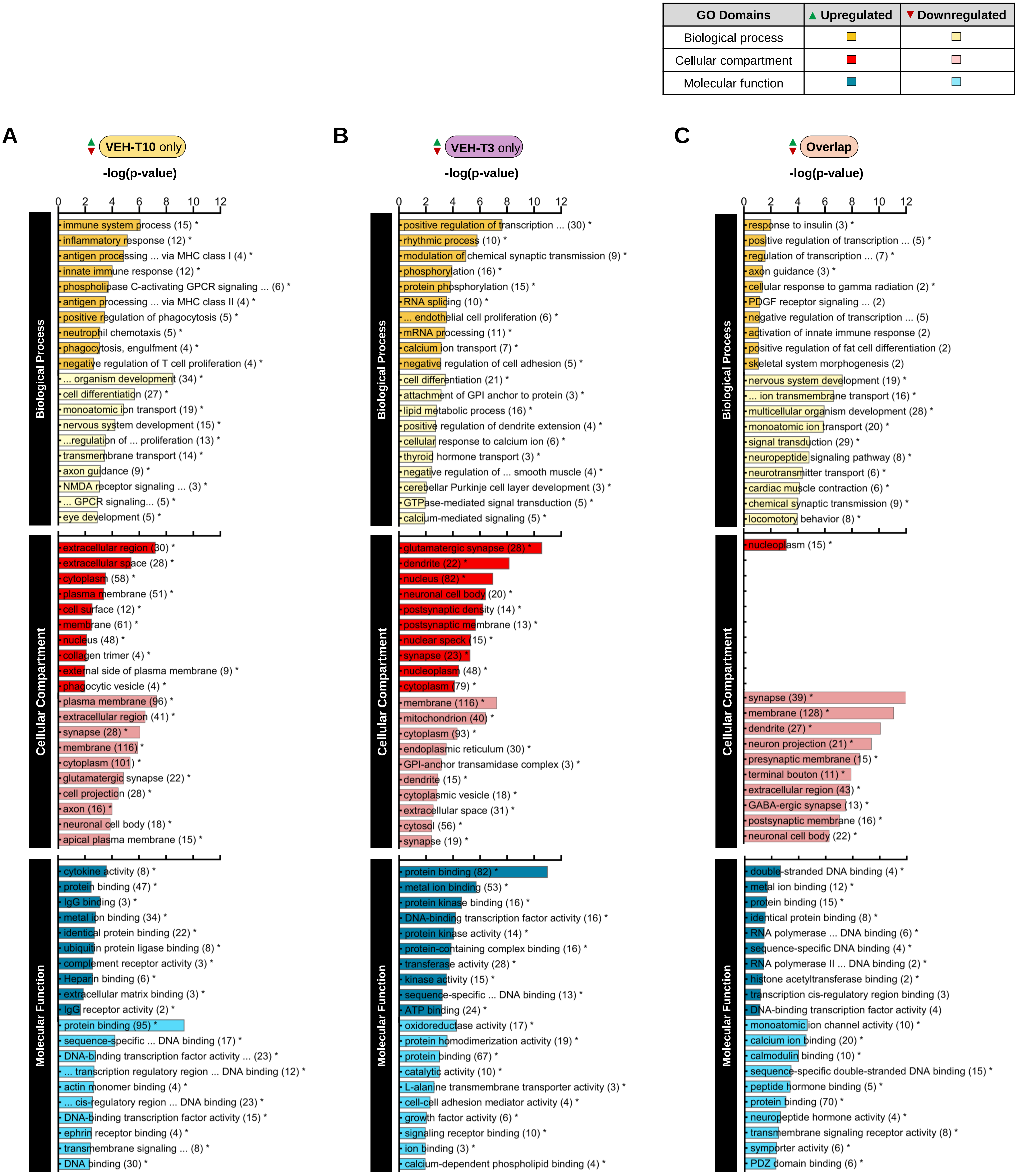

Figure S9.
DEGs expressed in threshold (T10) and subthreshold (T3) OLM training periods show distinct biological signatures.
Top 10 gene ontology (GO) terms from functional annotation and enrichment analysis of upregulated and downregulated DEGs (p < 0.05 cutoff) identified by the three distinct gene signature clusters in the two-way Venn diagram comparison of the VEH-T10 and VEH-T3 conditions (**Figure 5F**) and categorized by biological process, molecular function and cellular compartment GO domains: **(A)** VEH-T10 only, **(B)** VEH-T3 only, and **(C)** overlap. Fisher's exact p-value used to annotate significant gene-enrichment for an annotation term (* p < 0.05) (refer to **Table S5-S7)**. Upregulated GO terms = dark color scheme; and downregulated GO terms = light color scheme.

A

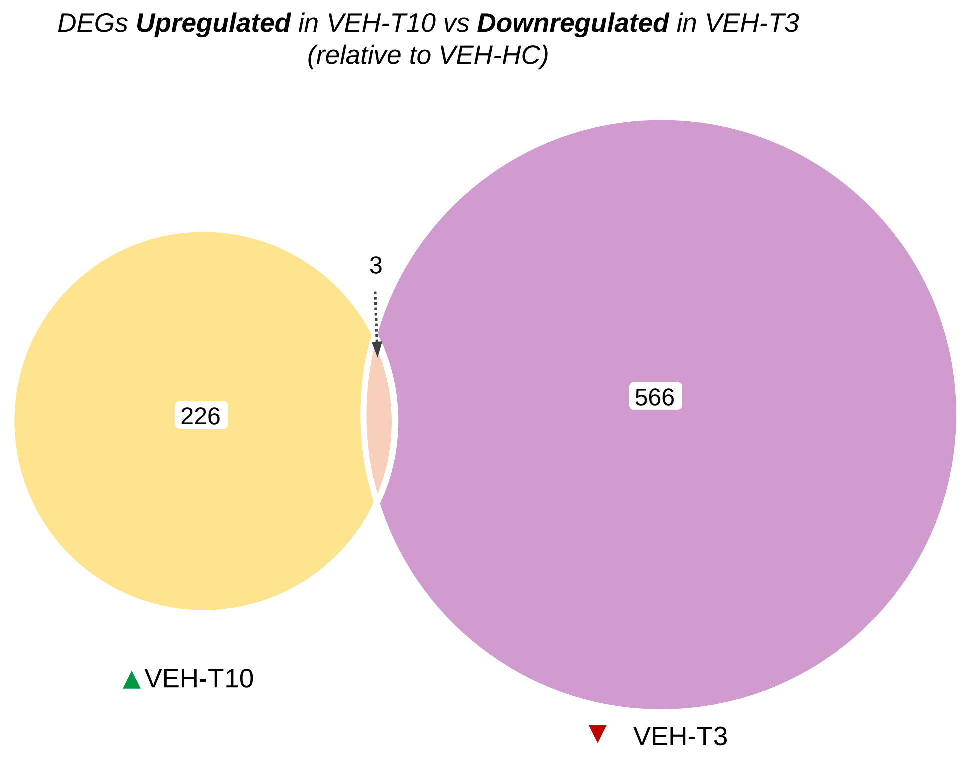

B

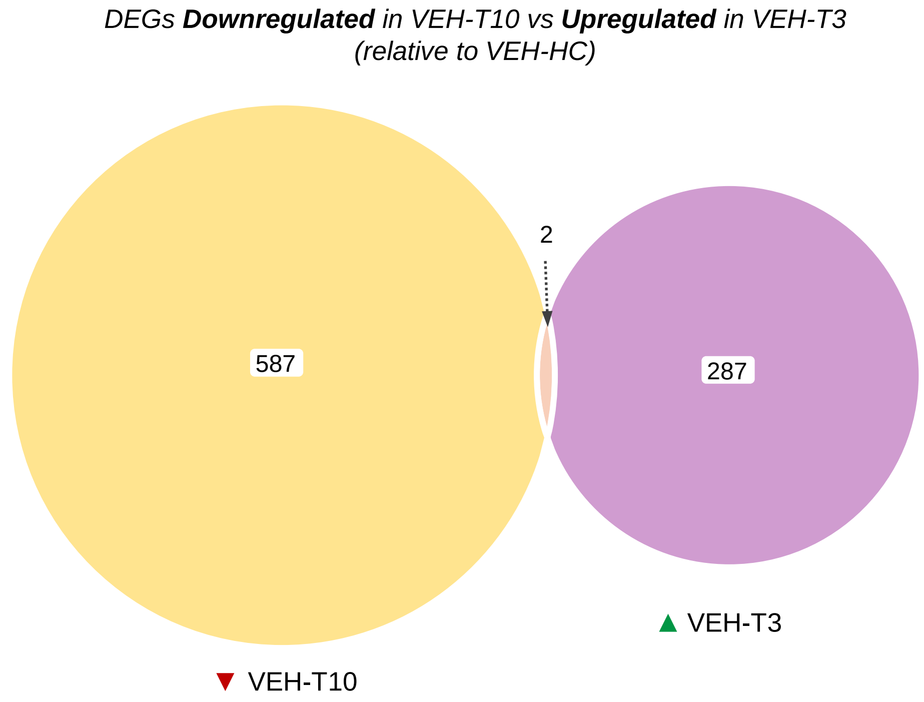

Figure S10.
DEGs with opposing direction of expression for threshold (T10) and subthreshold (T3) OLM training periods.
(A-B) Venn diagrams show the overlap in shared DEGs upregulated in VEH-T10 (threshold OLM training) but downregulated in VEH-T3 or downregulated in VEH-T10 but upregulated in VEH-T3. Number of significant DEGs noted for each section of the comparison ($p < 0.05$, relative to HC*) (see* **Table S8** and **Table S9)**.

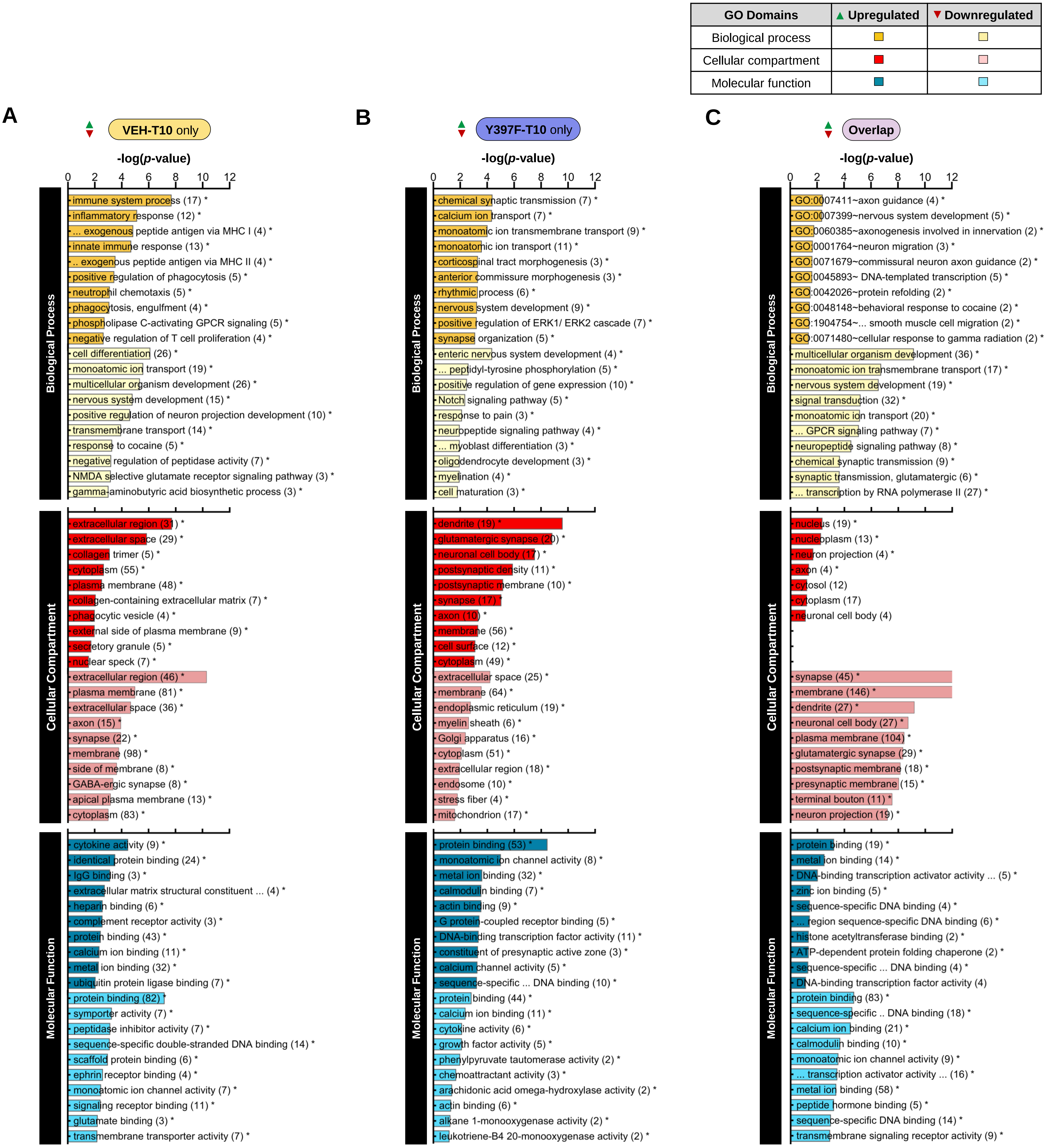

Figure S11.
DEGs affected by expression of the CREST Y397F point-mutant in a T10 OLM training period show distinct biological signatures.
Top 10 gene ontology (GO) terms from functional annotation and enrichment analysis of upregulated and downregulated DEGs ($p < 0.05$ cutoff) identified by the three distinct gene signature clusters in the two-way Venn diagram comparison of the VEH-T10 and Y397F-T10 conditions (**Figure 5G**) and categorized by biological process, molecular function and cellular compartment GO domains: **(A)** VEH-T10 only, **(B)** Y397F-T10 only, and **c,** overlap. Fisher's exact *p*-value used to annotate significant gene-enrichment for an annotation term (* p < 0.05) (refer to **Table S6, Table S11** and **Table S12**). Upregulated GO terms = dark color scheme; and downregulated GO terms = light color scheme.

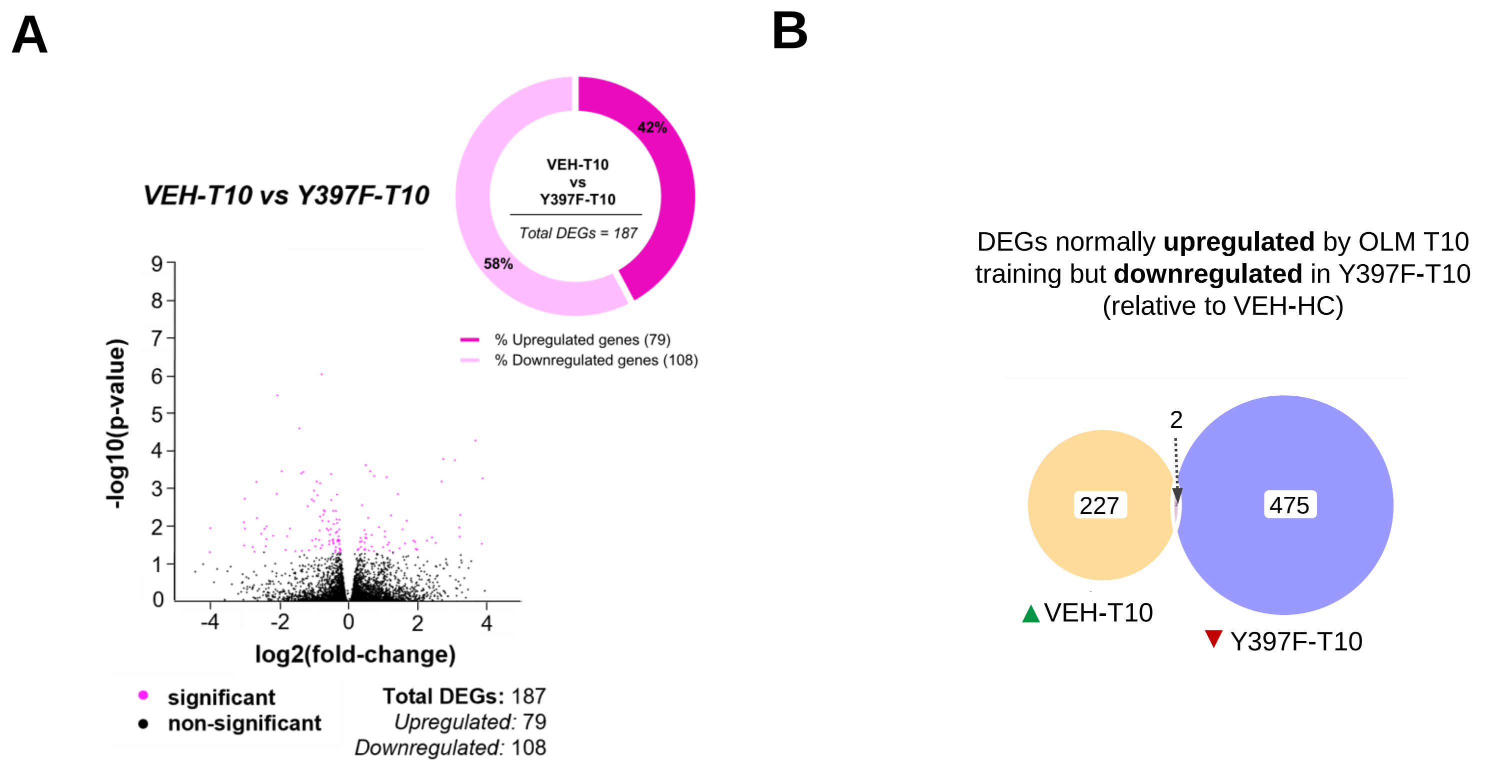

Figure S12. In vivo jetPEI transfection of CREST Y397 point mutants is sufficient to drive distinct DEGs relative to its corresponding threshold training period vehicle control.
(A) Volcano plot illustrates significance (y-axis) and magnitude (x-axis) for learning-induced DEGs ($p < 0.05$) for the Y397F-T10 experimental condition relative to the VEH-T10 control condition for a within threshold training period conditions DGE analysis (see **Table S13**). **(B)** Venn diagrams show the overlap in shared DEGs up-regulated in VEH-T10 (threshold OLM training) down-regulated in Y397F-T10. Number of significant DEGs ($p < 0.05$, relative to HC*)* (**Tables S14)** and overlap statistics (FDR-adjusted *p*-value from FET analysis; reported in **Table S6,** background 2) are annotated.

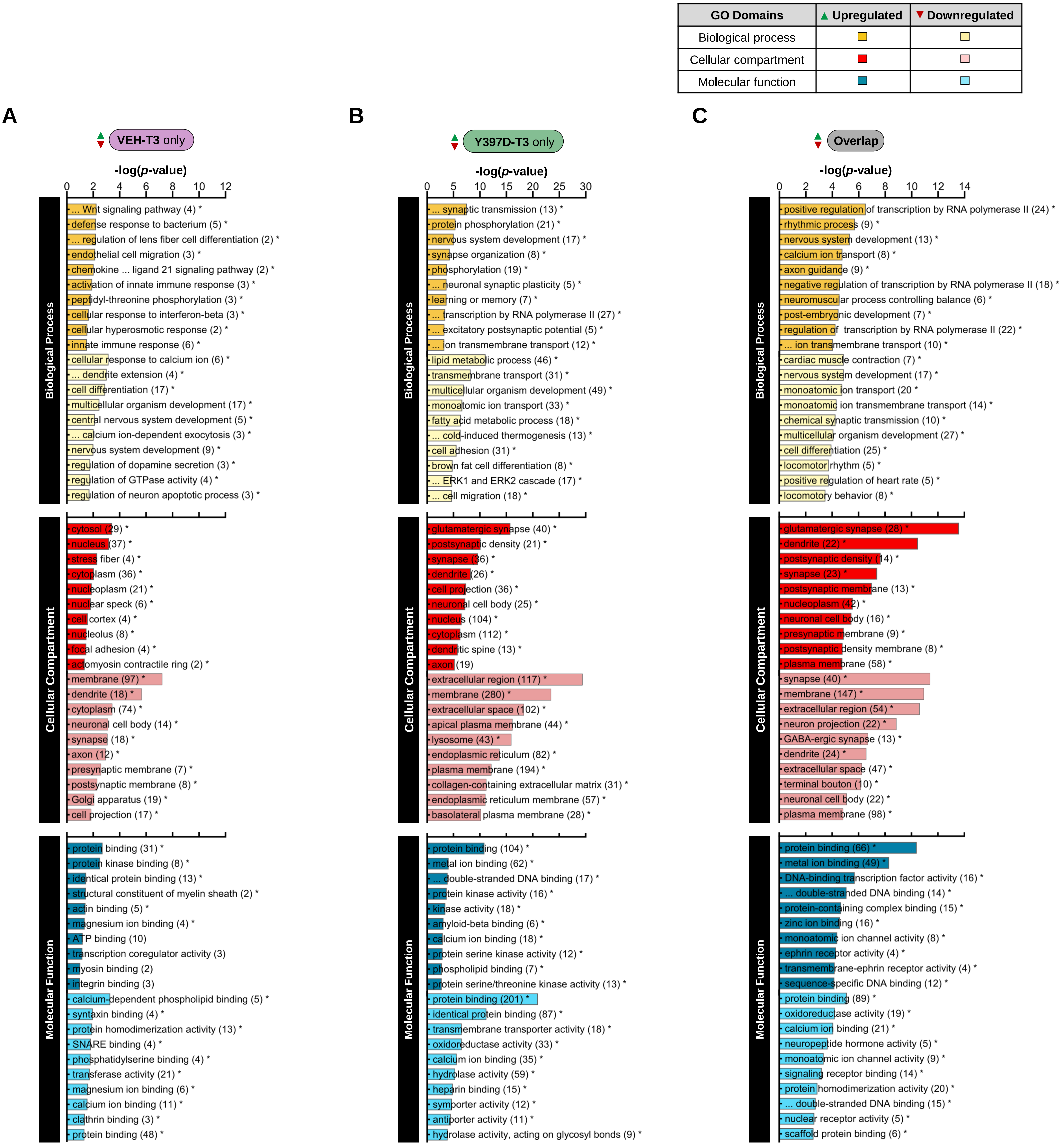

Figure S13.

DEGs affected by expression of the CREST Y397D point-mutant in a T3 OLM training period show distinct biological signatures.

Top 10 gene ontology (GO) terms from functional annotation and enrichment analysis of upregulated and downregulated DEGs ($p < 0.05$ cutoff) identified by the three distinct gene signature clusters in the two-way Venn diagram comparison of the VEH-T3 and Y397D-T3 conditions (**Figure 5H**) and categorized by biological process, molecular function and cellular compartment GO domains: **(A)** VEH-T3 only, **(B)** Y397D-T3 only, and **(C)** overlap. Fisher's exact p-value used to annotate significant gene-enrichment for an annotation term (* $p < 0.05$) (refer to **Table S6, Table S16** and **Table S17**). Upregulated GO terms = dark color scheme; and downregulated GO terms = light color scheme.

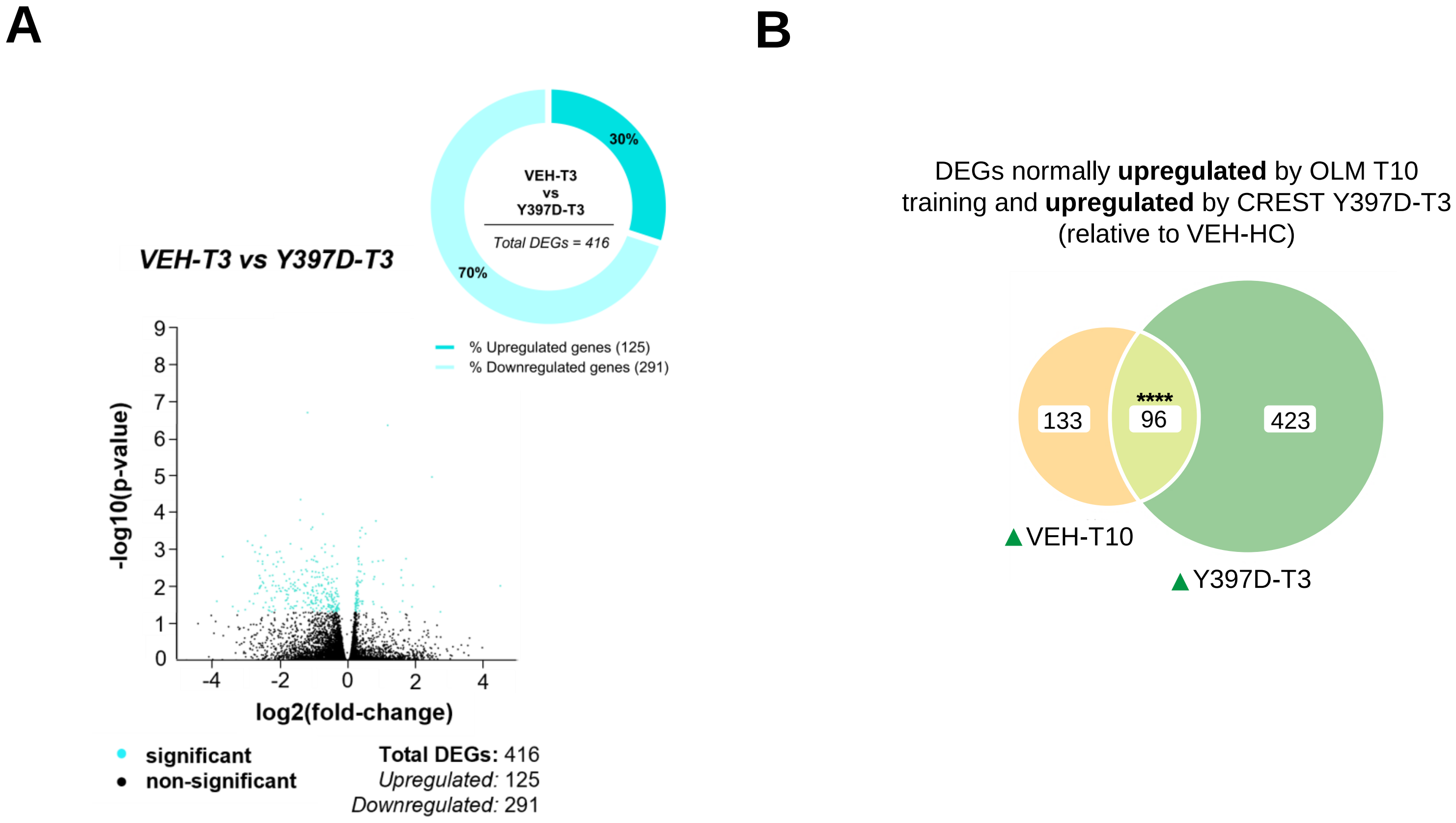

Figure S14. In vivo jetPEI transfection of the CREST Y39D point mutants is sufficient to drive distinct DEGs relative to its corresponding subthreshold training period vehicle control.
(A) Volcano plot illustrates significance (y-axis) and magnitude (x-axis) for learning-induced DEGs ($p < 0.05$) for the Y397-T3 experimental condition relative to the VEH-T3 control condition for a within subthreshold training period condition DGE analysis (see **Table S18**). **(B)** Venn diagrams show the overlap in shared DEGs up-regulated in both the VEH-T10 and the Y397D-T3 point-mutant condition (see **Table S19**). Number of significant DEGs ($p < 0.05$, relative to HC) *(***Tables S19**) and overlap statistics (FDR-adjusted *p*-value from FET analysis; reported in **Table S6,** background 2) are annotated.

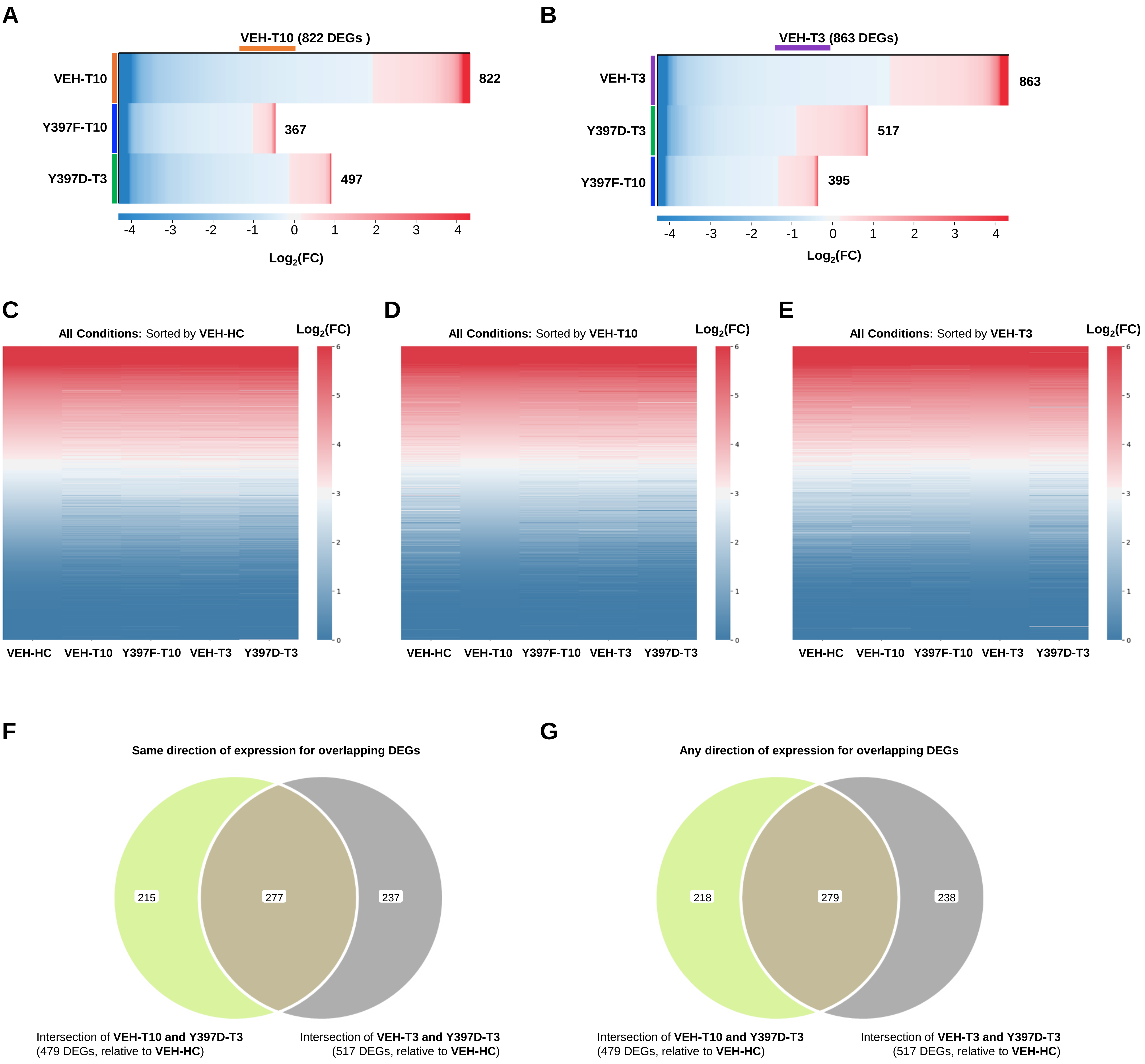

Figure S15.
Mutation of CREST Y397 alters learning-induced DEGs within distinct OLM training periods.
(A-B) Heatmaps compare the number and magnitude of learning-induced DEGs ($p < 0.05$) (relative to VEH-HC) in the Y397F-T10 and Y397D-T3 groups to DEGs exclusive to threshold (VEH-T10) or subthreshold (VEH-T3) OLM training period exposure, respectively (see **Table S20**). Venn diagram shows overlap between DEGs identified in the VEH-T10 (497) or VEH-T3 (517) control conditions for the Y397D-T3 phospho-mimic condition (each vs home cage) as identified in **(A)** and **(B)**, respectively. **(C-E)** Gene expression across all conditions. Heatmap representing the average of log-transformed gene expression levels within each condition. Genes are sorted according to their expression levels in the VEH-HC, VEH-T10 or VEH-T3 experimental conditions. The heatmap exhibits a high correlation of gene expressions across the groups, an observation commonly expected in biological studies since the majority of genes (90%-95% in our study) do not exhibit significant differential expression between conditions. **(F)** DEG entries from the Y397D-T3 condition that overlap and change in the same direction (i.e., up- or down-regulated in both Y397D-T3 and the training period control conditions). **(G)** DEG entries from the Y397D-T3 condition that overlap with either the VEH-T10 or VEH-T3 control condition lists are shown without requiring concordant direction of regulation (i.e., same gene is differentially expressed, regardless of log_2FC sign) (see **Table S21**).

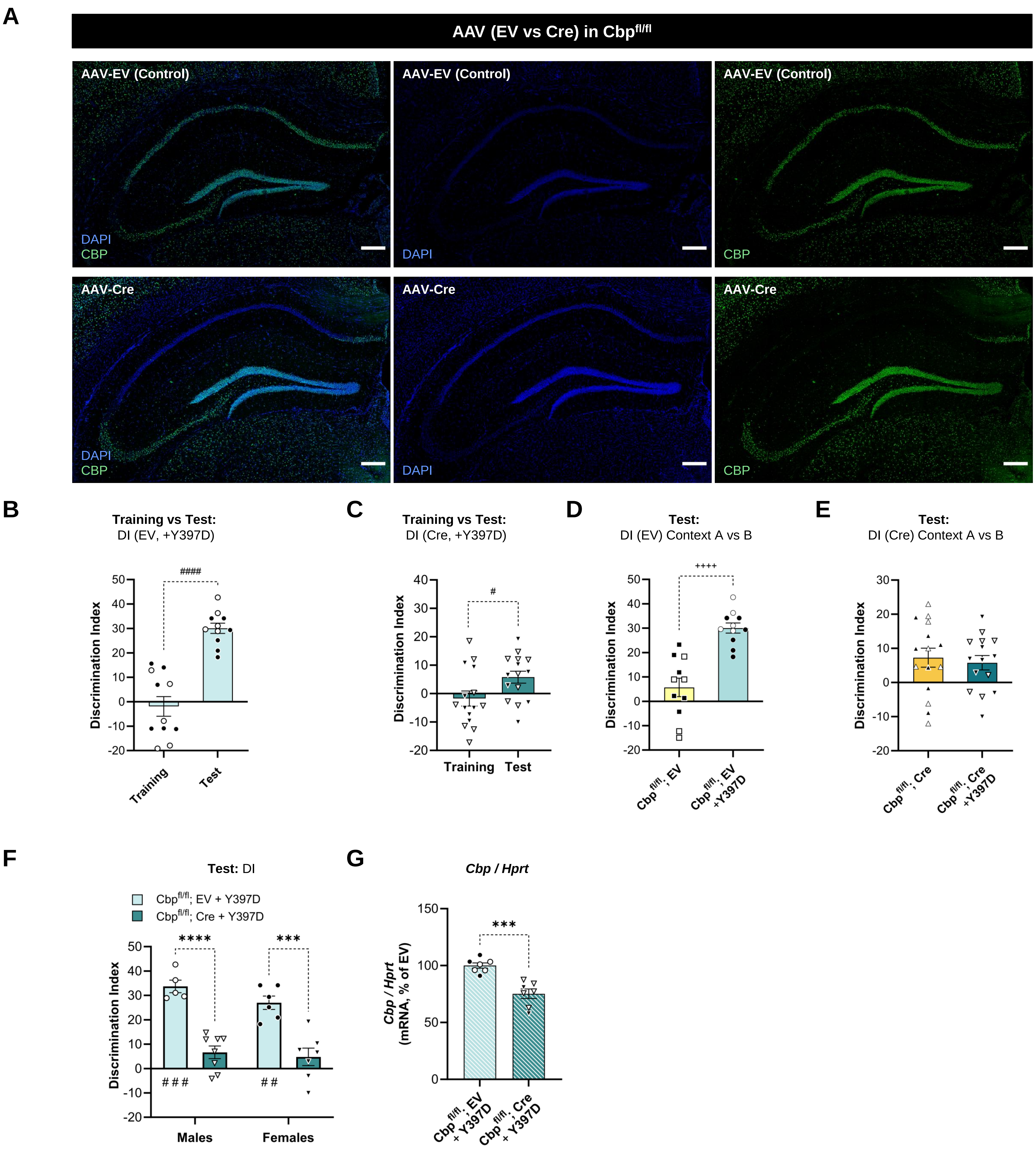

Figure S16.

OLM performance in Context A versus B and expression of Cbp JetPEI:Crest Y397D transfection.

(A) Representative immunofluorescence micrographs show CBP deletion after AAV-CMV-Cre infusion into the dCA1 of CBP$^{fl/fl}$ mice. The CBP (green) deletion was observed in brains injected with AAV-CMV-Cre, but not in brains injected with AAV-CMV-EV. Cells were counterstained with DAPI (blue). Scale bar: 250µm. **(B)** DI for *Cbp$^{fl/fl}$*; EV, +Y397D comparing test and training session in Context A (Student's t-test: t_{20} = 7.05, $^{####}$ p<0.0001) (**Figure 7H** and **J**). **(C)** DI for *Cbp$^{fl/fl}$*; Cre, +Y397D comparing test and training session in Context A (Student's t-test: t_{28} = 2.23, $^{#}$ p < 0.05) (**Figure 7H** and **J**). **(D)** DI for OLM testing session in *Cbp$^{fl/fl}$* mice with EV (Context B, squares) or EV, +Y397D infusion (Context A, circles) (Student's t-test, t_{20} = 5.62, $^{++++}$ p < 0.0001) (**Figure 7E** and **J**). **(E)** DI for OLM testing session in Cbp$^{fl/fl}$ mice with Cre (Context B, triangles) or Cre, +Y397D infusion (Context A, dark teal) (Student's t-test, t_{28} = 0.43, p = 0.67) (**Figure 7E** and **J**). **(F)** DI during OLM test session in Context A for comparison of *Cbp$^{fl/fl}$*; EV, +Y397D and *Cbp$^{fl/fl}$*; Cre, +Y397D conditions stratified by sex (Two-way ANOVA: effect of AAV condition, $F_{1,22}$ = 66.13, **** p < 0.0001) with pre-planned comparisons to corresponding training DI annotated (Student's t-test: Males (*Cbp$^{fl/fl}$*; EV +Y397D, pre-planned paired t-test, training x test: t_8=5.53, $^{###}$ p < 0.001) and (*Cbp$^{fl/fl}$*; Cre +Y397D, pre-planned paired t-test, training x test: t_{14}=1.76, p = 0.10); Females (*Cbp$^{fl/fl}$*; EV +Y397D, pre-planned paired t-test, training x test: t_{10} = 4.40, $^{##}$$p$ < 0.01) and (*Cbp$^{fl/fl}$*; Cre +Y397D, pre-planned paired t-test, training x test: t_{12} = 1.250, p = 0.24). No effect of sex x AAV condition interaction (Two-way ANOVA: $F_{1,22}$ = 0.64, p = 0.43). **(G)** Quantification of *Cbp* mRNA expression in the dCA1 collected after OLM testing session from Cbp$^{fl/fl}$; EV and Cbp$^{fl/fl}$; Cre mice with in vivo JetPEI-mediated transfection of Crest Y397D (N = 7/ group; Student's t-test: t_{12} = 5.22, *** p < 0.001). *Hprt* used as reference gene for mRNA normalization. OLM Behavior: n = 11-15 (5-8 males and females) /group. mRNA quantification: n = 7 (males and females) /group. Data presented as mean ±SEM. Males = white squares, triangles and circles. Females = black squares, triangles and circles.
