# Supplementary material for: The nBAF complex subunit CREST/SS18L1 regulates hippocampal memory processes via tyrosine 397 and histone acetyltransferase CBP
Source: Cell Rep. Author manuscript; Available in PMC 2026 May 20. (PMC13189212; doi:10.1016/j.celrep.2026.117158)
Supplement: Data_S1 [file NIHMS2168525-supplement-Data_S1.pdf]

Data S1. Original immunoblots and NetPhorest2.1 protein motif analysis output

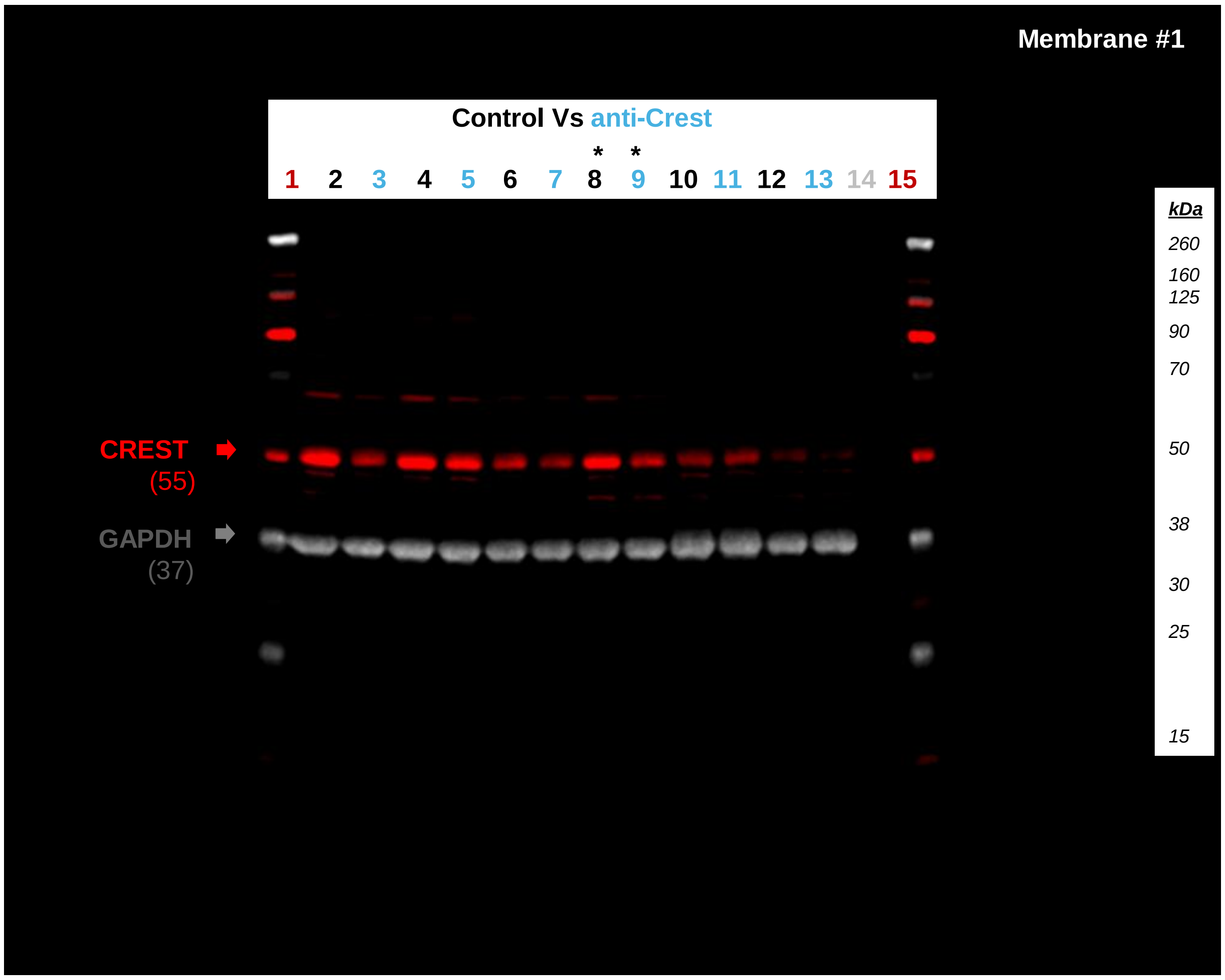

Source data (Figure 1A-B) : Full-length immunoblot for cropped representative image shown in **Figure 1A** and used for associated quantification of CREST (red, 700nm signal, IRDYE 680LT, 55kDa) and GAPDH (gray, 800nm signal, IRDYE 800CW, 37kDa) (**Figure 1B**) in protein lysate from dissected dCA1 tissue infused with a Control (black, even numbered lanes) or anti-Crest (blue, odd numbered lanes) morpholino oligo. *n* = 6 /condition. Lanes #8 and #9 (marked with asterisks) were cropped and used as representative images for **Figure 1A**. Lane #14 is empty. Both lanes #1 and #15 correspond to the Chameleon Duo Pre-stained Protein Ladder (kilodaltons, kDa; #928-60000) with protein band sizes annotated outside (right) of the immunoblot image margins.

A

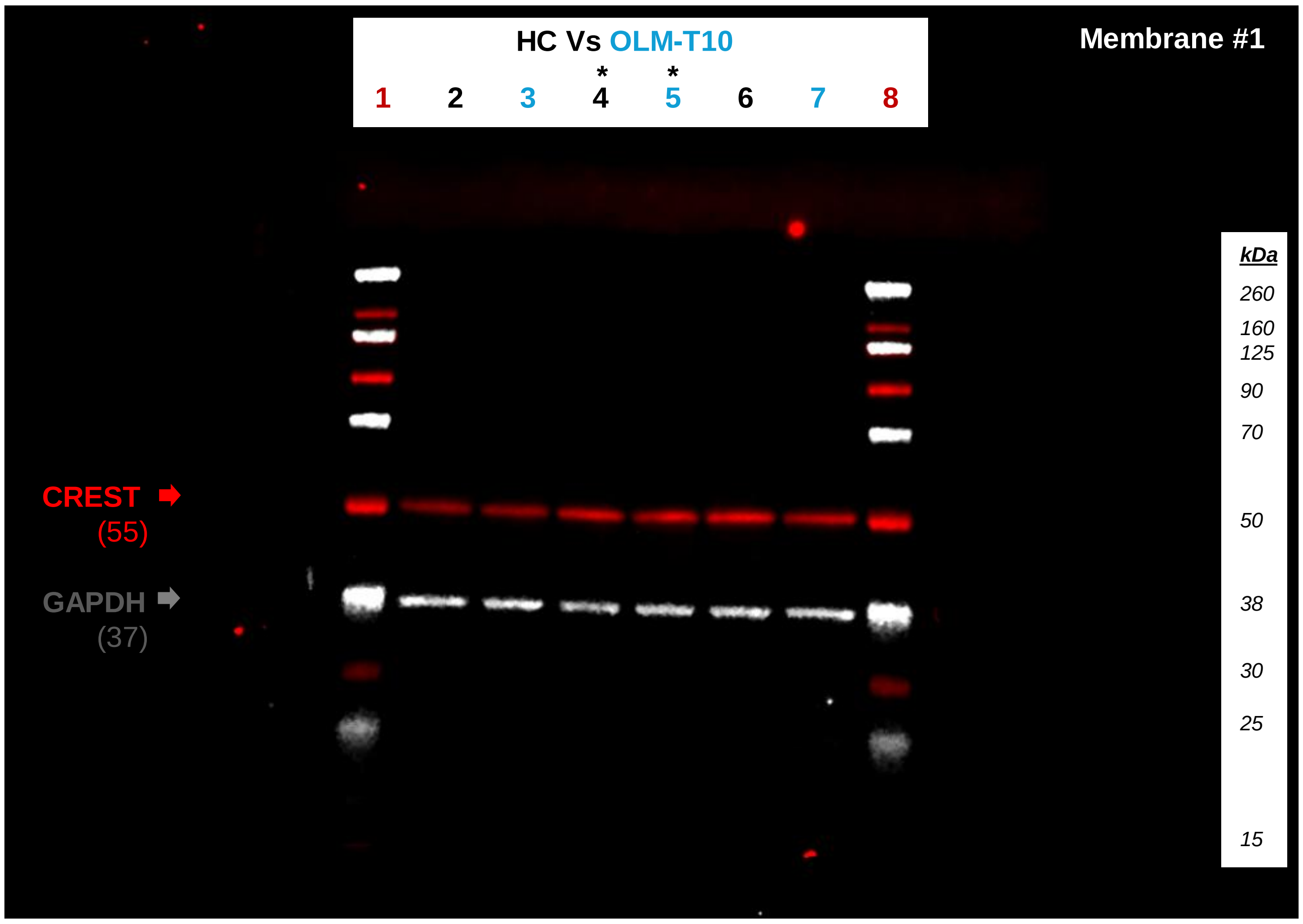

B

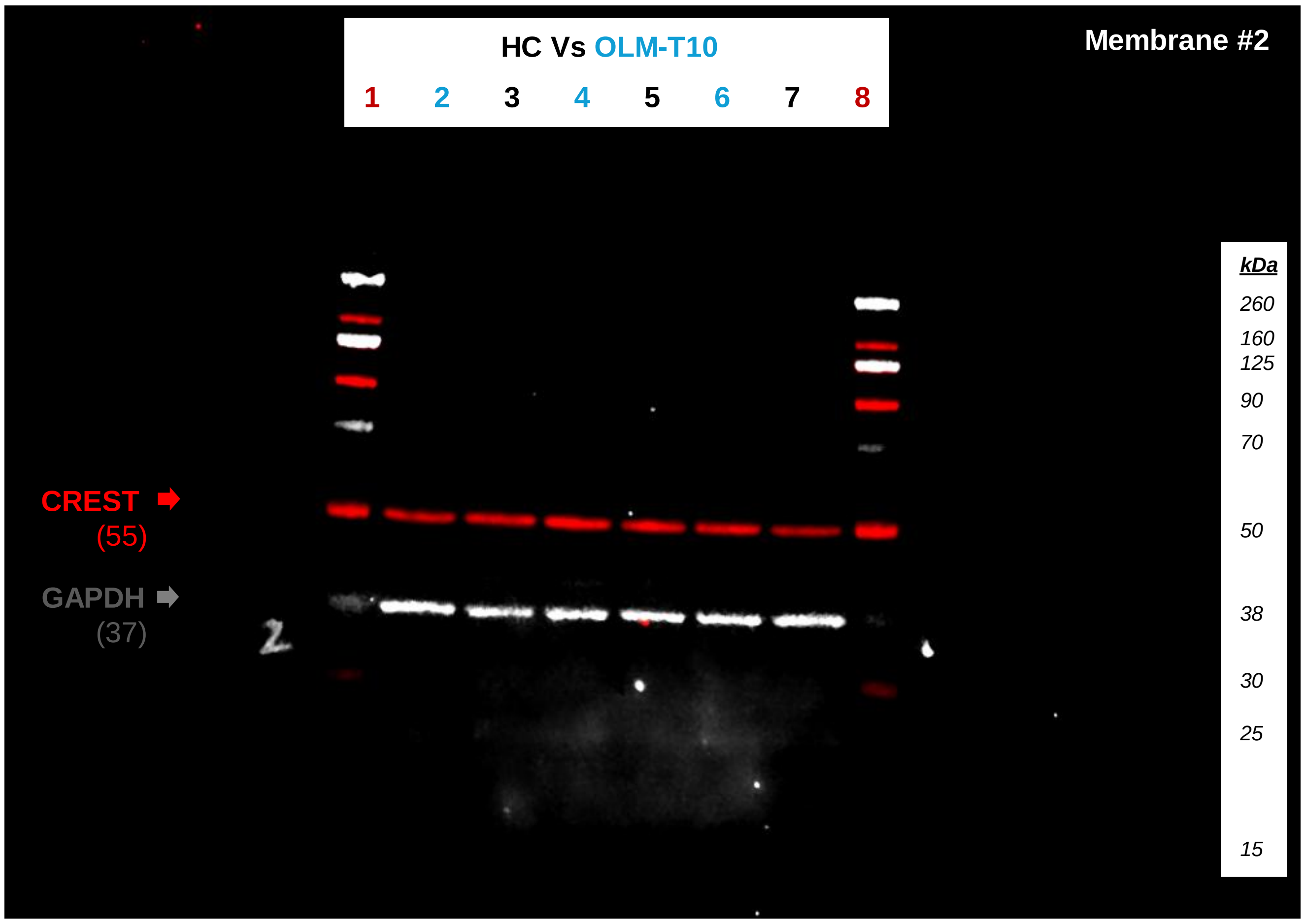

Source data (Figure 3D): (A-B) Full-length immunoblot used for associated quantification of CREST (red, 700nm signal, IRDYE 680LT, 55kDa) and GAPDH (gray, 800nm signal, IRDYE 800CW, 37kDa) (**Figure 3D**) in protein lysate from dissected dCA1 collected from home-cage (HC, black, even numbered lanes, Membrane #1; and odd numbered lanes, Membrane #2) and 1hr after OLM threshold training period (OLM-T10, blue, odd numbered lanes, Membrane #1; and even numbered lanes, Membrane #2)). *n*. Lane #1 and #8 of both membranes correspond to the Chameleon Duo Pre-stained Protein Ladder (kilodaltons, kDa; #928-60000) = 6 /condition with protein band sizes annotated outside of the immunoblot image margins. Lanes #4 and #5 of Membrane 1 (**A**) (marked with asterisks) were cropped for representative image in **Figure 3D**.

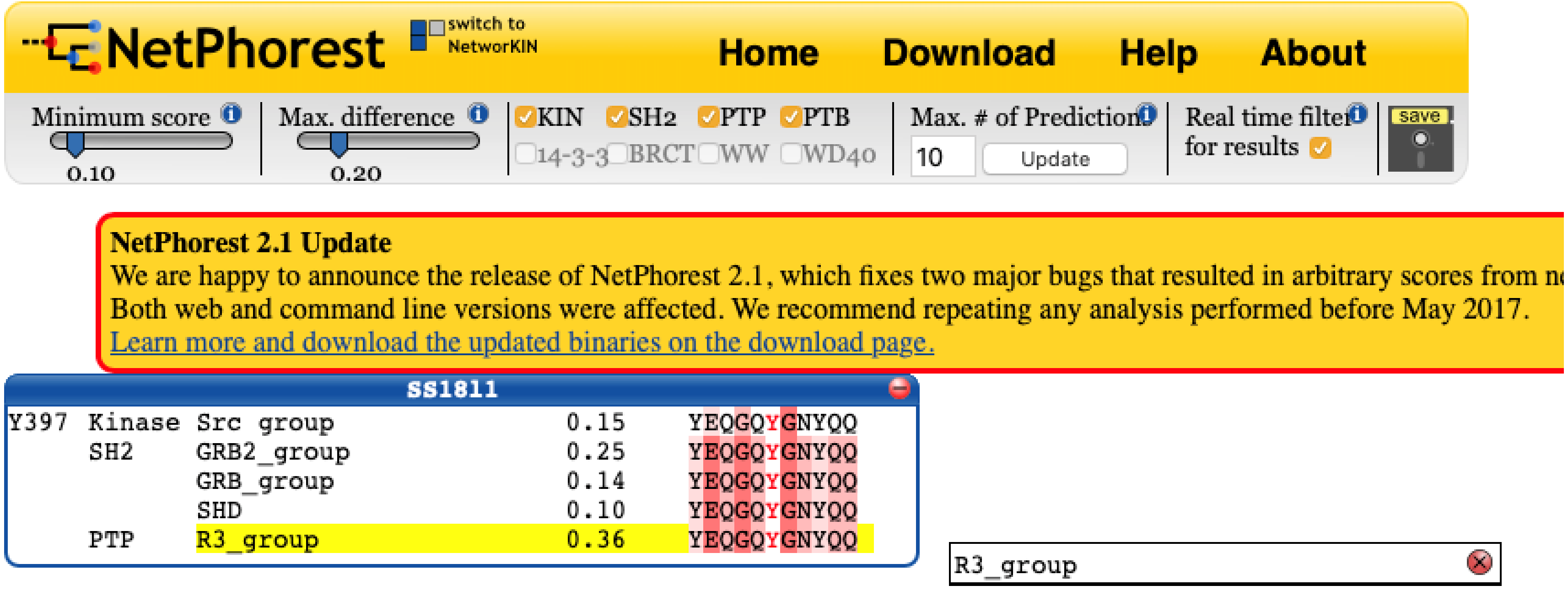

Source data (Figure 3G). Original output data from NetPhorest (machine-learning): http://netphorest.info/help.shtml
https://www.ncbi.nlm.nih.gov/pmc/articles/PMC6215708/
